# Identification of cellular genes and pathways important for tumorigenicity of hepatocellular carcinoma cell lines by proteomic profiling

**DOI:** 10.18632/oncotarget.21821

**Published:** 2017-09-27

**Authors:** Ali Zamani, Huahao Fan, Guangxiang Luo

**Affiliations:** ^1^ Department of Microbiology, University of Alabama at Birmingham School of Medicine, Birmingham, AL 35294, USA; ^2^ Department of Microbiology, Peking University Health Science Center School of Basic Medical Sciences, Beijing, 100191, China; ^3^ Current address: Department of Pathology and Laboratory Medicine, University of Pennsylvania, School of Medicine, Philadelphia, PA 19104, USA

**Keywords:** hepatocellular carcinoma, hepatitis virus, tumorigenicity, proteomics, HCC targets

## Abstract

Hepatocellular carcinoma (HCC) is the most commonly diagnosed malignancy of the liver. A more thorough understanding of HCC pathogenesis will provide novel targets for development of cancer drugs to effectively treat HCC. To further this goal, we carried out a proteomic profiling of HCC cell lines Huh-7.4 and Huh-7.5. These two cell lines were derived from subgenomic HCV RNA-replicating Huh-7 cells upon clearance of HCV RNA by antiviral drug treatment. Initially, the tumorigenicity of each cell line was determined and compared in parallel in the same immunedeficient mice. Strikingly, the Huh-7.4 cell line was able to induce tumors, whereas the Huh-7.5 cell line failed to do so, providing unique model systems for identifying cellular genes and pathways important for HCC development and progression. Subsequently, one-dimensional LC-MS/MS proteomic and bioinformatics analyses were performed in the hope of identifying unique cellular genes and pathways responsible for HCC tumorigenicity. Interestingly, a total of 130 cellular genes were found to be significantly up- or downregulated between these two cell lines (r>3 fold, P<0.001). Also, EIF (EIF2&4), mTOR/p70S6K, ERK5, and EGFR signaling pathways were significantly different. Overall, these results provide significant new information to shed light on the underlying biological processes involved in HCC development and progression.

## INTRODUCTION

Hepatocellular carcinoma (HCC) is the most commonly diagnosed malignancy of the liver, with a poor five-year survival rate (7%) due to its late presentation and resistance to chemotherapy. It ranks as the fifth most common cancer type and the third leading cause of cancer death worldwide [3]. It is a highly malignant tumor type, with average survival rate of less than 1 year and high recurrence rate after surgery (>70%). HCC is also the most rapidly increasing type of cancer, with annual deaths of more than 14,000 in the U.S. alone. The rapid increase in HCC incidence in the U.S. and other developed countries correlates with the prevalence of chronic hepatitis C virus (HCV) infection. Other major risk factors for HCC include hepatitis B virus (HBV), alcohol, nonalcoholic steatohepatitis (NASH), and aflatoxin B. However, the underlying molecular mechanisms for initiation and progression of HCC are unknown.

Genome-wide transcriptome profiling studies have identified a number of differentially expressed genes associated with HCC [1]. A big challenge is how to determine which cellular genes can serve as HCC biomarkers or therapeutic targets [2]. Another remaining question is whether the levels of mRNAs truly reflect their corresponding proteins [3]. Unlike genomic profiling, proteomic analysis directly determines the levels of protein expression, which is a better measurement of cellular functions. Accordingly, HCC proteomic profiling is the method of choice for determining the underlying molecular mechanism of HCC initiation, progression, and chemotherapy resistance. It is known that HCC development is associated with alteration of protein expression, which is exemplified by the HCC biomarker proteins alpha-fetoprotein and glypican-3 [4, 5].

In the past, proteomic studies on primary liver cancer were carried out using hepatic tumor cell lines, tissues, and patients’ sera. The overall goal of most proteomic studies was to identify novel biomarkers for HCC diagnosis and prognosis as well as therapeutic targets for drug discovery [6]. A number of proteomic analyses were recently reported by focusing on several HCC cell lines, such as MHCC, SMMC, HepG2, BEL7404, and Huh-7, in the hope of identifying unique HCC-associated proteins [7–11]. However, these cell lines do not support robust HCV infection and replication. Interestingly, the derivative sublines derived from Huh-7 cells were highly permissive to HCV infection and replication, including Huh-7.4 (unpublished results) and Huh-7.5 [12]. HCV is a major risk factor for HCC development. In the present study, we have profiled the tumorigenic potential of Huh-7.4 and Huh-7.5 cell lines in immunodeficient mice. Strikingly, inoculation of Huh-7.4 cells efficiently induced tumors, whereas Huh-7.5 cells failed to do so, providing a unique model to determine cellular factors important for HCC development. Proteomic profiling of these two cell lines has identified many differentially expressed proteins, and several signaling pathways involved in HCC development and progression.

## RESULTS

### Tumorigenicity of HCC cell lines Huh-7.4 and Huh-7.5

**Figure 1:**
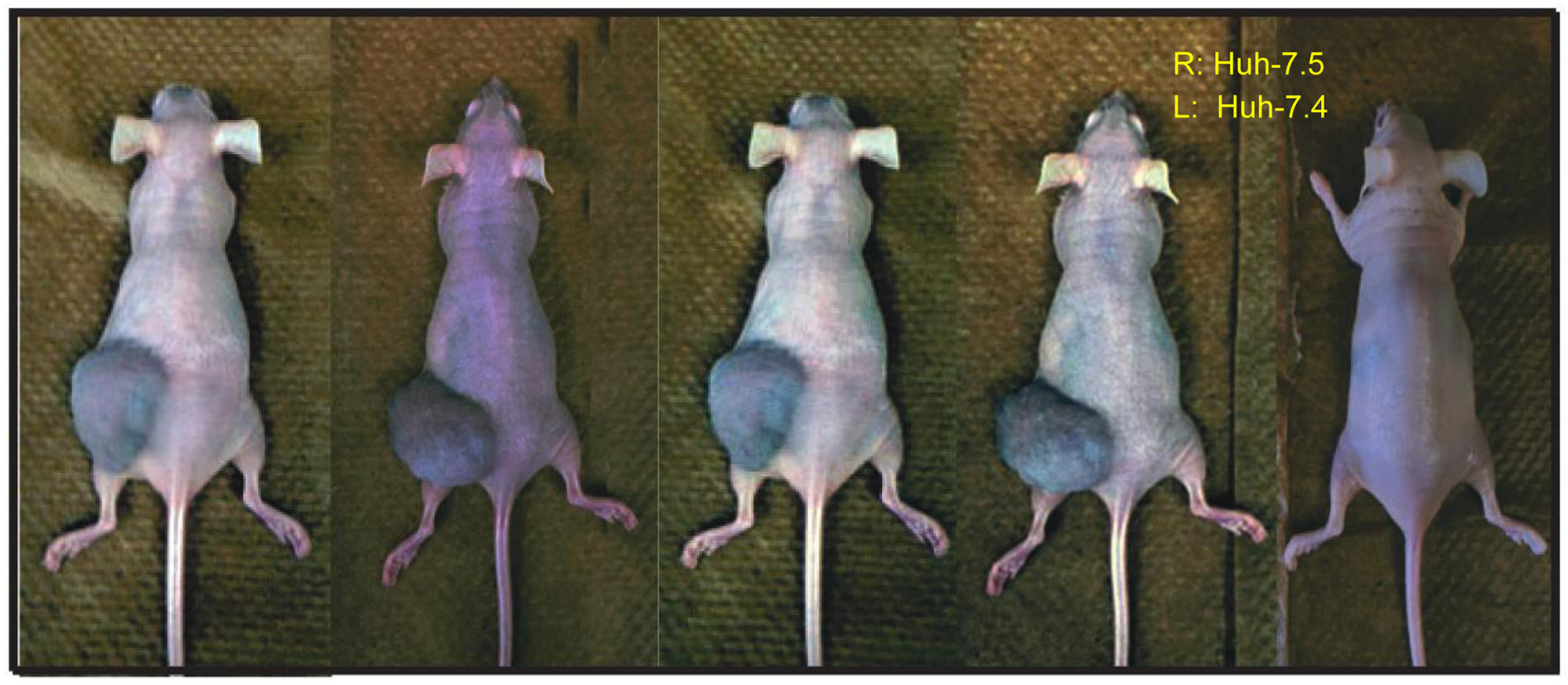
Tumorigenicity of Huh-7.4 and Huh-7.5 cell lines. A total of 3×10^6^ viable Huh-7.4 and Huh-7.5 cells in 100 μl of PBS (with Ca^2+^/Mg^2+^) were injected into the left and right flank of 5 nude mice (6 weeks old female), respectively. A total of 5 mice were used for the experimental condition. The tumor development was monitored weekly over a period of 6 weeks.

The Huh-7 cell line is widely used for the study of HCC and for screening potential therapeutics [13]. Although multiple HCC cell lines are available, only Huh-7 and its derivative variants are permissive to robust HCV infection and replication, making it an attractive model for determining the importance of HCV in HCC development and progression. However, HCV replication in the parental Huh-7 cells is inefficient [12]. Interestingly, its derivatives Huh-7.4 (unpublished results) and Huh-7.5, originating from HCV-replicating Huh-7 cells upon clearance of HCV by treatment with anti-HCV drugs and interferon, are highly susceptible to HCV infection and replication [12]. Therefore, tumorigenicity of these two cell lines was compared using immunodeficient nude mice (FOXn1-nude mice). To our surprise, Huh-7.4 cells developed tumors (4 out of 5), whereas Huh-7.5 failed to induce any visible tumors (Figure 1). This finding suggests that cellular genes and pathways differed between the two cell lines determine the outcomes of HCC development. It also provided an ideal system through which we could characterize the differences between these two cell lines in order to identify the tumorigenic mechanisms and factors that were inherent to the Huh-7.4 cell line.

### Proteomic profiling of HCC cell lines using LC-MS/MS analysis

**Figure 2:**
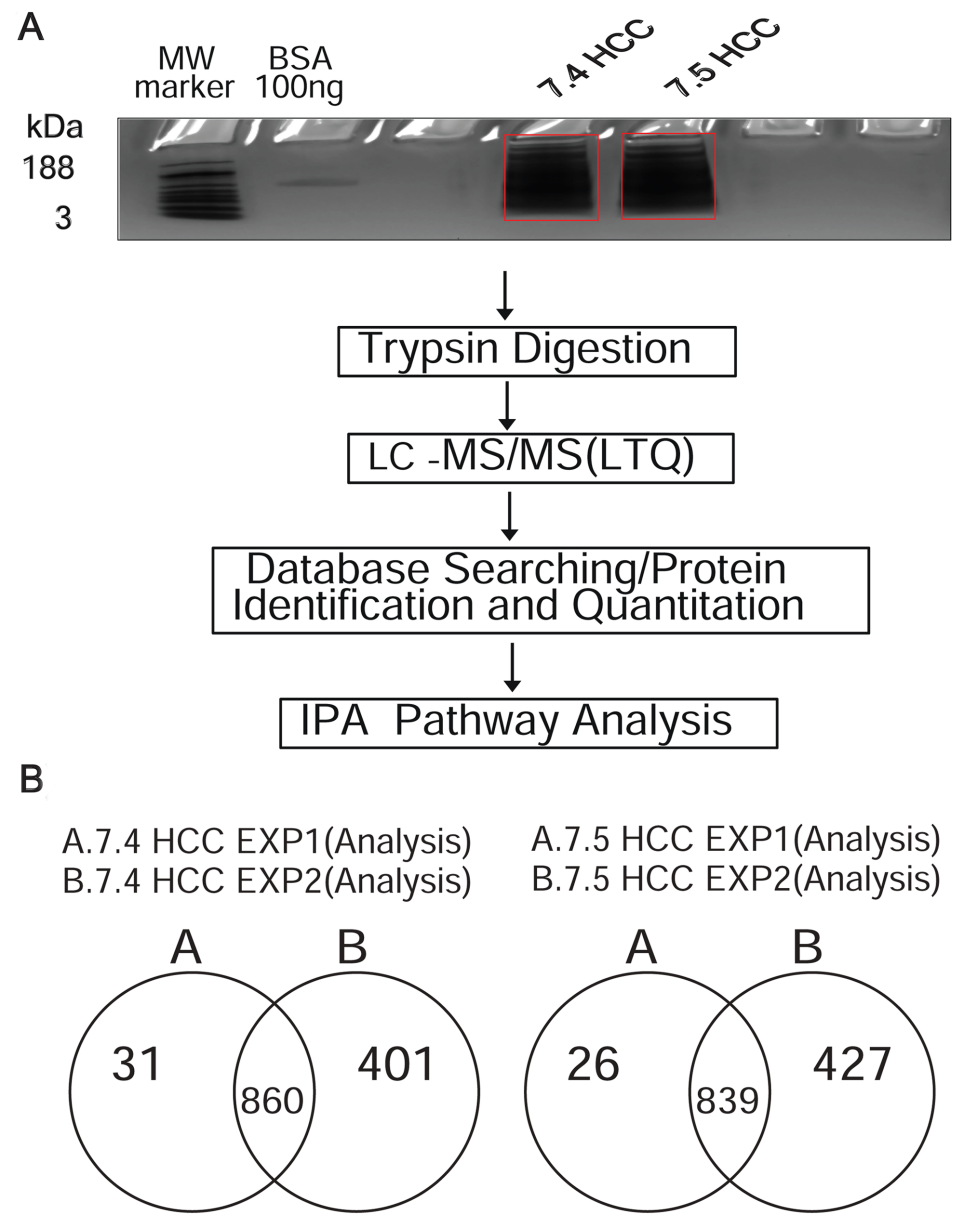
**(A)** Schematic overview of the experimental workflow. The workflow used in the IPA-conjugated proteomics analysis of Huh-7.4 and Huh-7.5 cell lines. A total of 20 μg proteins from each replicate were separated on short-stack gel, extracted and digested with trypsin. Peptides were then subjected to LC-MS/MS. The resulting spectra were searched for identification and quantification. The identified and quantified proteins were then analyzed using Ingenuity Pathways analysis software (IPA). **(B)** Number of common proteins identified in Huh-7.4 and Huh-7.5 by LC-MS/MS for two independent experiments. A list of proteins using database searching were generated, mapped and analyzed by IPA in duplicate performed in the same manner for each cell line.

At molecular level, the eight hallmark phenotypes of cancer cells (ability to sustain proliferation, resistance to cell death, escaping growth suppressors, the tendency to invade and metastasize, induction of angiogenesis, ability to evade immune system surveillance, and use of multiple sources of energy) [14] are regulated by differential protein expression and pathway activation. Therefore, differences in protein expression may account for the increased tumorigenicity of the Huh-7.4 cell line. To test this hypothesis, we performed a the LC-MS/MS analysis and compared the proteomic profiles (acquired spectral count) of the Huh-7.4 and Huh-7.5 cells. As shown in Figure 2A, two independent analyses (performed in duplicate) were subjected to fraction digestion followed by LC-MS/MS, and a total of 1035 and 1462 proteins were identified in experiment 1 and 2, respectively. Many of these proteins contained two or more unique peptide identifiers (Supplementary excel sheet file 1). After mapping data to the Ingenuity Pathway Analysis (IPA) knowledge base (KB), for the Huh-7.4 cell line, 860 proteins were identified in both experiments, yielding a reproducibility of 68%. For the Huh-7.5 cell line, 839 proteins were common to all biological replicates (66% reproducibility) (Figure 2B). The datasets generated from the duplicate runs (860 and 839 proteins) are listed in Supplementary excel sheet file 2, and were analyzed further.

### Classification of MS/MS-identified proteins by cellular localization and functions

**Figure 3:**
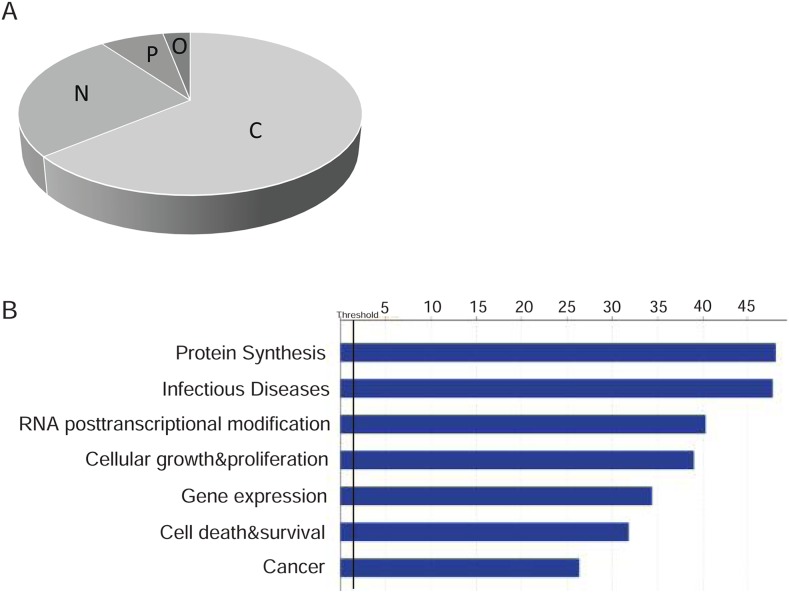
**(A)** Cellular localization of proteins identified in Huh-7.4 and Huh-7.5 by LC-MS/MS for two independent experiments. Overlapped proteins for each cell line were identified and classified by the cellular location of each. C: Cytoplasm, N: Nucleus, P: plasma membrane, O: other (Transporter and growth factors). **(B)** IPA biofunction analysis on the differentially expressed proteins in Huh-7.4 and Huh-7.5 cell lines. Biofunction analysis conducted through Ingenuity Pathway Analysis (IPA). Pathways associated with protein synthesis, infectious diseases, RNA post-transcriptional modification, cellular growth and cancer were altered in both cell lines and reached statistical significance (Z score >2.0) in Ingenuity Pathway Analysis.

The Huh-7.4 and Huh-7.5 datasets, containing 860 and 839 entries respectively, were analyzed independently using IPA Ingenuity software. The IPA database can provide details about the subcellular localization and molecular/cellular function, as well as predict pathway activation and potential therapies for a set of proteins. We found that a large portion of the proteins expressed in both cell lines are localized to the cytoplasm (~64%). Approximately 26% and 7% of proteins are predicted to be localized in the nucleus and plasma membrane, respectively. About 3% of proteins are growth factors or transporters and are classified as extracellular proteins (Figure 3A). Most of these proteins were assigned to functional categories such as cellular growth and proliferation, cell death and survival, gene expression and protein synthesis, and RNA/post-transcriptional modification (Figure 3B). Supplementary excel sheet file 3 shows detailed high-resolution bar graphs and the classification of each of these proteins from each cell line. As expected based on the nature of the cells, infectious diseases (e.g. viral infection) and cancer were the top diseases associated with both cell lines (Figure 3B). Supplementary excel sheet file 3 classifies the proteins by infectious diseases and cancer.

### Protein interaction and signaling pathway analysis

**Figure 4:**
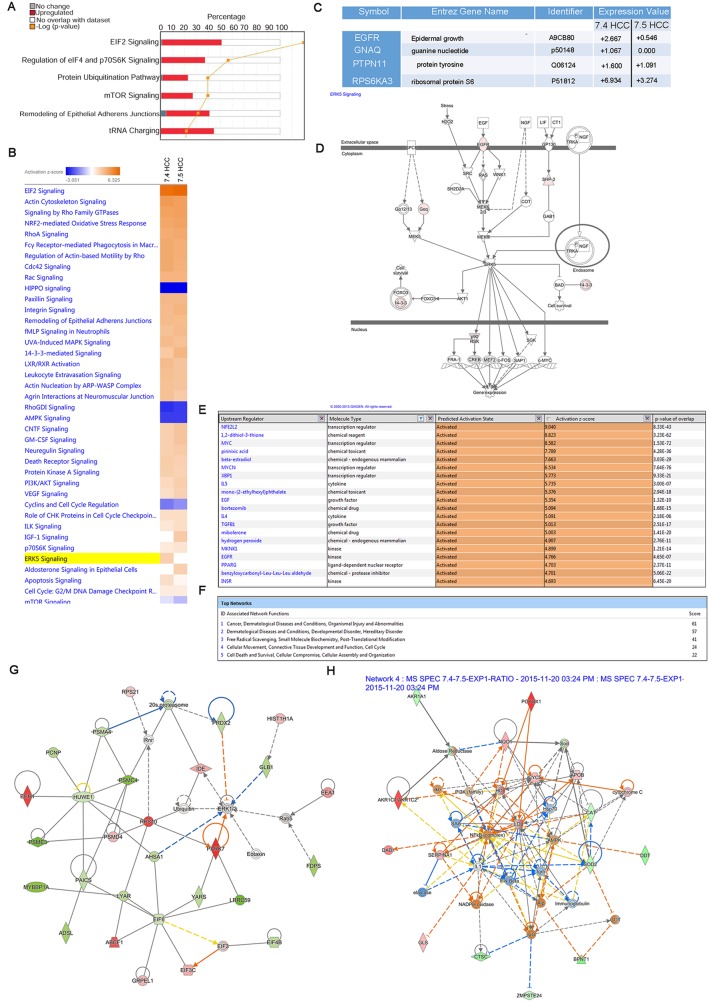
**(A)** Canonical pathway analysis of expressed proteins in in Huh-7.4 over Huh-7.5. The most statistically significant canonical pathways identified in the Huh-7.4 cell line are listed according to their p value (-Log) (orange line) and the ratio of list proteins found in each pathway over the total number of proteins in that pathway (Ratio, red bars). **(B and C)** Heat map analysis of canonical pathways upregulated in Huh-7.4 over Huh-7.5 cell line. Selected heat map and paired table demonstrating upregulation of the components of ERK5 pathway in the Huh-7.4 cells. **(D)** IPA prediction analysis of higher level of tumorigenesis in Huh-7.4 vs Huh-7.5 HCC cell lines. IPA analysis identifies a network of proteins that show how ERK5 upregulation downstream of EGF binding at its receptor region could lead to differential expression of critical genes in cell survival or growth. **(E)** Upstream regulators of differentially expressed proteins in Huh-7.4 cell line. Based on differential protein expression profile in Huh-7.4, IPA's regulator effects algorithm connected the upstream regulators to downstream functions to generate regulator effects hypotheses with predicted activation of upstream transcription regulators such as NFE2L2, MYC and XBP1. **(F)** Network analysis of differentially overexpressed proteins in Huh-7.4 over Huh-7.5. IPA network analysis identified the most significant overexpressed protein in the Huh-7.4 cell line compared to Huh-7.5, generating networks of these proteins based on their connectivity and assigned a score. **(G)** Cancer network originating from upregulated expression of proteins in Huh-7.4 cells. **(H)** Network of free radical scavenging/post-translation modification in Huh-7.4 over Huh-7.5. The intensity of the node's red color indicates the degree of upregulation, while the intensity of the green color indicates the degree of downregulation. The blue color indicates critical members of the network. The node shapes denote enzymes, phosphatases, kinases, peptidases, G-protein coupled receptor, transmembrane receptor, cytokines, growth factor, ion channel, transporter, translation factor, nuclear receptor, transcription factor and other entities.

To investigate cellular factors responsible for the difference in tumorigenesis between the Huh-7.4 and Huh-7.5 cells shown in xenograft model studies, we calculated the ratio of spectral expression of Huh-7.4 over Huh-7.5 using a cutoff of 3 for the average ratio (fold of change) and analyzed the differential protein expression in these cell types. This cutoff was chosen to achieve high reproducibility in proteomic data by reducing the false positive rate to around 7% [15]. IPA analysis of the Huh-7.4/Huh-7.5 ratio revealed ~132 proteins in each experiment that were either up- or downregulated using this cutoff. The ten proteins with the highest magnitude change (up or down) are summarized in Table 1 and Supplementary excel sheet file 4. To gain an understanding of how these identified proteins interact biologically, we performed canonical pathway analysis on this refined list of proteins of interest in IPA. The results of this analysis showed that the top pathways associated with these proteins were eukaryotic translation initiation factors (EIF2 and EIF4), mTOR/p70S6K signaling, ubiquitination, and remodeling of epithelial adherence junctions (Figure 4A and detailed high-resolution image in Supplementary excel sheet file 4). The EIF complex is known to be important for cancer initiation, progression, and protein translation. Heat map analysis of the canonical pathways activated in these cells showed upregulation of the ERK5 pathway in the Huh-7.4 cells. This was predicted based on the upregulation of EGFR and GNAQ (Figure 4B&4C). Interestingly, these proteins/pathways are known to be associated with cancer and cell survival. In another approach, we used the IPA regulator effects function to build a hypothesis regarding the enhanced tumorigenicity in Huh-7.4 versus Huh-7.5 cells. This tool generated a network connecting the predicted upstream regulators to the dataset, and then to the downstream diseases and functions. In agreement with our pathway analysis, this “regulator effect” analysis predicted that EGF signaling would play a significant role in the tumorigenic potential of Huh-7.4 cells (Figure 4D). IPA analysis predicted that the most active upstream regulators (having a Z-score higher than 2) were the NFE2L2, MYC, MYCN, and XBP1 transcription factors, IL-4 and IL-5 cytokines, EGF and TGF-B growth factors, and MKNK1, EGFR, and INSR kinases (Figure 4E). Interestingly, NFE2L2 has already received attention as a potential therapeutic target for HCC treatment [1]. Network analysis of the differentially expressed proteins revealed activation of cancer, cellular movement, cell cycle, free radical scavenging, cellular growth, and proliferation networks (Figure 4F). Examples of two of these networks (cancer and free radical scavenging/post-translation modification), with their representative molecules, are shown in Figures 4G and 4H. This analysis also revealed higher expression of cancer biomarkers such as KRT8 (breast cancer prognosis), FN1 (pancreatic cancer), NQ1 (lung cancer), and PSDM4 (liver cancer) in the Huh-7.4 cell line. Using the drug screening tool in IPA, which suggests possible therapies to target the activated networks, we obtained a list of potential drugs to use against Huh-7.4 cells. As shown in Supplementary PDF file 5, most of the suggested therapies are currently being used to treat different types of cancer (leukemia, ovary, and colon cancer) but have not yet been tested against liver cancer.

**Table 1:** Summary of ten proteins with the highest magnitude change (up- or downregulated) in Huh7.4 over Huh7.5 cell line

Symbol	Entrez gene name	Identifier	Expression value	Location	Type(s)
		UniProt/Swiss-Prot Accession	Exp fold change		
PARK7	parkinson protein 7	Q99497	↑13.686	Nucleus	enzyme
SPATS2L	spermatogenesis associated,serine-rich 2-like	Q9NUQ6	↑13.686	Nucleus	other
IQGAP1	IQ motif containing GTPaseactivating protein 1	P46940	↑12.709	Cytoplasm	other
ATP2B1	ATPase, Ca++ transporting,plasma membrane 1	P20020	↑9.776	Plasma Membrane	transporter
RRM2	ribonucleotide reductase M2	P31350	↑9.776	Nucleus	enzyme
PCYOX1	prenylcysteine oxidase 1	Q9UHG3	↑9.776	Cytoplasm	enzyme
RPS10	ribosomal protein S10	P46783	↑8.798	Cytoplasm	other
ECH1	enoyl CoA hydratase 1,peroxisomal	Q13011	↑8.798	Cytoplasm	enzyme
SQSTM1	sequestosome 1	Q13501	↑8.309	Cytoplasm	transcription regulator
ABCF1	ATP-binding cassette, sub-family F(GCN20), member 1	Q2L6I2	↑7.821	Cytoplasm	transporter
ALDOC	aldolase C, fructose-bisphosphate	P09972	↓-14.321	Cytoplasm	enzyme
PRKDC	protein kinase, DNA-activated,catalytic polypeptide	P78527	↓-12.276	Nucleus	kinase
PSME3	proteasome (prosome, macropain) activatorsubunit 3 (PA28 gamma; Ki)	P61289	↓-12.275	Cytoplasm	peptidase
CTNNB1	catenin (cadherin-associated protein),beta 1, 88kDa	B5BU28	↓-10.229	Nucleus	transcription regulator
LRRC59	leucine rich repeat containing 59	Q96AG4	↓-10.229	Cytoplasm	other
PSMC4	proteasome (prosome, macropain)26S subunit, ATPase, 4	P43686	↓-8.184	Nucleus	peptidase
MVD	mevalonate (diphospho) decarboxylase	P53602	↓-8.184	Cytoplasm	enzyme
KRT1	keratin 1, type II	P04264	↓-7.161	Cytoplasm	other
PUS7	pseudouridylate synthase 7 (putative)	Q96PZ0	↓-7.161	Other	other
MYBBP1A	MYB binding protein (P160) 1a	Q9BQG0	↓-7.161	Nucleus	transcription regulator

The top ten proteins with the highest magnitude change (up- or downregulated) in Huh-7.4 over Huh-7.5 are summarized. The main functions of these proteins were related to cancer, cellular movement, cell death and survival, cellular assembly and organization, connective tissue development and function, and infectious diseases.

### Validation of LC/MS/MS data

**Figure 5:**
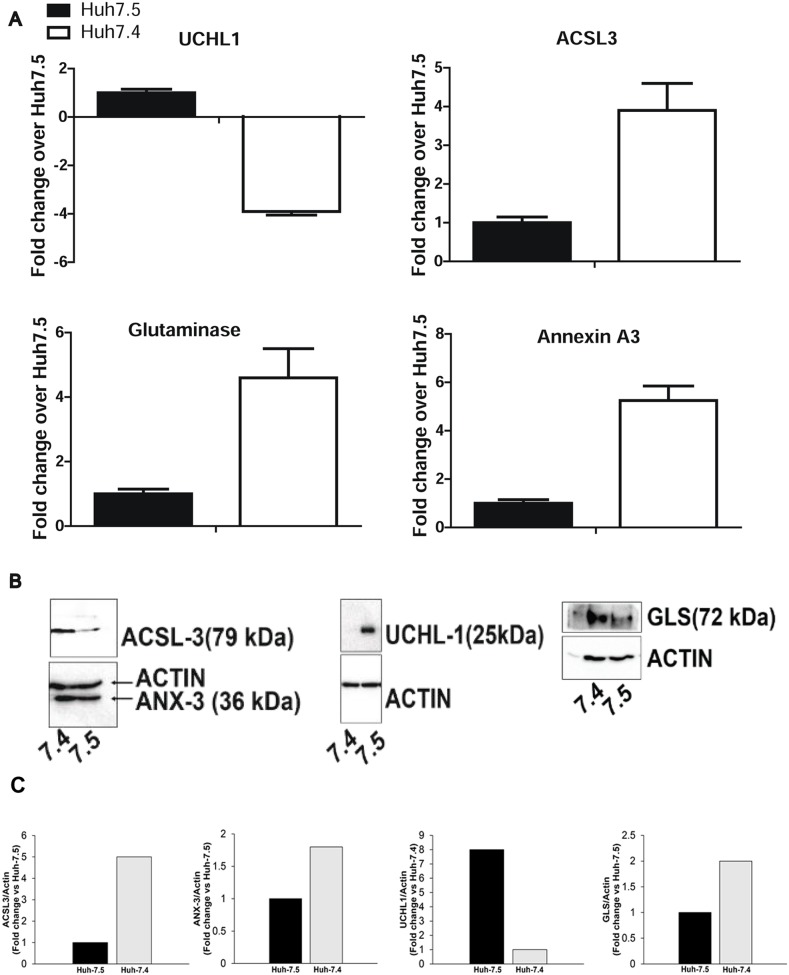
**(A)** Expression analysis of selected proteins from two mass spec experiments. The average MS/MS spikes from two experiments for representative proteins were analyzed and plotted against the number of spikes in the Huh-7.5 cell line. **(B)** Western blot analysis of selected proteins in Huh-7.4 and Huh-7.5 cells. Immunoblotting was used to confirm the upregulation/downregulation of the above representative proteins identified by high throughput MS/MS spectrometry in Huh-7.4 and Huh-7.5. **(C)** Quantification of selected proteins in B by densitometry analysis.

From the list of proteins that appeared to be differentially expressed between Huh-7.4 and Huh-7.5 cell lines, GLS (glutaminase kidney isoform, mitochondria), ANXA3 (Annexin A3), ACSL-3 (long chain fatty acyl-coA synthetase 3), and UCHL-1 (Ubiquitin C-terminal hydrolase-L1) proteins have previously been connected to HCC and cancer in general [16, 17]. IPA analysis also predicted that these proteins played a role in cancer, free radical scavenging, and amino acid metabolism networks. To validate our mass spectrometry data, we confirmed the levels of GLS, ANXA3, ACSL3 and UCHL-1 expression by Western blot. It was shown that Huh-7.4 cells express higher levels of GLS, ANXA3 and ACSL3, and virtually undetectable UCHL1 (Figure 5A). These findings are consistent with the results from proteomic analysis, showing 3-fold higher expression of GLS, ANXA3 and ACSL3 and a 3.9-fold lower expression of UCHL1 in Huh-7.4 versus Huh-7.5 cells (Figure 5B).

## DISCUSSION

HCC is one of the most malignant types of cancer due to its often late diagnosis and limited treatment options. A more thorough understanding of the fundamental mechanisms and molecular pathways involved in HCC initiation and progression is the key to discovery and development of novel drugs for prevention and treatment of HCC. Proteomic approaches have greatly contributed to the identification of biomarkers and the underlying tumorigenic mechanisms for many different types of cancer. Although there have been numerous reports on proteomic profiling of HCC cell lines, none of them would recapitulate HCV infection. Given that HCV is a major risk factor for HCC, HCV-permissive HCC cell lines are important model systems for the discovery of novel biomarkers with the goal of early detection and diagnosis. These cell lines will also be important for unraveling critical protein changes triggered by HCV infection, providing insights into effective therapeutic approaches for HCC patients. In this study, a high-throughput one-dimensional LC-MS/MS proteomic strategy was used to identify differences in protein expression between two highly permissive HCV hepatoma cell lines (Huh-7.4 and Huh-7.5). Interestingly, the two cell lines exhibited opposing phenotypes in tumorigenic potential. Both cell lines originated from parental Huh-7 cells in which a replicating HCV RNA of genotype 2a was cured by antiviral drugs. Proteomic profiling of these two cell lines with similar genetic background will be the method of choice for identifying alteration of cellular gene expression, a process important for HCC development and progression.

Proteomic analysis revealed ~800 reproducible proteins in these two HCC cell lines. The experiments were performed on two independent biological replicates and showed more than 60% reproducibility between the two runs. This is an acceptable level of overlap between the biological replicates due to the nature of MS/MS, where different peptides are selected and ionized in each run [18]. Using the IPA platform to execute our proteomic analysis, we were able to identify up- or down-regulated expression of genes among these two HCC cell lines. It was found that 130 proteins were significantly altered in their abundance (r>3 fold, P<0.001) between Huh-7.4 (resulting in tumor development) and Huh-7.5 (non-tumorigenic control). Among them, 25 proteins were significantly overexpressed in the Huh-7.4 cell line compared to those in the non-tumorigenic Huh-7.5 cell line (r>5 fold). Of them, PARK7 [19], IQGAP1 [20], SPATS2L and RRM2 [21], SQSTM1(p62) [22], AKR1C1 [23], KRT8 [24], ANXA7 [25], NQO1[26], SERPINA1[27], GLS [28] ANXA3 [29, 30], ACSL3 [31], and the tumor suppressor UCHL1 [32] are known to be associated with HCC initiation, progression, metastasis, and drug resistance. We validated differential expression of ACSL3, GLS, UCHL1 and ANXA3 through WB analysis.

Analyses such as molecular and cellular function, canonical pathway, network enrichment, biomarker, and drug screening were performed on the differentially expressed proteins using IPA. According to the cellular function analysis, we observed higher cell proliferation, increased protein synthesis and translation, and lower cell death and apoptosis in the Huh-7.4 cell line. This is not surprising since increases in translation and the overall rate of protein synthesis are characteristics of many cancers [33]. The biofunction analysis indicated that the primary diseases or disorders associated with proteins in Huh-7.4 cells were viral infection and cancer initiation and progression. In addition, the top canonical pathways related to 40% of the differentially expressed genes were EIF and mTOR signaling. In cancer, mTOR activation, induction of the EIF complex, and cooperation between these two pathways are known to be important for initiation of particular types of protein synthesis that influence cancer progression or confer resistance to treatment [34]. Interestingly, it has been established that EIFs (specifically EIF2 subunits) are required to initiate IRES-mediated translation of viral and cellular proteins in host cells [35]. Therefore our IPA analysis suggests that higher levels of EIF in Huh-7.4 cells not only predisposes them to cancer development, but also plays a critical role in HCV replication.

Our network analysis revealed several similar network characteristics between the two cell lines, as expected due to their common parental Huh-7 cell line. However, the ERK5 signaling pathway, and proteins in this network such as EGFR and RPS6KA3, showed higher levels of activation in Huh-7.4 than in Huh-7.5 (r>4 fold and r>2 fold, respectively). This pathway regulates cell proliferation and cancer cell transformation downstream of EGF stimulation [36], and proteins in this pathway are constitutively active in several human malignancies [37, 38]. Thus ERK5 and EGFR hyperactivation may also contribute to development of HCC.

This study was able to identify several candidate biomarkers, such as KRT8, FN1, NQO1 and PSMD4 [39], and also suggesting treatment options such as cytarabine, cetuximab, AE788, and L19-IL2 (a monoclonal antibody-cytokine conjugate currently being used to treat other types of cancer) can be potential therapeutics for HCC. Future studies are warranted to validate the above targets and potential drugs in the aforementioned xenograft HCC model (Figure 1).

In summary, this is the first proteomic analysis of HCV-permissive HCC cells, and will serve as an initial step for the next generation of comprehensive studies to analyze the proteomic profile of HCV-mediated HCC. In this study, we compared the proteomic profiles of HCC tumorigenic and non-tumorigenic cell lines. Using proteomic approaches in conjunction with the IPA database, we identified a number of putative proteins and signaling pathways associated with the initiation and progression of HCC. Further studies on their functional relevance to HCC development and progression will provide important insights into the mechanisms of HCV-induced hepatocarcinogenesis.

## MATERIALS AND METHODS

### Cells

Huh-7.4 and Huh-7.5 were cultured in DMEM/HIGH GLUCOSE (Thermo Scientific) supplemented with 10% FBS (GEMINI BIO PRODUCTS), penicillin-streptomycin (Thermo Scientific) and non-essential amino acids (Sigma-Aldrich) at 37°C and 5% CO_2_.

### Mice

Female FOXn1-nude mice (4 weeks of age) were purchased from the Jackson Laboratory (Bar Harbor, ME). Mice were housed in cages and were fed with food and water ad libitum, with a 12 h light and 12 h dark cycle.

### Human HCC xenograft tumor study

Five female nude mice at 6 weeks of age were used for a tumorigenicity study. Three million (3 × 10^6^) of Huh-7.4 and Huh-7.5 cells in PBS were injected subcutaneously (SC) into the flank area on the left and right sides of each mouse, respectively. Each mouse was observed weekly for tumor development. The animal care and study protocol was approved by University of Alabama at Birmingham (UAB) Institution Animal Care and Use Committee (IACUC).

### Immunoblotting

Huh-7.4 and Huh-7.5 Cells were lysed in a RIPA buffer containing protease inhibitor cocktail (Roche). After centrifugation, the supernatants were collected (cytosolic fraction) and protein concentration was measured using a protein assay reagent (Bio-Rad). A total of 25 μg of protein was loaded onto a 10% SDS-PAGE. Upon electrophoresis, proteins were transferred onto a PVDF membrane for immunoblotting. 1:500 dilutions of primary antibodies were used. All antibodies were purchased from Santa Cruz Biotechnology. Specific proteins were detected with Pierce ECL Western Blotting Substrate (Thermo Scientific) and imaged using a ChemiDoc MP Imaging System (BIO-RAD).

### Proteomic analysis

#### Sample preparation

Huh-7.4 and Huh-7.5 cells were cultured in a 10 cm dish to reach 70-80% confluency. Cells were washed with ice-cold PBS and were collected by scraping. After centrifugation, cell pellets were collected and subjected to proteomic profiling by UAB Comprehensive Cancer Center Mass Spectrometry/Proteomics (MSP) Core. The protein fractions were extracted from the pellet and concentrated, and the buffer was exchanged using 3kDa MW cut-off columns (Millipore). The sample was then diluted in LDS-PAGE buffer (Invitrogen) followed by reducing, denaturing, and separation on an SDS Bis-Tris short stack gel (4%, Invitrogen). The gel was stained overnight with colloidal blue (Invitrogen). The stained fraction was cut out and equilibrated in 100 mM ammonium bicarbonate (AmBc). Gel slices were reduced, carbamidomethylated, dehydrated, and digested with Trypsin Gold (Promega) as per manufacturer instructions. Following digestion, peptides were extracted, concentrated under vacuum, and solubilized in 0.1% formic acid before analysis by 1D reverse-phase LC-ESI-MS2, as outlined below.

#### Mass spectrometry

Peptide digests were injected onto a Surveyor HPLC Plus (Thermo Scientific) using a split-flow configuration on the back end of a 100-micron I.D. x 13 cm pulled tip C-18 column (Jupiter C-18 300 Å, 5 micron, Phenomenex). This system runs in-line with a Thermo Orbitrap Velos Pro hybrid mass spectrometer, equipped with a nano-electrospray source (Thermo Scientific), and all data were collected in CID mode. Peptide fractions were directly sprayed into the mass spectrometer over the course of a 90-minute gradient, set to increase from 0%-30% acetonitrile in deionized H_2_O containing 0.1% formic acid and with a flow rate of 0.3 μl/min. Following each parent ion scan, fragmentation data were collected on the 18 most intense ions. Before and following the analysis window, the spray voltage was set to 0.0 kV, and the flow rate was set at 3 μl/min. During data collection, the instrument was configured as follows: spray voltage 1.9 kV, capillary temperature 170°C, 1 microscan with a maximum inject time of 25 ms for all modes. The fragmentation scan was obtained at a 60 K resolution with a minimum signal threshold of 2000 counts. The activation settings were charge state 3, isolation width 2.0 m/z, normalized collision energy 30.0, activation Q 0.250, and activation time 25 ms. For the dependent scans, charge state screening was enabled, and the dynamic exclusion was enabled with the following settings: repeat count 2, repeat duration 15.0s, exclusion list size 500, and exclusion duration 60.0s.

#### MS data conversion and searches

The XCalibur RAW files were collected in profile mode, centroided and converted to MzXML using ReAdW v. 3.5.1. The mgf files were then created using MzXML2Search (included in TPP v. 3.5) for all scans with a precursor mass between 350Da and 2,000Da. The data were searched using SEQUEST, which was set for 3 maximum missed cleavages, a precursor mass window of 20ppm, trypsin digestion, variable modification C at 57.0293, and M at 15.9949. For the fragment-ion mass tolerance, 0.0Da was used. Searches were performed with a human subset of the UniRef100 database, which includes common contaminants such as digestion enzymes and human keratin, in addition to sequences specific to these experiments.

#### Filtering and system biology

A list of peptide IDs were generated based on SEQUEST search results, which were filtered using Scaffold (Protein Sciences). The scaffold was applied to filter and group all of the matching peptides to generate and retain only high-confidence IDs, while also generating normalized spectral counts (SC) across all samples for the purpose of relative quantification. The filter cutoff values were set with peptide length (>5 AA’s); no peptides with a MH+1 charge state were included. Peptide probabilities were calculated and set to >90% C.I., with the number of peptides per protein set at 2 or more, and protein probabilities set to >97% C.I.; all combined, this resulted in a list of protein IDs with >99% confidence. The scaffold incorporates the two most common methods for statistical validation of large proteome datasets, the false discovery rate and protein probability. Relative quantifications across experiments were performed via spectral counting, and spectral count abundances were then normalized between samples.

Statistical analysis was carried out between pair-wise groups using Significance Analysis of Microarray (SAM). Cut-off was set at ±3, and a fold change cut-off set at ±3, in addition to Wilcoxon rank sum test with p<0.05. The list of statistically significant protein hits was combined with protein IDs that were only observed within a single group, and were then further analyzed with the Ingenuity pathway analysis.

### Ingenuity pathway analysis (IPA)

Datasets of differentially expressed proteins were analyzed through the use of IPA core pathway analysis (QIAGEN Ingenuity Systems). Functional analysis of entire datasets identified the biological functions and diseases that were most significant to the dataset. Proteins from the dataset that were associated with biological functions or diseases in the Ingenuity Pathways Knowledge Base were considered for the analysis (IPA-mapped IDs). Canonical pathway analysis of entire datasets identified, from among the IPA library of canonical pathways, those that were most significant to the dataset. The identification was based upon proteins within the dataset that were associated with a canonical pathway in the Ingenuity Pathways Knowledge Base.

## SUPPLEMENTARY MATERIALS FIGURES AND TABLES



































## Abbreviations

HCC: hepatocellular carcinoma
HBV: hepatitis B virus
HCV: hepatitis C virus
mTOR: The mechanistic target of rapamycin
EIF: eukaryote translation initiation factors
EGF: epidermal growth factor
EGFR: epidermal growth factor receptor
NASH: nonalcoholic steatohepatitis
IPA: ingenuity pathway analysis
MAPK/ERK: mitogen-activated protein kinases/extracellular signal-regulated kinases
GNAQ: G protein subunit alpha Q
NFE2L2: nuclear factor, erythroid 2 like 2
XBP1: X-box binding protein 1
TGF-B: transforming growth factor beta
MKNK1: MAP kinase interacting serine/threonine kinase 1
INSR: insulin receptor
KRT8: Keratin 8
FN1: Fibronectin 1
GLS: glutaminase
ANX A3: annexin A3
ACSL3: acyl-coA synthetase 3
UCHL-1: Ubiquitin C-terminal hydrolase-L1
