# Supplementary material for: Identification of cellular genes and pathways important for tumorigenicity of hepatocellular carcinoma cell lines by proteomic profiling
Source: Oncotarget. 2017 Sep 27;8(56):96171–83. doi: 10.18632/oncotarget.21821 (PMC5707090; doi:10.18632/oncotarget.21821)
Supplement: Supplementary file 1 [file oncotarget-08-96171-s001.pdf]

Identification of cellular genes and pathways important for tumorigenicity of hepatocellular carcinoma cell lines by proteomic profiling

SUPPLEMENTARY MATERIALS

Supplementary File 1: Gene annotation, fold spectral-change, and the identifier of the original data.

See Supplementary File 1

Supplementary File 2: The datasets of proteins which were generated, mapped and analyzed by IPA in duplicate performed in the same manner for each cell line.

See Supplementary File 2

Supplementary File 3: Detailed high-resolution bar graphs and the classification of each of the proteins from Huh-7.4 and Huh-7.5.

See Supplementary File 3

Supplementary File 4: The ten proteins with the highest magnitude change (up or down) and the top pathways associated with these proteinsare are summarized.

See Supplementary File 4

Supplementary File 5: A list of potential drugs to use against Huh-7.4 cells.

See Supplementary File 5
