# Supplementary figures and images for: Identification of cellular genes and pathways important for tumorigenicity of hepatocellular carcinoma cell lines by proteomic profiling
Source: Oncotarget. 2017 Sep 27;8(56):96171–83. doi: 10.18632/oncotarget.21821 (PMC5707090; doi:10.18632/oncotarget.21821)

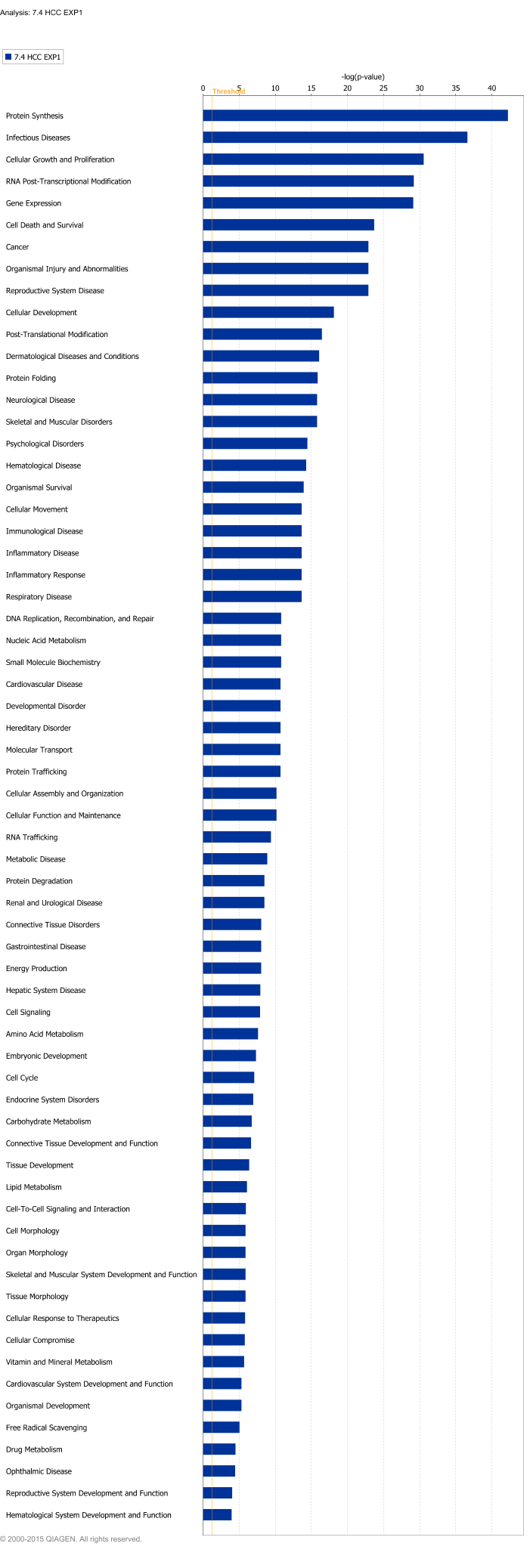

Supplement: Supplementary file 7 [file oncotarget-08-96171-s007.tif]

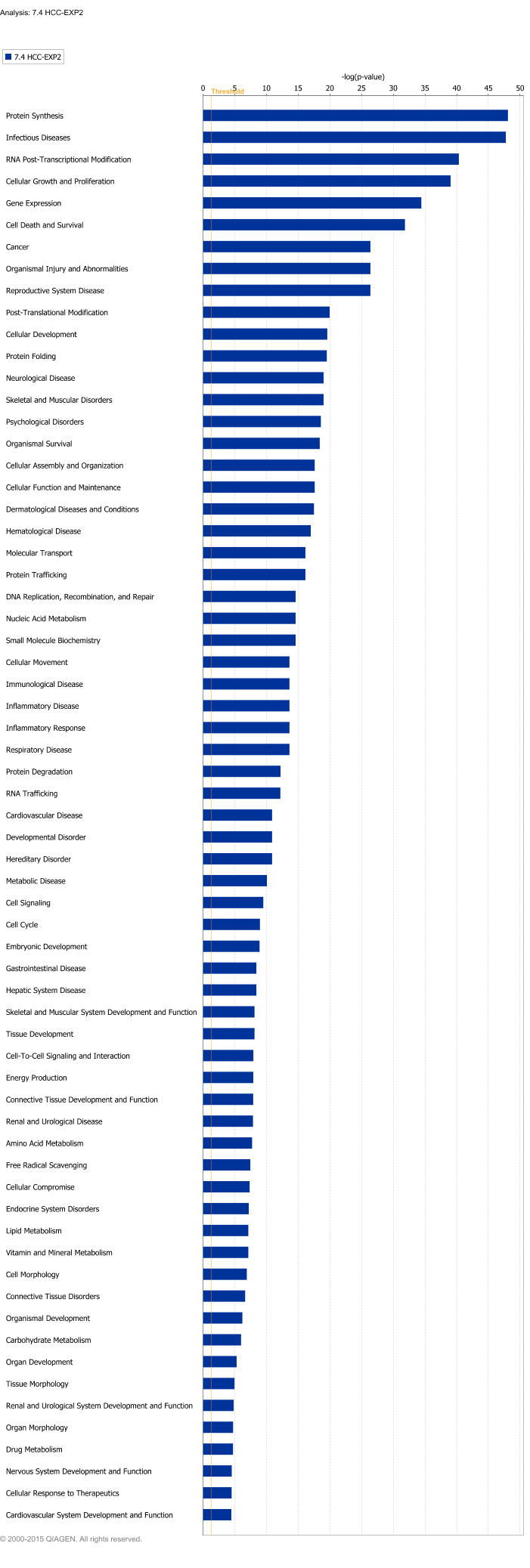

Supplement: Supplementary file 8 [file oncotarget-08-96171-s008.tif]

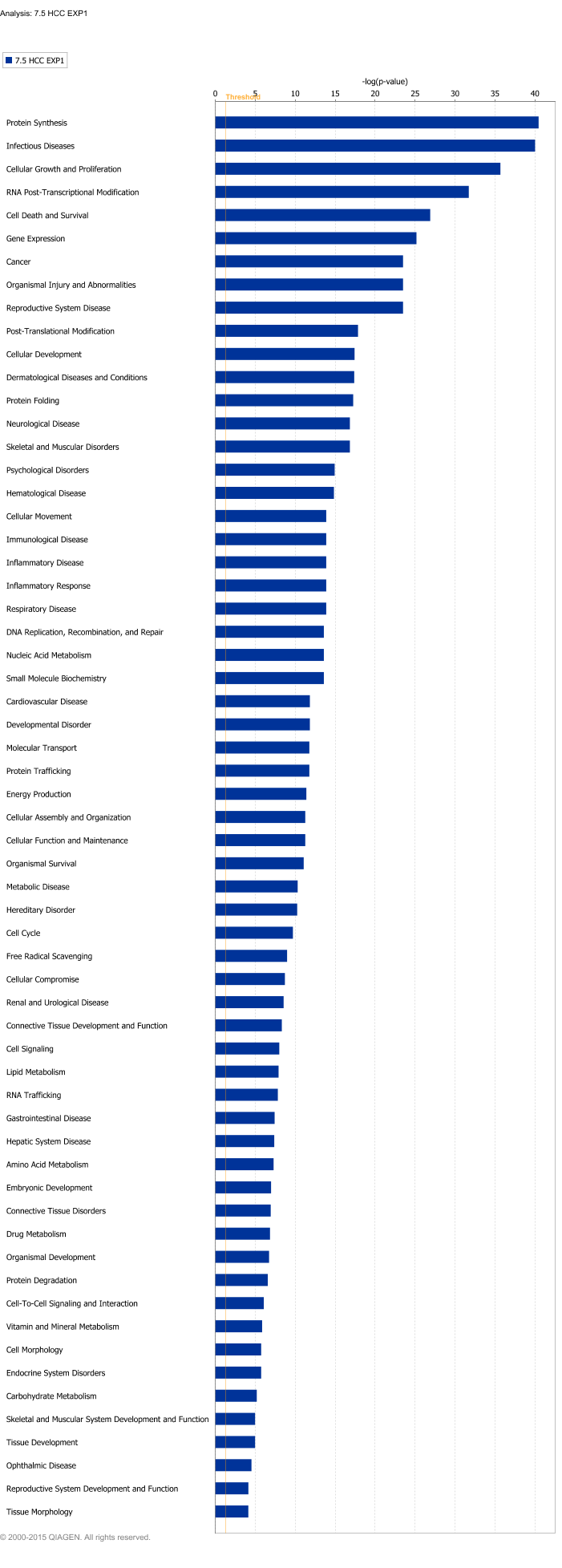

Supplement: Supplementary file 11 [file oncotarget-08-96171-s011.tif]

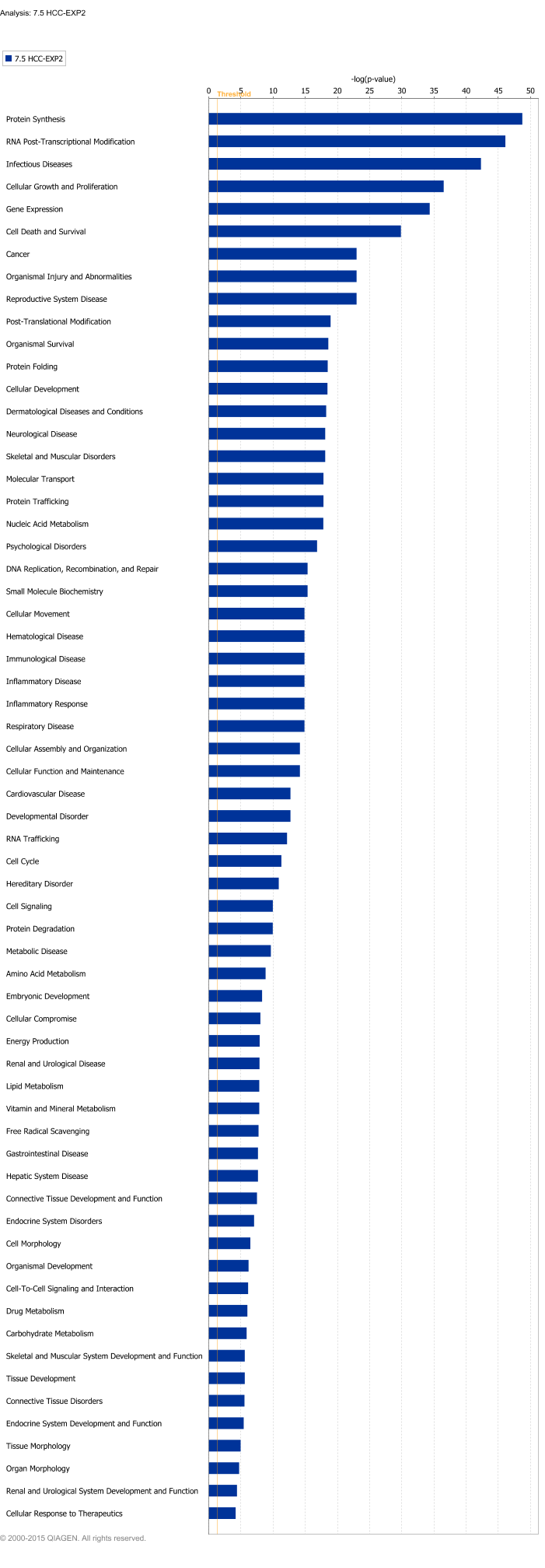

Supplement: Supplementary file 12 [file oncotarget-08-96171-s012.tif]

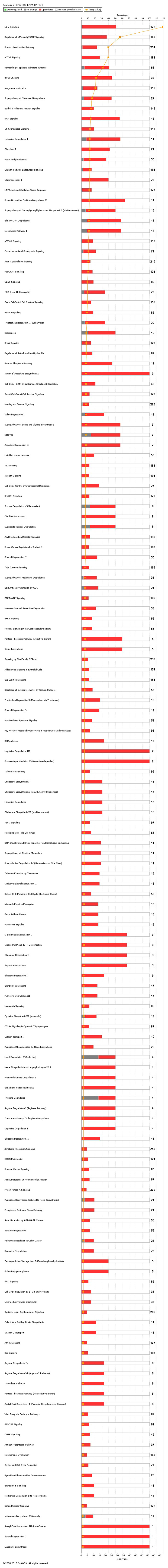

Supplement: Supplementary file 14 [file oncotarget-08-96171-s014.tif]
