# Supplementary material for: Identification of cellular genes and pathways important for tumorigenicity of hepatocellular carcinoma cell lines by proteomic profiling
Source: Oncotarget. 2017 Sep 27;8(56):96171–83. doi: 10.18632/oncotarget.21821 (PMC5707090; doi:10.18632/oncotarget.21821)
Supplement: Supplementary file 17 [file oncotarget-08-96171-s017.pdf]

INGENUITY
PATHWAY ANALYSIS

My Pathway

Report Date: 2015-11-20
Report Version: 355910
Content Version: 24718999 (Release Date: 2015-09-14)

My Pathway: New My Pathway 1

Notes: --

Top Functions & Diseases: Cancer; Organismal Injury and Abnormalities; Developmental Disorder

Molecules: ABCF1, ACBD3, ACIN1, ACSL3, AKR1C1/AKR1C2, AKR7A2, ALDH3A2, ANXA3, ANXA7, AP2M1, APMAP, APOB, ATP2B1, ATP6V1G1, ATXN2L, BAG6, BAX, BIN1, C11orf54, CCDC86, COPS4, COPZ1, COX5A, CRKL, CSRP2, CUL2, CYP51A1, DAD1, DAZAP1, DFNA5, DIAPH1, DIS3, ECH1, EDC4, EGFR, EHD4, EIF4E, EXOC4, FAM114A1, FBL, FKBP15, FLII, FLNC, FN1, FXR1, GAMT, GJA1, GLS, GNPDA1, GNS, GRPEL1, GSK3B, HEBP2, HIST1H2BK, HNRNPUL2, HSPBP1, IDH3A, IRF2BP2, ITGB1, KRT19, LMAN2, MAN2A1, MTHFD1L, MYH14, NAA50, NAGK, NDUFV2, NELFA, NLN, NUP155, NUP214, PARK7, PCYOX1, PCYT1A, PCYT2, PELO, PEPD, PGM2, PGM3, PLAA, POLR2A, PPP6C, PQBP1, PRDM15, PSMD4, QPRT, RAB18, RBMX, RCN1, RPL27A, RPS10, RPS15, RPS21, RRM2, RTCB, S100A10, SARS2, SCCPDH, SEPHS1, SERPINB1, SF1, SLIRP, SMC3, SMS, SPATS2, SPATS2L, SRP68, SRRT, STOM, STT3A, STX4, SUGT1, SYAP1, TAGLN, TCAF1, TMED9, TMEM2, TOR1AIP1, UNC45A, UTRN, VAMP3, XPOT, ZC3HAV1L
show preview

Drug Summary - Overview of drugs targeting molecules in My Pathway

Showing 134 of 134 row(s) of Drug data.

Drug Name	Targets	Actions	Brand Names	Indications/Status
(±)-2-hydroxyoleic acid	SMS	activator		glioma cancer/Phase 1/Phase 2
18F-PEG6-IPQA	EGFR	binder		EGFR amplification positive KRAS mutation negative non-small cell lung cancer/Phase 1<br>EGFR mutation positive KRAS mutation negative non-small cell lung cancer/Phase 1<br>KRAS mutation negative non-small cell lung cancer/Phase 1
5-fluorouracil/gemcitabine [gemcitabine]	RRM2	inhibitor		
ABT-414	EGFR	inhibitor		EGFR expressing glioblastoma/Phase 1<br>EGFR expressing glioblastoma/Phase 2<br>EGFR expressing non-small cell lung cancer/Phase 1<br>EGFR expressing solid tumor/Phase 1
AEE 788	EGFR	inhibitor		cancer/Phase 1<br>central nervous system tumor/Phase 1/Phase 2<br>glioblastoma/Phase 1/Phase 2
afatinib	EGFR	inhibitor	Gilotrif, Giotrif	adenocarcinoma/Phase 3<br>advanced non-small-cell lung cancer/Phase 2<br>advanced solid tumor/Phase 1<br>ALK fusion positive non-small cell lung cancer/Phase 1<br>anaplastic astrocytoma/Phase 1<br>anaplastic oligodendroglioma/Phase 1<br>bile duct carcinoma/Phase 1<br>brain cancer/Phase 1<br>brain metastasis/Phase 1<br>cancer/Phase 2<br>carcinomatous meningitis/Phase 1<br>colorectal tumor/Phase 1/Phase 2<br>colorectal tumor/Phase 2<br>diffuse intrinsic pontine glioma/Phase 1<br>EGFR (L858R) positive lung adenocarcinoma/Phase 4<br>EGFR activating mutation positive adenocarcinoma of the lung/Phase 2<br>EGFR exon 19 deletion positive EGFR L858R mutation positive non-small cell lung cancer/Phase 4<br>EGFR exon 19 deletion positive EGFR T790M mutation negative non-squamous non-small cell lung carcinoma/Phase 1<br>EGFR exon 19 deletion positive lung adenocarcinoma/Phase 4<br>EGFR exon 19 deletion positive non-small cell lung cancer/Phase 1<br>EGFR exon 19 deletion positive non-small cell lung cancer/Phase 1/Phase 2<br>EGFR exon 19 deletion positive non-small cell lung cancer/Phase 4<br>EGFR G718X mutation positive non-small cell lung cancer/Phase 1<br>EGFR G719X mutation positive EGFR T790M mutation negative non-squamous non-small cell lung carcinoma/Phase 1<br>EGFR L858R mutation positive EGFR T790M mutation negative non-squamous non-small cell carcinoma of lung/Phase 1<br>EGFR L858R mutation positive non-small cell lung cancer/Phase 1<br>EGFR L858R mutation positive

© 2000-2014 QIAGEN. All rights reserved.
INGENUITY SYSTEMS

non-small cell lung cancer/Phase 1/Phase 2
EGFR L858R mutation positive non-small cell lung cancer/Phase 4
EGFR L861Q mutation positive non-small cell lung cancer/Phase 1
EGFR L861X mutation positive EGFR T790M mutation negative non-squamous non-small cell lung carcinoma/Phase 1
EGFR mutation positive non small cell lung carcinoma/Phase 3
EGFR mutation positive non-small cell lung cancer/Phase 1
EGFR mutation positive non-small cell lung cancer/Phase 2
EGFR mutation positive non-small cell lung cancer/Phase 3
EGFR mutation positive non-small cell lung cancer/Unspecified phase
EGFR sensitizing mutation positive non-small cell lung cancer/Phase 2
EGFR T790M positive non-small cell lung cancer/Phase 1
EGFR T790M positive non-small cell lung carcinoma/Phase 1
EGFR(L858R) positive metastatic non-small cell lung cancer/Approved
ependymoma/Phase 1
esophageal squamous cell cancer/Phase 2
estrogen receptor positive HER2 negative breast adenocarcinoma/Phase 2
estrogen receptor positive HER2 negative breast cancer/Phase 2
gastric adenocarcinoma/Phase 2
gastric carcinoma/Phase 2
gastro-esophageal junction carcinoma/Phase 2
gastroesophageal junction adenocarcinoma/Phase 2
glioblastoma cancer/Phase 1
glioma cancer/Phase 1
glioma cancer/Phase 2
head and neck cancer/Phase 3
head and neck squamous cell carcinoma/Phase 1
head and neck squamous cell carcinoma/Phase 1/Phase 2
head and neck squamous cell carcinoma/Phase 2
head and neck squamous cell carcinoma/Phase 3
head and neck tumor/Phase 2
head and neck tumor/Phase 3
HER2 mutation positive non-small cell lung cancer/Phase 2
HER2 positive breast neoplasm/Phase 1
HER2 positive gastric adenocarcinoma/Phase 2
HER2 positive gastroesophageal cancer/Phase 2
HER2 positive gastroesophageal junction adenocarcinoma/Phase 2
HER2 positive intrathoracic esophageal adenocarcinoma/Phase 2
HER2 positive stomach neoplasm/Phase 1
HER2-positive breast cancer/Phase 2
hypopharyngeal squamous cell carcinoma/Phase 2
hypopharyngeal squamous cell carcinoma/Phase 3
KRAS wild-type metastatic colorectal cancer/Phase 1
KRAS wild-type metastatic colorectal cancer/Phase 2
laryngeal squamous cell carcinoma/Phase 2
laryngeal squamous cell carcinoma/Phase 3
laryngeal verrucous carcinoma/Phase 2
liver disease/Phase 1
locally advanced EGFR mutation positive non-small cell lung cancer/Phase 3
locally advanced EGFR mutation positive non-small cell lung carcinoma/Phase 3
low grade astrocytoma/Phase 1
low-grade glioma/Phase 1
lung cancer/Phase 2
lung tumor/Phase 2
malignant glioma/Phase 2
malignant solid tumor/Phase 1
malignant solid tumor/Phase 2
mammary tumor/Phase 1
mammary tumor/Phase 2
mammary tumor/Phase 3
medulloblastoma/Phase 1
meningioma/Phase 1

© 2000-2014 QIAGEN. All rights reserved.
INGENUITY® SYSTEMS

				metastasis/Phase 2<br>metastatic breast carcinoma/Phase 2<br>metastatic esophageal squamous cell carcinoma/Phase 2<br>metastatic estrogen receptor positive HER2 negative breast cancer/Phase 2<br>metastatic non-small-cell lung cancer/Phase 1<br>metastatic non-small-cell lung cancer/Phase 2/Phase 3<br>metastatic pancreatic adenocarcinoma/Phase 2<br>neuroblastoma/Phase 1<br>non small cell lung adenocarcinoma/Phase 2<br>non-small cell lung cancer with activating EGFR mutations/Phase 1<br>non-small cell lung cancer/Phase 1<br>non-small cell lung cancer/Phase 1/Phase 2<br>non-small cell lung cancer/Phase 2<br>non-small cell lung cancer/Phase 2/Phase 3<br>non-small cell lung cancer/Phase 3<br>non-small cell lung cancer/Unspecified phase<br>non-small-cell lung carcinoma/Approved<br>oropharyngeal squamous-cell carcinoma/Phase 3<br>pancreatic cancer/Phase 1<br>pancreatic carcinoma/Phase 1<br>pancreatic neoplasia/Phase 1/Phase 2<br>primary HER2 mutation positive non-small cell lung cancer/Phase 2<br>prostatic tumor/Phase 2<br>recurrent EGFR exon 19 deletion positive non-small cell lung cancer/Phase 1<br>recurrent EGFR L858R mutation positive non-small cell lung cancer/Phase 1<br>recurrent laryngeal squamous cell carcinoma/Phase 3<br>rhabdomyosarcoma/Phase 1<br>salivary gland cancer/Phase 2<br>squamous cell carcinoma of the oral cavity/Phase 3<br>squamous cell carcinoma/Phase 2<br>squamous cell carcinoma/Phase 3<br>squamous cell lung cancer/Phase 1<br>squamous cell lung cancer/Phase 2<br>stage III head and neck squamous cell carcinoma/Phase 3<br>stage III oropharyngeal squamous cell carcinoma/Phase 2<br>stage IVA oropharyngeal squamous cell carcinoma/Phase 2<br>stage IVB oropharyngeal squamous cell carcinoma/Phase 2<br>stage IVC oropharyngeal squamous cell carcinoma/Phase 2<br>tongue cancer/Phase 2<br>tumor/Phase 1<br>tumor/Phase 2<br>unilateral HER2 positive breast cancer/Phase 2<br>upper aero-digestive squamous cell carcinoma/Phase 1<br>urothelial cancer/Phase 2
alemtuzumab/cyclophosphamide/fludarabine phosphate/mitoxantrone [fludarabine phosphate]	RRM2	inhibitor		
alemtuzumab/cyclophosphamide/fludarabine phosphate/rituximab [fludarabine phosphate]	RRM2	inhibitor		
alemtuzumab/fludarabine phosphate [fludarabine phosphate]	RRM2	inhibitor		
anti-EGFR monoclonal antibody GT-MAB 5.2-GEX	EGFR	binder	CetuGEX	EGFR expressing head and neck squamous cell carcinoma/Phase 2<br>EGFR positive head and neck cancer/Phase 2<br>solid tumor/Phase 1
AP26113	EGFR	inhibitor		ALK fusion positive anaplastic large cell lymphoma/Phase 1/Phase 2<br>ALK fusion positive inflammatory myofibroblastic tumor/Phase 1/Phase 2<br>ALK fusion positive non-small cell lung cancer/Phase 1/Phase 2<br>ALK positive large B cell lymphoma/Phase 1/Phase 2<br>EGFR T790M positive non-small cell lung cancer/Phase 1/Phase 2<br>non-small cell lung cancer/Phase 1/Phase 2<br>progressive ALK fusion positive non-small cell lung cancer/Phase 2

© 2000-2014 QIAGEN. All rights reserved.
INGENUITY® SYSTEMS

Drug Name	Targets	Actions	Brand Names	Indications/Status
ASP8273	EGFR	inhibitor		EGFR exon 19 deletion positive non-small cell lung cancer/Phase 1<br>EGFR exon 19 deletion positive non-small cell lung cancer/Phase 1/Phase 2<br>EGFR exon 20 insertion positive non small cell lung cancer/Phase 1<br>EGFR G719X mutation positive non-small cell lung cancer/Phase 1<br>EGFR G719X mutation positive non-small cell lung cancer/Phase 1/Phase 2<br>EGFR L858R mutation positive non-small cell lung cancer/Phase 1<br>EGFR L858R mutation positive non-small cell lung cancer/Phase 1/Phase 2<br>EGFR L861Q mutation positive non-small cell lung cancer/Phase 1<br>EGFR L861Q mutation positive non-small cell lung cancer/Phase 1/Phase 2<br>non-small cell lung cancer with activating EGFR mutations/Phase 1<br>progressive EGFR T790M mutation positive non-small cell lung cancer/Phase 1/Phase 2
atorvastatin/niacin [nicotinic acid]	QPRT	binder		carotid atherosclerosis/Phase 2
avitinib	EGFR	inhibitor		EGFR T790M positive non-small cell lung cancer/Phase 1<br>non-small cell lung cancer with activating EGFR mutations/Phase 1/Phase 2
AZD3759	EGFR	inhibitor		
AZD9291	EGFR	inhibitor		advanced non-small-cell lung cancer/Phase 1/Phase 2<br>EGFR (L858R) positive lung adenocarcinoma/Phase 3<br>EGFR exon 19 deletion positive lung adenocarcinoma/Phase 3<br>EGFR exon 19 deletion positive non-small cell lung cancer/Phase 3<br>EGFR L858R mutation positive non-small cell lung cancer/Phase 3<br>EGFR mutation positive non-small cell lung cancer/Phase 1/Phase 2<br>EGFR sensitizing mutation positive EGFR T790M mutation positive non small cell lung cancer/Phase 1<br>EGFR sensitizing mutation positive non-small cell lung cancer/Phase 1<br>lung cancer/Phase 3<br>non-small cell lung cancer/Phase 2<br>progressive EGFR exon 19 deletion positive EGFR T790M mutation positive non small cell lung cancer/Phase 2<br>progressive EGFR G719X mutation positive EGFR T790M mutation positive non small cell lung cancer/Phase 2<br>progressive EGFR L858R mutation positive EGFR T790M mutation positive non small cell lung cancer/Phase 2<br>progressive EGFR L861Q mutation positive EGFR T790M mutation positive non small cell lung cancer/Phase 2<br>progressive EGFR sensitizing mutation positive EGFR T790M mutation positive non small cell lung cancer/Phase 2<br>progressive EGFR sensitizing mutation positive non-small cell lung cancer/Phase 1<br>progressive EGFR T790M mutation positive non-small cell lung cancer/Phase 3
betamethasone/clotrimazole [clotrimazole]	CYP51A1	antagonist	Betamethasone-Clotimazole	tinea corporis/Approved<br>tinea cruris/Approved<br>tinea pedis/Approved
bevacizumab/cetuximab [cetuximab]	EGFR	inhibitor		
bevacizumab/erlotinib [erlotinib]	EGFR	inhibitor		
bevacizumab/gemcitabine [gemcitabine]	RRM2	inhibitor		fallopian tube neoplasm/Phase 2<br>ovarian cancer/Phase 2
bevacizumab/panitumumab [panitumumab]	EGFR	antibody		
bifonazole	CYP51A1	inhibitor	Amycor, Azolmen, Mycospor	onychomycosis/Phase 3<br>tinea pedis/Phase 2
BMS-599626	EGFR	inhibitor		cancer/Phase 1<br>metastasis/Phase 1
bortezomib/cladribine/rituximab [cladribine]	RRM2	inhibitor		
cabozantinib/erlotinib [erlotinib]	EGFR	inhibitor		
cabozantinib/erlotinib [erlotinib]	EGFR	antagonist		
canertinib	EGFR	inhibitor		lung tumor/Phase 2<br>mammary tumor/Phase 2<br>non-small cell lung cancer/Phase 1
capecitabine/docetaxel/gemcitabine [gemcitabine]	RRM2	inhibitor		
capecitabine/erlotinib [erlotinib]	EGFR	antagonist		
capecitabine/gemcitabine [gemcitabine]	RRM2	inhibitor		

© 2000-2014 QIAGEN. All rights reserved.
INGENUITY® SYSTEMS

Drug Name	Targets	Actions	Brand Names	Indications/Status
capecitabine/lapatinib [lapatinib]	EGFR	antagonist	Tykerb/capecitabine, Tyverb/capecitabine	HER2 positive advanced breast cancer/Approved<br>HER2 positive metastatic breast cancer/Approved
CB-839	GLS	inhibitor		acute lymphocytic leukemia/Phase 1<br>acute myeloid leukemia/Phase 1<br>diffuse large B-cell lymphoma/Phase 1<br>HER2 negative hormone receptor negative breast cancer/Phase 1<br>IDH1 mutation positive tumor/Phase 1<br>IDH2 mutation positive tumor/Phase 1<br>mesothelioma/Phase 1<br>metastatic solid tumor/Phase 1<br>multiple myeloma/Phase 1<br>non small cell lung adenocarcinoma/Phase 1<br>non-Hodgkin's disease/Phase 1<br>non-small cell lung cancer/Phase 1<br>renal parenchyma cancer/Phase 1<br>renal-cell carcinoma/Phase 1<br>solid tumor/Phase 1<br>succinate dehydrogenase deficient gastrointestinal stromal tumor/Phase 1<br>Waldenstrom's macroglobulinemia/Phase 1
cetuximab	EGFR	antibody	Erbitux	adenocarcinoma/Phase 2<br>adult solid tumor/Phase 1<br>adult solid tumor/Phase 1/Phase 2<br>advanced cancer/Phase 1<br>advanced cancer/Phase 2<br>advanced colorectal cancer/Phase 2<br>advanced non-small-cell lung cancer/Phase 2<br>advanced solid tumor/Phase 1<br>anal cancer/Phase 1<br>anal cancer/Phase 2<br>anaplastic astrocytoma/Phase 1<br>appendix carcinoma/Phase 1<br>biliary tract cancer/Phase 2<br>bladder cancer/Phase 2<br>BRAF mutation negative colon adenocarcinoma/Phase 2<br>BRAF mutation negative KRAS mutation negative colon adenocarcinoma/Phase 2<br>BRAF mutation negative KRAS mutation negative colorectal cancer/Phase 2<br>BRAF mutation negative KRAS mutation negative NRAS mutation negative colon adenocarcinoma/Phase 2<br>BRAF mutation negative KRAS mutation negative NRAS mutation negative colorectal adenocarcinoma/Phase 2<br>BRAF mutation negative KRAS mutation negative NRAS mutation negative colorectal cancer/Phase 1/Phase 2<br>BRAF mutation negative KRAS mutation negative NRAS mutation negative colorectal cancer/Phase 2<br>BRAF mutation negative KRAS mutation negative NRAS mutation negative PIK3CA mutation negative colorectal cancer/Phase 1/Phase 2<br>BRAF mutation negative KRAS mutation negative NRAS mutation negative rectal adenocarcinoma/Phase 2<br>BRAF mutation negative KRAS mutation negative rectal adenocarcinoma/Phase 2<br>BRAF mutation negative rectal adenocarcinoma/Phase 2<br>BRAF mutation positive colorectal cancer/Phase 1/Phase 2<br>BRAF mutation positive colorectal cancer/Phase 2<br>BRAF V600 mutation positive cancer/Phase 2<br>BRAF V600 mutation positive KRAS mutation negative cancer/Phase 1<br>BRAF V600 mutation positive KRAS mutation negative malignant neoplasm/Phase 1<br>BRAF V600 mutation positive KRAS mutation negative RNF43 mutation positive colorectal cancer/Phase 1/Phase 2<br>BRAF V600 mutation positive KRAS mutation negative RNF43 mutation positive RSPO fusion positive colorectal cancer/Phase 1/Phase 2<br>BRAF V600 mutation positive

© 2000-2014 QIAGEN. All rights reserved.

KRAS mutation negative RSPO fusion positive colorectal cancer/Phase 1/Phase 2
BRAF V600 mutation positive KRAS mutation negative solid tumor/Phase 1
BRAF V600 mutation positive multiple myeloma/Phase 2
BRAF V600 mutation positive solid tumor/Phase 2
BRAF V600E positive KRAS mutation negative colorectal cancer/Phase 1
breast cancer/Phase 1
breast cancer/Phase 2
c-MET amplified solid tumor/Phase 1
C-MET positive head and neck squamous cell carcinoma/Phase 1/Phase 2
c-MET positive KRAS mutation negative NRAS mutation negative colorectal cancer/Phase 1/Phase 2
cancer/Phase 1
cancer/Phase 2
carcinoma/Phase 2
Castleman's disease/Phase 1
CDKN2A mutation negative oropharyngeal carcinoma/Phase 2
CDKN2A negative oropharyngeal squamous cell carcinoma/Phase 2
CDKN2A positive oropharyngeal carcinoma/Phase 2
CDKN2A positive oropharyngeal squamous cell carcinoma/Phase 2
CDKN2A positive oropharyngeal squamous cell carcinoma/Phase 3
cervical adenocarcinoma/Phase 1
cervical adenosquamous carcinoma/Phase 1
cervical cancer/Phase 1
cervical cancer/Phase 2
cervical small cell carcinoma/Phase 1
cervical squamous cell carcinoma/Phase 1
cervical squamous cell carcinoma/Phase 2
cholangiocarcinoma/Phase 2
colon adenocarcinoma/Phase 1
colon adenocarcinoma/Phase 1/Phase 2
colon adenocarcinoma/Phase 3
colon cancer/Phase 0
colon cancer/Phase 1
colon cancer/Phase 1/Phase 2
colon cancer/Phase 2
colon cancer/Phase 3
colon carcinoma/Phase 1
colon tumor/Phase 1
colorectal adenocarcinoma/Phase 1
colorectal cancer/Phase 1
colorectal cancer/Phase 1/Phase 2
colorectal cancer/Phase 2
colorectal cancer/Phase 2/Phase 3
colorectal cancer/Phase 3
colorectal cancer/Phase 4
colorectal cancer/Unspecified phase
colorectal carcinoma/Phase 1
colorectal carcinoma/Phase 1/Phase 2
colorectal carcinoma/Phase 2
colorectal tumor/Phase 1
colorectal tumor/Phase 2
dermatological disorder/Phase 2
EGFR expressing colorectal cancer/Phase 2
EGFR expressing epithelioid malignant pleural mesothelioma/Phase 2
EGFR expressing head and neck squamous cell carcinoma/Phase 2
EGFR expressing KRAS mutation negative colorectal adenocarcinoma/Phase 4
EGFR mutation positive esophageal squamous cell carcinoma/Phase 2
EGFR positive head and neck cancer/Phase 2
endometrial cancer/Phase 2
Erdheim-Chester disease/Phase 1
esophageal adenocarcinoma/Phase 2
esophageal adenocarcinoma/Unspecified phase
esophageal cancer/Phase 1
esophageal cancer/Phase 1/Phase 2
esophageal cancer/Phase 2
esophageal cancer/Phase 2/Phase 3
esophageal cancer/Phase 3
esophageal carcinoma/Phase 2

© 2000-2014 QIAGEN. All rights reserved.

esophageal squamous cell cancer/Phase 2
esophageal squamous cell carcinoma/Phase 2
esophagogastric junction cancer/Phase 2
extrahepatic bile duct cancer/Phase 1
extrahepatic cholangiocarcinoma/Phase 2
fallopian tube neoplasm/Phase 2
gall bladder adenocarcinoma/Phase 2
gallbladder cancer/Phase 1
gastric adenocarcinoma/Phase 2
gastric cancer/Phase 1
gastric cancer/Phase 1/Phase 2
gastric cancer/Phase 2
gastric cancer/Phase 3
gastric cancer/Unspecified phase
gastrointestinal stromal tumor/Phase 1
glioblastoma cancer/Phase 2
glioblastoma/Phase 1
head and neck adenoid cystic carcinoma/Phase 1/Phase 2
head and neck cancer/Phase 0
head and neck cancer/Phase 1
head and neck cancer/Phase 1/Phase 2
head and neck cancer/Phase 2
head and neck cancer/Phase 3
head and neck cancer/Phase 4
head and neck cancer/Unspecified phase
head and neck carcinoma/Phase 2
head and neck squamous cell cancer/Phase 1
head and neck squamous cell cancer/Phase 2
head and neck squamous cell cancer/Phase 3
head and neck squamous cell cancer/Unspecified phase
head and neck squamous cell carcinoma/Phase 1
head and neck squamous cell carcinoma/Phase 1/Phase 2
head and neck squamous cell carcinoma/Phase 2
head and neck squamous cell carcinoma/Phase 3
head and neck tumor/Phase 1
head and neck tumor/Phase 1/Phase 2
head and neck tumor/Phase 2
hepatocellular carcinoma/Phase 2
HER2 negative hormone receptor negative breast cancer/Phase 1
HER2 negative hormone receptor negative breast cancer/Phase 2
HER2 positive EGFR positive breast cancer/Phase 1
HER2 positive esophagogastric junction cancer/Phase 1/Phase 2
HER2 positive gastric cancer/Phase 1/Phase 2
HER2 positive metastatic breast cancer/Phase 1/Phase 2
HER3 positive carcinoma/Phase 1
hormone refractory prostate cancer/Phase 2
hypopharyngeal cancer/Phase 2
hypopharyngeal squamous cell carcinoma/Phase 1
hypopharyngeal squamous cell carcinoma/Phase 1/Phase 2
hypopharyngeal squamous cell carcinoma/Phase 2
hypopharyngeal squamous cell carcinoma/Phase 2/Phase 3
hypopharyngeal squamous cell carcinoma/Phase 3
hypopharyngeal squamous cell carcinoma/Phase 4
hypopharyngeal squamous cell carcinoma/Unspecified phase
infection/Phase 2
KRAS codon 12 mutation positive colorectal cancer/Phase 2
KRAS codon 13 mutation positive colorectal cancer/Phase 2
KRAS codon 61 mutation positive colorectal cancer/Phase 2
KRAS mutation negative adenocarcinoma of the colon/Phase 1
KRAS mutation negative adenocarcinoma of the colon/Phase 2
KRAS mutation negative adenocarcinoma of the rectum/Phase 1
KRAS mutation negative adenocarcinoma of the rectum/Phase 2
KRAS mutation negative and

© 2000-2014 QIAGEN. All rights reserved.

NRAS mutation negative colorectal cancer/Phase 1/Phase 2
KRAS mutation negative and NRAS mutation negative colorectal cancer/Phase 2
KRAS mutation negative and NRAS mutation negative colorectal cancer/Phase 3
KRAS mutation negative colon carcinoma/Phase 3
KRAS mutation negative colorectal adenocarcinoma/Phase 2
KRAS mutation negative head and neck squamous cell carcinoma/Phase 1
KRAS mutation negative NRAS mutation negative colon adenocarcinoma/Phase 2
KRAS mutation negative NRAS mutation negative colon adenocarcinoma/Phase 3
KRAS mutation negative NRAS mutation negative colorectal adenocarcinoma/Phase 1/Phase 2
KRAS mutation negative NRAS mutation negative rectal adenocarcinoma/Phase 2
KRAS mutation negative NRAS mutation negative rectal adenocarcinoma/Phase 3
KRAS mutation negative penis cancer/Phase 2
KRAS mutation negative penis squamous cell carcinoma/Phase 2
KRAS mutation negative rectal cancer/Phase 2
KRAS mutation negative rectal carcinoma/Phase 3
KRAS mutation positive colorectal cancer/Phase 2
KRAS wild-type liver metastasis associated with KRAS wild-type metastatic colorectal cancer/Phase 2
KRAS wild-type liver metastasis/Phase 2
KRAS wild-type metastatic colorectal cancer/Phase 1
KRAS wild-type metastatic colorectal cancer/Phase 2
KRAS wild-type positive colorectal cancer/Phase 1
KRAS wild-type positive colorectal cancer/Phase 2
large cell lung cancer/Phase 2
laryngeal cancer/Phase 1
laryngeal cancer/Phase 2
laryngeal carcinoma/Phase 2
laryngeal squamous cell carcinoma/Phase 1
laryngeal squamous cell carcinoma/Phase 1/Phase 2
laryngeal squamous cell carcinoma/Phase 2
laryngeal squamous cell carcinoma/Phase 2/Phase 3
laryngeal squamous cell carcinoma/Phase 3
laryngeal squamous cell carcinoma/Phase 4
laryngeal squamous cell carcinoma/Unspecified phase
laryngeal tumor/Phase 2
laryngeal verrucous carcinoma/Phase 1
laryngeal verrucous carcinoma/Phase 1/Phase 2
laryngeal verrucous carcinoma/Phase 2
laryngeal verrucous carcinoma/Phase 2/Phase 3
laryngeal verrucous carcinoma/Unspecified phase
liver cancer/Phase 1
liver cancer/Phase 2
liver lesion associated with metastatic colorectal cancer/Phase 2
liver metastasis associated with EGFR-expressing KRAS mutation-negative metastatic colorectal cancer/Phase 2
liver metastasis associated with KRAS wild-type metastatic colorectal cancer/Phase 2
liver metastasis associated with metastatic colorectal cancer/Phase 2
liver metastasis associated with metastatic KRAS-mutation-positive colorectal cancer/Phase 2
liver metastasis/Phase 0
liver metastasis/Phase 2
liver metastasis/Unspecified phase
loco-regionally advanced squamous cell carcinoma of the head and neck/Approved

© 2000-2014 QIAGEN. All rights reserved.

lung adenocarcinoma/Phase 1/Phase 2
lung adenosquamous cancer/Phase 2
lung cancer/Phase 1
lung cancer/Phase 1/Phase 2
lung cancer/Phase 2
lung cancer/Phase 3
lung tumor/Phase 2
lymphangioleiomyomatosis/Phase 1
malignant glioma/Phase 2
malignant pleural effusion/Phase 2
malignant solid tumor/Phase 1
malignant tumor/Phase 3
mammary tumor/Phase 2
metastasis/Phase 1
metastasis/Phase 1/Phase 2
metastasis/Phase 2
metastasis/Phase 3
metastasis/Phase 4
metastatic breast cancer/Phase 2
metastatic colorectal adenocarcinoma/Phase 1/Phase 2
metastatic colorectal cancer/Phase 1
metastatic colorectal cancer/Phase 1/Phase 2
metastatic colorectal cancer/Phase 2
metastatic colorectal cancer/Phase 3
metastatic colorectal cancer/Phase 4
metastatic colorectal cancer/Unspecified phase
metastatic colorectal carcinoma/Phase 1/Phase 2
metastatic head and neck squamous cell carcinoma/Phase 1
metastatic head and neck squamous cell carcinoma/Phase 1/Phase 2
metastatic head and neck squamous cell carcinoma/Phase 2
metastatic KRAS mutation negative colorectal adenocarcinoma/Phase 2
metastatic malignant colorectal neoplasm/Approved
metastatic non-small-cell lung cancer/Phase 1
metastatic non-small-cell lung cancer/Phase 1/Phase 2
metastatic non-small-cell lung cancer/Phase 2
metastatic non-small-cell lung cancer/Phase 2/Phase 3
metastatic non-small-cell lung cancer/Phase 3
metastatic pancreatic cancer/Phase 1
metastatic pancreatic cancer/Phase 1/Phase 2
metastatic pancreatic cancer/Phase 2
metastatic solid tumor/Phase 1
metastatic solid tumor/Phase 1/Phase 2
metastatic squamous cell carcinoma of the hypopharynx/Phase 1
metastatic squamous cell carcinoma of the hypopharynx/Phase 1/Phase 2
metastatic squamous cell carcinoma of the hypopharynx/Phase 2
metastatic squamous cell carcinoma of the larynx/Phase 1
metastatic squamous cell carcinoma of the larynx/Phase 1/Phase 2
metastatic squamous cell carcinoma of the larynx/Phase 2
metastatic squamous cell carcinoma of the oral cavity/Phase 1
metastatic squamous cell carcinoma of the oral cavity/Phase 1/Phase 2
metastatic squamous cell carcinoma of the oral cavity/Phase 2
mucinous adenocarcinoma of colon/Phase 0
mucinous adenocarcinoma of colon/Phase 1
mucinous adenocarcinoma of colon/Phase 2
mucinous adenocarcinoma of rectum/Phase 0
mucinous adenocarcinoma of rectum/Phase 1
multiple myeloma/Phase 2
nasopharyngeal carcinoma/Phase 1/Phase 2

© 2000-2014 QIAGEN. All rights reserved.

nasopharyngeal carcinoma/Phase 2
neoplasia/Phase 2
neuralgia/Phase 2
non small cell lung adenocarcinoma/Phase 2
non-small cell lung cancer/Phase 1
non-small cell lung cancer/Phase 1/Phase 2
non-small cell lung cancer/Phase 2
non-small cell lung cancer/Phase 2/Phase 3
non-small cell lung cancer/Phase 3
non-squamous non-small cell lung cancer/Phase 2
oral cancer/Phase 1
oral squamous cell carcinoma/Phase 1
oral squamous cell carcinoma/Phase 2
oropharyngeal cancer/Phase 2
oropharyngeal squamous-cell carcinoma/Phase 1
oropharyngeal squamous-cell carcinoma/Phase 1/Phase 2
oropharyngeal squamous-cell carcinoma/Phase 2
oropharyngeal squamous-cell carcinoma/Phase 3
oropharyngeal squamous-cell carcinoma/Phase 4
ovarian cancer/Phase 2
pancreatic adenocarcinoma/Phase 2
pancreatic cancer/Phase 1
pancreatic cancer/Phase 1/Phase 2
pancreatic cancer/Phase 2
pancreatic cancer/Phase 3
pancreatic cancer/Unspecified phase
peritoneal cavity cancer/Phase 2
peritoneal tumor/Phase 2
pharyngeal cancer/Phase 1
pharyngeal tumor/Phase 2
precancerous condition/Phase 2
precancerous condition/Unspecified phase
primary glioblastoma/Phase 1/Phase 2
primary head and neck squamous cell carcinoma/Phase 1
primary laryngeal squamous cell carcinoma/Phase 1
primary oral squamous cell carcinoma/Phase 1
primary peritoneal cancer/Phase 2
prostate cancer/Phase 2
prostatic tumor/Phase 2
radiation toxicity/Phase 3
RAS mutation negative colon adenocarcinoma/Phase 2
RAS mutation negative colorectal adenocarcinoma/Phase 2
RAS mutation negative colorectal cancer/Phase 2
RAS mutation negative rectal adenocarcinoma/Phase 2
rectal adenocarcinoma/Phase 1
rectal adenocarcinoma/Phase 1/Phase 2
rectal adenocarcinoma/Phase 2
rectum cancer/Phase 0
rectum cancer/Phase 1
rectum cancer/Phase 1/Phase 2
rectum cancer/Phase 2
rectum cancer/Phase 3
rectum carcinoma/Phase 1
recurrent cancer/Phase 1
recurrent cervical carcinoma/Phase 2
recurrent laryngeal squamous cell carcinoma/Phase 1
recurrent laryngeal squamous cell carcinoma/Phase 1/Phase 2
recurrent laryngeal squamous cell carcinoma/Phase 2
recurrent loco-regionally advanced squamous cell carcinoma of the head and neck/Approved
recurrent oropharyngeal squamous cell carcinoma/Phase 1
recurrent oropharyngeal squamous cell carcinoma/Phase 1/Phase 2
recurrent oropharyngeal squamous cell carcinoma/Phase 2
recurrent rectal cancer/Phase 0
recurrent rectal cancer/Phase 1
recurrent rectal cancer/Phase 1/Phase 2
recurrent rectal cancer/Phase 2
recurrent rectal cancer/Phase 3
renal cancer/Phase 1
renal parenchyma cancer/Phase 1/Phase 2
salivary gland cancer/Phase 1
salivary gland cancer/Phase

© 2000-2014 QIAGEN. All rights reserved.

1/Phase 2
salivary gland cancer/Phase 2
salivary gland carcinoma/Phase 1
salivary gland carcinoma/Phase 1/Phase 2
salivary gland carcinoma/Phase 2
sarcoma/Phase 1
sarcoma/Phase 2
signet ring adenocarcinoma of the colon/Phase 0
signet ring adenocarcinoma of the colon/Phase 1
signet ring adenocarcinoma of the colon/Phase 2
signet ring adenocarcinoma of the rectum/Phase 0
signet ring adenocarcinoma of the rectum/Phase 1
skin cancer/Unspecified phase
skin squamous cell carcinoma/Unspecified phase
small intestine cancer/Phase 1
solid tumor/Phase 1
solid tumor/Phase 1/Phase 2
squamous cell carcinoma of nasopharynx/Phase 1
squamous cell carcinoma of nasopharynx/Phase 1/Phase 2
squamous cell carcinoma of nasopharynx/Phase 2
squamous cell carcinoma of the oral cavity/Phase 1/Phase 2
squamous cell carcinoma of the oral cavity/Phase 2
squamous cell carcinoma of the oral cavity/Phase 3
squamous cell carcinoma of the oral cavity/Phase 4
squamous cell carcinoma/Phase 1
squamous cell carcinoma/Phase 1/Phase 2
squamous cell carcinoma/Phase 2
squamous cell lung cancer/Phase 1
squamous cell lung cancer/Phase 2
stage IB2 cervical carcinoma/Phase 2
stage III head and neck squamous cell carcinoma/Phase 3
stage III oropharyngeal squamous cell carcinoma/Phase 1
stage III oropharyngeal squamous cell carcinoma/Phase 1/Phase 2
stage III oropharyngeal squamous cell carcinoma/Phase 2
stage III oropharyngeal squamous cell carcinoma/Phase 2/Phase 3
stage III oropharyngeal squamous cell carcinoma/Phase 3
stage III oropharyngeal squamous cell carcinoma/Phase 4
stage III oropharyngeal squamous cell carcinoma/Unspecified phase
stage IV gastric cancer/Phase 1
stage IV head and neck squamous cell carcinoma/Phase 2
stage IV head and neck squamous cell carcinoma/Phase 3
stage IV oropharyngeal squamous cell carcinoma/Phase 1
stage IV oropharyngeal squamous cell carcinoma/Phase 1/Phase 2
stage IV oropharyngeal squamous cell carcinoma/Phase 2
stage IV oropharyngeal squamous cell carcinoma/Phase 4
stage IVA oropharyngeal squamous cell carcinoma/Phase 1
stage IVA oropharyngeal squamous cell carcinoma/Phase 1/Phase 2
stage IVA oropharyngeal squamous cell carcinoma/Phase 2
stage IVA oropharyngeal squamous cell carcinoma/Phase 2/Phase 3
stage IVA oropharyngeal squamous cell carcinoma/Phase 3
stage IVA oropharyngeal squamous cell carcinoma/Unspecified phase
stage IVB cervical carcinoma/Phase 2
stage IVB oropharyngeal squamous cell carcinoma/Phase 1
stage IVB oropharyngeal squamous cell carcinoma/Phase 1/Phase 2
stage IVB oropharyngeal squamous cell carcinoma/Phase 2
stage IVB oropharyngeal squamous cell carcinoma/Phase 2/Phase 3
stage IVB oropharyngeal squamous cell carcinoma/Unspecified phase
stage IVC oropharyngeal squamous cell carcinoma/Phase 1

© 2000-2014 QIAGEN. All rights reserved.

				stage IVC oropharyngeal squamous cell carcinoma/Phase 1/Phase 2<br>stage IVC oropharyngeal squamous cell carcinoma/Phase 2<br>stage IVC oropharyngeal squamous cell carcinoma/Phase 3<br>thymic carcinoma/Phase 2<br>thymoma/Phase 2<br>tongue cancer/Phase 1<br>tongue cancer/Phase 1/Phase 2<br>tongue cancer/Phase 2<br>tongue cancer/Phase 2/Phase 3<br>tongue cancer/Unspecified phase<br>triple negative basal like breast carcinoma/Phase 1/Phase 2<br>tumor/Phase 1<br>undifferentiated nasopharyngeal carcinoma/Phase 1<br>undifferentiated nasopharyngeal carcinoma/Phase 1/Phase 2
cetuximab	EGFR	inhibitor	Erbitux	adenocarcinoma/Phase 2<br>adult solid tumor/Phase 1<br>adult solid tumor/Phase 1/Phase 2<br>advanced cancer/Phase 1<br>advanced cancer/Phase 2<br>advanced colorectal cancer/Phase 2<br>advanced non-small-cell lung cancer/Phase 2<br>advanced solid tumor/Phase 1<br>anal cancer/Phase 1<br>anal cancer/Phase 2<br>anaplastic astrocytoma/Phase 1<br>appendix carcinoma/Phase 1<br>biliary tract cancer/Phase 2<br>bladder cancer/Phase 2<br>BRAF mutation negative colon adenocarcinoma/Phase 2<br>BRAF mutation negative KRAS mutation negative colon adenocarcinoma/Phase 2<br>BRAF mutation negative KRAS mutation negative colorectal cancer/Phase 2<br>BRAF mutation negative KRAS mutation negative NRAS mutation negative colon adenocarcinoma/Phase 2<br>BRAF mutation negative KRAS mutation negative NRAS mutation negative colorectal adenocarcinoma/Phase 2<br>BRAF mutation negative KRAS mutation negative NRAS mutation negative colorectal cancer/Phase 1/Phase 2<br>BRAF mutation negative KRAS mutation negative NRAS mutation negative colorectal cancer/Phase 2<br>BRAF mutation negative KRAS mutation negative NRAS mutation negative PIK3CA mutation negative colorectal cancer/Phase 1/Phase 2<br>BRAF mutation negative KRAS mutation negative NRAS mutation negative rectal adenocarcinoma/Phase 2<br>BRAF mutation negative KRAS mutation negative rectal adenocarcinoma/Phase 2<br>BRAF mutation negative rectal adenocarcinoma/Phase 2<br>BRAF mutation positive colorectal cancer/Phase 1/Phase 2<br>BRAF mutation positive colorectal cancer/Phase 2<br>BRAF V600 mutation positive cancer/Phase 2<br>BRAF V600 mutation positive KRAS mutation negative cancer/Phase 1<br>BRAF V600 mutation positive KRAS mutation negative malignant neoplasm/Phase 1<br>BRAF V600 mutation positive KRAS mutation negative RNF43 mutation positive colorectal cancer/Phase 1/Phase 2<br>BRAF V600 mutation positive KRAS mutation negative RNF43 mutation positive RSPO fusion positive colorectal cancer/Phase 1/Phase 2<br>BRAF V600 mutation positive KRAS mutation negative RSPO fusion positive colorectal cancer/Phase 1/Phase 2<br>BRAF V600 mutation positive KRAS mutation negative solid tumor/Phase 1<br>BRAF V600 mutation positive multiple myeloma/Phase 2<br>BRAF V600 mutation positive solid tumor/Phase 2<br>BRAF V600E positive KRAS mutation negative colorectal

© 2000-2014 QIAGEN. All rights reserved.

cancer/Phase 1
breast cancer/Phase 1
breast cancer/Phase 2
c-MET amplified solid tumor/Phase 1
C-MET positive head and neck squamous cell carcinoma/Phase 1/Phase 2
c-MET positive KRAS mutation negative NRAS mutation negative colorectal cancer/Phase 1/Phase 2
cancer/Phase 1
cancer/Phase 2
carcinoma/Phase 2
Castleman's disease/Phase 1
CDKN2A mutation negative oropharyngeal carcinoma/Phase 2
CDKN2A negative oropharyngeal squamous cell carcinoma/Phase 2
CDKN2A positive oropharyngeal carcinoma/Phase 2
CDKN2A positive oropharyngeal squamous cell carcinoma/Phase 2
CDKN2A positive oropharyngeal squamous cell carcinoma/Phase 3
cervical adenocarcinoma/Phase 1
cervical adenosquamous carcinoma/Phase 1
cervical cancer/Phase 1
cervical cancer/Phase 2
cervical small cell carcinoma/Phase 1
cervical squamous cell carcinoma/Phase 1
cervical squamous cell carcinoma/Phase 2
cholangiocarcinoma/Phase 2
colon adenocarcinoma/Phase 1
colon adenocarcinoma/Phase 1/Phase 2
colon adenocarcinoma/Phase 3
colon cancer/Phase 0
colon cancer/Phase 1
colon cancer/Phase 1/Phase 2
colon cancer/Phase 2
colon cancer/Phase 3
colon carcinoma/Phase 1
colon tumor/Phase 1
colorectal adenocarcinoma/Phase 1
colorectal cancer/Phase 1
colorectal cancer/Phase 1/Phase 2
colorectal cancer/Phase 2
colorectal cancer/Phase 2/Phase 3
colorectal cancer/Phase 3
colorectal cancer/Phase 4
colorectal cancer/Unspecified phase
colorectal carcinoma/Phase 1
colorectal carcinoma/Phase 1/Phase 2
colorectal carcinoma/Phase 2
colorectal tumor/Phase 1
colorectal tumor/Phase 2
dermatological disorder/Phase 2
EGFR expressing colorectal cancer/Phase 2
EGFR expressing epithelioid malignant pleural mesothelioma/Phase 2
EGFR expressing head and neck squamous cell carcinoma/Phase 2
EGFR expressing KRAS mutation negative colorectal adenocarcinoma/Phase 4
EGFR mutation positive esophageal squamous cell carcinoma/Phase 2
EGFR positive head and neck cancer/Phase 2
endometrial cancer/Phase 2
Erdheim-Chester disease/Phase 1
esophageal adenocarcinoma/Phase 2
esophageal adenocarcinoma/Unspecified phase
esophageal cancer/Phase 1
esophageal cancer/Phase 1/Phase 2
esophageal cancer/Phase 2
esophageal cancer/Phase 2/Phase 3
esophageal cancer/Phase 3
esophageal carcinoma/Phase 2
esophageal squamous cell cancer/Phase 2
esophageal squamous cell carcinoma/Phase 2
esophagogastric junction cancer/Phase 2
extrahepatic bile duct cancer/Phase 1
extrahepatic cholangiocarcinoma/Phase 2
fallopian tube neoplasm/Phase 2

© 2000-2014 QIAGEN. All rights reserved.

gall bladder adenocarcinoma/Phase 2
gallbladder cancer/Phase 1
gastric adenocarcinoma/Phase 2
gastric cancer/Phase 1
gastric cancer/Phase 1/Phase 2
gastric cancer/Phase 2
gastric cancer/Phase 3
gastric cancer/Unspecified phase
gastrointestinal stromal tumor/Phase 1
glioblastoma cancer/Phase 2
glioblastoma/Phase 1
head and neck adenoid cystic carcinoma/Phase 1/Phase 2
head and neck cancer/Phase 0
head and neck cancer/Phase 1
head and neck cancer/Phase 1/Phase 2
head and neck cancer/Phase 2
head and neck cancer/Phase 3
head and neck cancer/Phase 4
head and neck cancer/Unspecified phase
head and neck carcinoma/Phase 2
head and neck squamous cell cancer/Phase 1
head and neck squamous cell cancer/Phase 2
head and neck squamous cell cancer/Phase 3
head and neck squamous cell cancer/Unspecified phase
head and neck squamous cell carcinoma/Phase 1
head and neck squamous cell carcinoma/Phase 1/Phase 2
head and neck squamous cell carcinoma/Phase 2
head and neck squamous cell carcinoma/Phase 3
head and neck tumor/Phase 1
head and neck tumor/Phase 1/Phase 2
head and neck tumor/Phase 2
hepatocellular carcinoma/Phase 2
HER2 negative hormone receptor negative breast cancer/Phase 1
HER2 negative hormone receptor negative breast cancer/Phase 2
HER2 positive EGFR positive breast cancer/Phase 1
HER2 positive esophagogastric junction cancer/Phase 1/Phase 2
HER2 positive gastric cancer/Phase 1/Phase 2
HER2 positive metastatic breast cancer/Phase 1/Phase 2
HER3 positive carcinoma/Phase 1
hormone refractory prostate cancer/Phase 2
hypopharyngeal cancer/Phase 2
hypopharyngeal squamous cell carcinoma/Phase 1
hypopharyngeal squamous cell carcinoma/Phase 1/Phase 2
hypopharyngeal squamous cell carcinoma/Phase 2
hypopharyngeal squamous cell carcinoma/Phase 2/Phase 3
hypopharyngeal squamous cell carcinoma/Phase 3
hypopharyngeal squamous cell carcinoma/Phase 4
hypopharyngeal squamous cell carcinoma/Unspecified phase
infection/Phase 2
KRAS codon 12 mutation positive colorectal cancer/Phase 2
KRAS codon 13 mutation positive colorectal cancer/Phase 2
KRAS codon 61 mutation positive colorectal cancer/Phase 2
KRAS mutation negative adenocarcinoma of the colon/Phase 1
KRAS mutation negative adenocarcinoma of the colon/Phase 2
KRAS mutation negative adenocarcinoma of the rectum/Phase 1
KRAS mutation negative adenocarcinoma of the rectum/Phase 2
KRAS mutation negative and NRAS mutation negative colorectal cancer/Phase 1/Phase 2
KRAS mutation negative and NRAS mutation negative colorectal cancer/Phase 2
KRAS mutation negative and NRAS mutation negative colorectal cancer/Phase 3
KRAS mutation negative colon carcinoma/Phase 3
KRAS mutation negative colorectal

© 2000-2014 QIAGEN. All rights reserved.

adenocarcinoma/Phase 2
KRAS mutation negative head and neck squamous cell carcinoma/Phase 1
KRAS mutation negative NRAS mutation negative colon adenocarcinoma/Phase 2
KRAS mutation negative NRAS mutation negative colon adenocarcinoma/Phase 3
KRAS mutation negative NRAS mutation negative colorectal adenocarcinoma/Phase 1/Phase 2
KRAS mutation negative NRAS mutation negative rectal adenocarcinoma/Phase 2
KRAS mutation negative NRAS mutation negative rectal adenocarcinoma/Phase 3
KRAS mutation negative penis cancer/Phase 2
KRAS mutation negative penis squamous cell carcinoma/Phase 2
KRAS mutation negative rectal cancer/Phase 2
KRAS mutation negative rectal carcinoma/Phase 3
KRAS mutation positive colorectal cancer/Phase 2
KRAS wild-type liver metastasis associated with KRAS wild-type metastatic colorectal cancer/Phase 2
KRAS wild-type liver metastasis/Phase 2
KRAS wild-type metastatic colorectal cancer/Phase 1
KRAS wild-type metastatic colorectal cancer/Phase 2
KRAS wild-type positive colorectal cancer/Phase 1
KRAS wild-type positive colorectal cancer/Phase 2
large cell lung cancer/Phase 2
laryngeal cancer/Phase 1
laryngeal cancer/Phase 2
laryngeal carcinoma/Phase 2
laryngeal squamous cell carcinoma/Phase 1
laryngeal squamous cell carcinoma/Phase 1/Phase 2
laryngeal squamous cell carcinoma/Phase 2
laryngeal squamous cell carcinoma/Phase 2/Phase 3
laryngeal squamous cell carcinoma/Phase 3
laryngeal squamous cell carcinoma/Phase 4
laryngeal squamous cell carcinoma/Unspecified phase
laryngeal tumor/Phase 2
laryngeal verrucous carcinoma/Phase 1
laryngeal verrucous carcinoma/Phase 1/Phase 2
laryngeal verrucous carcinoma/Phase 2
laryngeal verrucous carcinoma/Phase 2/Phase 3
laryngeal verrucous carcinoma/Unspecified phase
liver cancer/Phase 1
liver cancer/Phase 2
liver lesion associated with metastatic colorectal cancer/Phase 2
liver metastasis associated with EGFR-expressing KRAS mutation-negative metastatic colorectal cancer/Phase 2
liver metastasis associated with KRAS wild-type metastatic colorectal cancer/Phase 2
liver metastasis associated with metastatic colorectal cancer/Phase 2
liver metastasis associated with metastatic KRAS-mutation-positive colorectal cancer/Phase 2
liver metastasis/Phase 0
liver metastasis/Phase 2
liver metastasis/Unspecified phase
loco-regionally advanced squamous cell carcinoma of the head and neck/Approved
lung adenocarcinoma/Phase 1/Phase 2
lung adenosquamous cancer/Phase 2
lung cancer/Phase 1
lung cancer/Phase 1/Phase 2
lung cancer/Phase 2
lung cancer/Phase 3
lung tumor/Phase 2
lymphangioleiomyomatosis/Phase 1

© 2000-2014 QIAGEN. All rights reserved.

malignant glioma/Phase 2
malignant pleural effusion/Phase 2
malignant solid tumor/Phase 1
malignant tumor/Phase 3
mammary tumor/Phase 2
metastasis/Phase 1
metastasis/Phase 1/Phase 2
metastasis/Phase 2
metastasis/Phase 3
metastasis/Phase 4
metastatic breast cancer/Phase 2
metastatic colorectal adenocarcinoma/Phase 1/Phase 2
metastatic colorectal cancer/Phase 1
metastatic colorectal cancer/Phase 1/Phase 2
metastatic colorectal cancer/Phase 2
metastatic colorectal cancer/Phase 3
metastatic colorectal cancer/Phase 4
metastatic colorectal cancer/Unspecified phase
metastatic colorectal carcinoma/Phase 1/Phase 2
metastatic head and neck squamous cell carcinoma/Phase 1
metastatic head and neck squamous cell carcinoma/Phase 1/Phase 2
metastatic head and neck squamous cell carcinoma/Phase 2
metastatic KRAS mutation negative colorectal adenocarcinoma/Phase 2
metastatic malignant colorectal neoplasm/Approved
metastatic non-small-cell lung cancer/Phase 1
metastatic non-small-cell lung cancer/Phase 1/Phase 2
metastatic non-small-cell lung cancer/Phase 2
metastatic non-small-cell lung cancer/Phase 2/Phase 3
metastatic non-small-cell lung cancer/Phase 3
metastatic pancreatic cancer/Phase 1
metastatic pancreatic cancer/Phase 1/Phase 2
metastatic pancreatic cancer/Phase 2
metastatic solid tumor/Phase 1
metastatic solid tumor/Phase 1/Phase 2
metastatic squamous cell carcinoma of the hypopharynx/Phase 1
metastatic squamous cell carcinoma of the hypopharynx/Phase 1/Phase 2
metastatic squamous cell carcinoma of the hypopharynx/Phase 2
metastatic squamous cell carcinoma of the larynx/Phase 1
metastatic squamous cell carcinoma of the larynx/Phase 1/Phase 2
metastatic squamous cell carcinoma of the larynx/Phase 2
metastatic squamous cell carcinoma of the oral cavity/Phase 1
metastatic squamous cell carcinoma of the oral cavity/Phase 1/Phase 2
metastatic squamous cell carcinoma of the oral cavity/Phase 2
mucinous adenocarcinoma of colon/Phase 0
mucinous adenocarcinoma of colon/Phase 1
mucinous adenocarcinoma of colon/Phase 2
mucinous adenocarcinoma of rectum/Phase 0
mucinous adenocarcinoma of rectum/Phase 1
multiple myeloma/Phase 2
nasopharyngeal carcinoma/Phase 1/Phase 2
nasopharyngeal carcinoma/Phase 2
neoplasia/Phase 2
neuralgia/Phase 2
non small cell lung adenocarcinoma/Phase 2
non-small cell lung cancer/Phase 1
non-small cell lung cancer/Phase 1/Phase 2
non-small cell lung cancer/Phase 2
non-small cell lung cancer/Phase

© 2000-2014 QIAGEN. All rights reserved.

2/Phase 3
non-small cell lung cancer/Phase 3
non-squamous non-small cell lung cancer/Phase 2
oral cancer/Phase 1
oral squamous cell carcinoma/Phase 1
oral squamous cell carcinoma/Phase 2
oropharyngeal cancer/Phase 2
oropharyngeal squamous-cell carcinoma/Phase 1
oropharyngeal squamous-cell carcinoma/Phase 1/Phase 2
oropharyngeal squamous-cell carcinoma/Phase 2
oropharyngeal squamous-cell carcinoma/Phase 3
oropharyngeal squamous-cell carcinoma/Phase 4
ovarian cancer/Phase 2
pancreatic adenocarcinoma/Phase 2
pancreatic cancer/Phase 1
pancreatic cancer/Phase 1/Phase 2
pancreatic cancer/Phase 2
pancreatic cancer/Phase 3
pancreatic cancer/Unspecified phase
peritoneal cavity cancer/Phase 2
peritoneal tumor/Phase 2
pharyngeal cancer/Phase 1
pharyngeal tumor/Phase 2
precancerous condition/Phase 2
precancerous condition/Unspecified phase
primary glioblastoma/Phase 1/Phase 2
primary head and neck squamous cell carcinoma/Phase 1
primary laryngeal squamous cell carcinoma/Phase 1
primary oral squamous cell carcinoma/Phase 1
primary peritoneal cancer/Phase 2
prostate cancer/Phase 2
prostatic tumor/Phase 2
radiation toxicity/Phase 3
RAS mutation negative colon adenocarcinoma/Phase 2
RAS mutation negative colorectal adenocarcinoma/Phase 2
RAS mutation negative colorectal cancer/Phase 2
RAS mutation negative rectal adenocarcinoma/Phase 2
rectal adenocarcinoma/Phase 1
rectal adenocarcinoma/Phase 1/Phase 2
rectal adenocarcinoma/Phase 2
rectum cancer/Phase 0
rectum cancer/Phase 1
rectum cancer/Phase 1/Phase 2
rectum cancer/Phase 2
rectum cancer/Phase 3
rectum carcinoma/Phase 1
recurrent cancer/Phase 1
recurrent cervical carcinoma/Phase 2
recurrent laryngeal squamous cell carcinoma/Phase 1
recurrent laryngeal squamous cell carcinoma/Phase 1/Phase 2
recurrent laryngeal squamous cell carcinoma/Phase 2
recurrent loco-regionally advanced squamous cell carcinoma of the head and neck/Approved
recurrent oropharyngeal squamous cell carcinoma/Phase 1
recurrent oropharyngeal squamous cell carcinoma/Phase 1/Phase 2
recurrent oropharyngeal squamous cell carcinoma/Phase 2
recurrent rectal cancer/Phase 0
recurrent rectal cancer/Phase 1
recurrent rectal cancer/Phase 1/Phase 2
recurrent rectal cancer/Phase 2
recurrent rectal cancer/Phase 3
renal cancer/Phase 1
renal parenchyma cancer/Phase 1/Phase 2
salivary gland cancer/Phase 1
salivary gland cancer/Phase 1/Phase 2
salivary gland cancer/Phase 2
salivary gland carcinoma/Phase 1
salivary gland carcinoma/Phase 1/Phase 2
salivary gland carcinoma/Phase 2
sarcoma/Phase 1
sarcoma/Phase 2
signet ring adenocarcinoma of the colon/Phase 0
signet ring adenocarcinoma of the colon/Phase 1

© 2000-2014 QIAGEN. All rights reserved.

signet ring adenocarcinoma of the colon/Phase 2
signet ring adenocarcinoma of the rectum/Phase 0
signet ring adenocarcinoma of the rectum/Phase 1
skin cancer/Unspecified phase
skin squamous cell carcinoma/Unspecified phase
small intestine cancer/Phase 1
solid tumor/Phase 1
solid tumor/Phase 1/Phase 2
squamous cell carcinoma of nasopharynx/Phase 1
squamous cell carcinoma of nasopharynx/Phase 1/Phase 2
squamous cell carcinoma of nasopharynx/Phase 2
squamous cell carcinoma of the oral cavity/Phase 1/Phase 2
squamous cell carcinoma of the oral cavity/Phase 2
squamous cell carcinoma of the oral cavity/Phase 3
squamous cell carcinoma of the oral cavity/Phase 4
squamous cell carcinoma/Phase 1
squamous cell carcinoma/Phase 1/Phase 2
squamous cell carcinoma/Phase 2
squamous cell lung cancer/Phase 1
squamous cell lung cancer/Phase 2
stage IB2 cervical carcinoma/Phase 2
stage III head and neck squamous cell carcinoma/Phase 3
stage III oropharyngeal squamous cell carcinoma/Phase 1
stage III oropharyngeal squamous cell carcinoma/Phase 1/Phase 2
stage III oropharyngeal squamous cell carcinoma/Phase 2
stage III oropharyngeal squamous cell carcinoma/Phase 2/Phase 3
stage III oropharyngeal squamous cell carcinoma/Phase 3
stage III oropharyngeal squamous cell carcinoma/Phase 4
stage III oropharyngeal squamous cell carcinoma/Unspecified phase
stage IV gastric cancer/Phase 1
stage IV head and neck squamous cell carcinoma/Phase 2
stage IV head and neck squamous cell carcinoma/Phase 3
stage IV oropharyngeal squamous cell carcinoma/Phase 1
stage IV oropharyngeal squamous cell carcinoma/Phase 1/Phase 2
stage IV oropharyngeal squamous cell carcinoma/Phase 2
stage IV oropharyngeal squamous cell carcinoma/Phase 4
stage IVA oropharyngeal squamous cell carcinoma/Phase 1
stage IVA oropharyngeal squamous cell carcinoma/Phase 1/Phase 2
stage IVA oropharyngeal squamous cell carcinoma/Phase 2
stage IVA oropharyngeal squamous cell carcinoma/Phase 2/Phase 3
stage IVA oropharyngeal squamous cell carcinoma/Phase 3
stage IVA oropharyngeal squamous cell carcinoma/Unspecified phase
stage IVB cervical carcinoma/Phase 2
stage IVB oropharyngeal squamous cell carcinoma/Phase 1
stage IVB oropharyngeal squamous cell carcinoma/Phase 1/Phase 2
stage IVB oropharyngeal squamous cell carcinoma/Phase 2
stage IVB oropharyngeal squamous cell carcinoma/Phase 2/Phase 3
stage IVB oropharyngeal squamous cell carcinoma/Unspecified phase
stage IVC oropharyngeal squamous cell carcinoma/Phase 1
stage IVC oropharyngeal squamous cell carcinoma/Phase 1/Phase 2
stage IVC oropharyngeal squamous cell carcinoma/Phase 2
stage IVC oropharyngeal squamous cell carcinoma/Phase 3
thymic carcinoma/Phase 2
thymoma/Phase 2
tongue cancer/Phase 1
tongue cancer/Phase 1/Phase 2

© 2000-2014 QIAGEN. All rights reserved.

				tongue cancer/Phase 2<br>tongue cancer/Phase 2/Phase 3<br>tongue cancer/Unspecified phase<br>triple negative basal like breast carcinoma/Phase 1/Phase 2<br>tumor/Phase 1<br>undifferentiated nasopharyngeal carcinoma/Phase 1<br>undifferentiated nasopharyngeal carcinoma/Phase 1/Phase 2
cetuximab	EGFR	antagonist	Erbitux	adenocarcinoma/Phase 2<br>adult solid tumor/Phase 1<br>adult solid tumor/Phase 1/Phase 2<br>advanced cancer/Phase 1<br>advanced cancer/Phase 2<br>advanced colorectal cancer/Phase 2<br>advanced non-small-cell lung cancer/Phase 2<br>advanced solid tumor/Phase 1<br>anal cancer/Phase 1<br>anal cancer/Phase 2<br>anaplastic astrocytoma/Phase 1<br>appendix carcinoma/Phase 1<br>biliary tract cancer/Phase 2<br>bladder cancer/Phase 2<br>BRAF mutation negative colon adenocarcinoma/Phase 2<br>BRAF mutation negative KRAS mutation negative colon adenocarcinoma/Phase 2<br>BRAF mutation negative KRAS mutation negative colorectal cancer/Phase 2<br>BRAF mutation negative KRAS mutation negative NRAS mutation negative colon adenocarcinoma/Phase 2<br>BRAF mutation negative KRAS mutation negative NRAS mutation negative colorectal adenocarcinoma/Phase 2<br>BRAF mutation negative KRAS mutation negative NRAS mutation negative colorectal cancer/Phase 1/Phase 2<br>BRAF mutation negative KRAS mutation negative NRAS mutation negative colorectal cancer/Phase 2<br>BRAF mutation negative KRAS mutation negative NRAS mutation negative PIK3CA mutation negative colorectal cancer/Phase 1/Phase 2<br>BRAF mutation negative KRAS mutation negative NRAS mutation negative rectal adenocarcinoma/Phase 2<br>BRAF mutation negative KRAS mutation negative rectal adenocarcinoma/Phase 2<br>BRAF mutation negative rectal adenocarcinoma/Phase 2<br>BRAF mutation positive colorectal cancer/Phase 1/Phase 2<br>BRAF mutation positive colorectal cancer/Phase 2<br>BRAF V600 mutation positive cancer/Phase 2<br>BRAF V600 mutation positive KRAS mutation negative cancer/Phase 1<br>BRAF V600 mutation positive KRAS mutation negative malignant neoplasm/Phase 1<br>BRAF V600 mutation positive KRAS mutation negative RNF43 mutation positive colorectal cancer/Phase 1/Phase 2<br>BRAF V600 mutation positive KRAS mutation negative RNF43 mutation positive RSPO fusion positive colorectal cancer/Phase 1/Phase 2<br>BRAF V600 mutation positive KRAS mutation negative RSPO fusion positive colorectal cancer/Phase 1/Phase 2<br>BRAF V600 mutation positive KRAS mutation negative solid tumor/Phase 1<br>BRAF V600 mutation positive multiple myeloma/Phase 2<br>BRAF V600 mutation positive solid tumor/Phase 2<br>BRAF V600E positive KRAS mutation negative colorectal cancer/Phase 1<br>breast cancer/Phase 1<br>breast cancer/Phase 2<br>c-MET amplified solid tumor/Phase 1<br>C-MET positive head and neck squamous cell carcinoma/Phase 1/Phase 2<br>c-MET positive KRAS mutation negative NRAS mutation negative colorectal cancer/Phase 1/Phase 2

© 2000-2014 QIAGEN. All rights reserved.
INGENUITY SYSTEMS

cancer/Phase 1
cancer/Phase 2
carcinoma/Phase 2
Castleman's disease/Phase 1
CDKN2A mutation negative oropharyngeal carcinoma/Phase 2
CDKN2A negative oropharyngeal squamous cell carcinoma/Phase 2
CDKN2A positive oropharyngeal carcinoma/Phase 2
CDKN2A positive oropharyngeal squamous cell carcinoma/Phase 2
CDKN2A positive oropharyngeal squamous cell carcinoma/Phase 3
cervical adenocarcinoma/Phase 1
cervical adenosquamous carcinoma/Phase 1
cervical cancer/Phase 1
cervical cancer/Phase 2
cervical small cell carcinoma/Phase 1
cervical squamous cell carcinoma/Phase 1
cervical squamous cell carcinoma/Phase 2
cholangiocarcinoma/Phase 2
colon adenocarcinoma/Phase 1
colon adenocarcinoma/Phase 1/Phase 2
colon adenocarcinoma/Phase 3
colon cancer/Phase 0
colon cancer/Phase 1
colon cancer/Phase 1/Phase 2
colon cancer/Phase 2
colon cancer/Phase 3
colon carcinoma/Phase 1
colon tumor/Phase 1
colorectal adenocarcinoma/Phase 1
colorectal cancer/Phase 1
colorectal cancer/Phase 1/Phase 2
colorectal cancer/Phase 2
colorectal cancer/Phase 2/Phase 3
colorectal cancer/Phase 3
colorectal cancer/Phase 4
colorectal cancer/Unspecified phase
colorectal carcinoma/Phase 1
colorectal carcinoma/Phase 1/Phase 2
colorectal carcinoma/Phase 2
colorectal tumor/Phase 1
colorectal tumor/Phase 2
dermatological disorder/Phase 2
EGFR expressing colorectal cancer/Phase 2
EGFR expressing epithelioid malignant pleural mesothelioma/Phase 2
EGFR expressing head and neck squamous cell carcinoma/Phase 2
EGFR expressing KRAS mutation negative colorectal adenocarcinoma/Phase 4
EGFR mutation positive esophageal squamous cell carcinoma/Phase 2
EGFR positive head and neck cancer/Phase 2
endometrial cancer/Phase 2
Erdheim-Chester disease/Phase 1
esophageal adenocarcinoma/Phase 2
esophageal adenocarcinoma/Unspecified phase
esophageal cancer/Phase 1
esophageal cancer/Phase 1/Phase 2
esophageal cancer/Phase 2
esophageal cancer/Phase 2/Phase 3
esophageal cancer/Phase 3
esophageal carcinoma/Phase 2
esophageal squamous cell cancer/Phase 2
esophageal squamous cell carcinoma/Phase 2
esophagogastric junction cancer/Phase 2
extrahepatic bile duct cancer/Phase 1
extrahepatic cholangiocarcinoma/Phase 2
fallopian tube neoplasm/Phase 2
gall bladder adenocarcinoma/Phase 2
gallbladder cancer/Phase 1
gastric adenocarcinoma/Phase 2
gastric cancer/Phase 1
gastric cancer/Phase 1/Phase 2
gastric cancer/Phase 2
gastric cancer/Phase 3
gastric cancer/Unspecified phase
gastrointestinal stromal tumor/Phase 1
glioblastoma cancer/Phase 2

© 2000-2014 QIAGEN. All rights reserved.

glioblastoma/Phase 1
head and neck adenoid cystic carcinoma/Phase 1/Phase 2
head and neck cancer/Phase 0
head and neck cancer/Phase 1
head and neck cancer/Phase 1/Phase 2
head and neck cancer/Phase 2
head and neck cancer/Phase 3
head and neck cancer/Phase 4
head and neck cancer/Unspecified phase
head and neck carcinoma/Phase 2
head and neck squamous cell cancer/Phase 1
head and neck squamous cell cancer/Phase 2
head and neck squamous cell cancer/Phase 3
head and neck squamous cell cancer/Unspecified phase
head and neck squamous cell carcinoma/Phase 1
head and neck squamous cell carcinoma/Phase 1/Phase 2
head and neck squamous cell carcinoma/Phase 2
head and neck squamous cell carcinoma/Phase 3
head and neck tumor/Phase 1
head and neck tumor/Phase 1/Phase 2
head and neck tumor/Phase 2
hepatocellular carcinoma/Phase 2
HER2 negative hormone receptor negative breast cancer/Phase 1
HER2 negative hormone receptor negative breast cancer/Phase 2
HER2 positive EGFR positive breast cancer/Phase 1
HER2 positive esophagogastric junction cancer/Phase 1/Phase 2
HER2 positive gastric cancer/Phase 1/Phase 2
HER2 positive metastatic breast cancer/Phase 1/Phase 2
HER3 positive carcinoma/Phase 1
hormone refractory prostate cancer/Phase 2
hypopharyngeal cancer/Phase 2
hypopharyngeal squamous cell carcinoma/Phase 1
hypopharyngeal squamous cell carcinoma/Phase 1/Phase 2
hypopharyngeal squamous cell carcinoma/Phase 2
hypopharyngeal squamous cell carcinoma/Phase 2/Phase 3
hypopharyngeal squamous cell carcinoma/Phase 3
hypopharyngeal squamous cell carcinoma/Phase 4
hypopharyngeal squamous cell carcinoma/Unspecified phase
infection/Phase 2
KRAS codon 12 mutation positive colorectal cancer/Phase 2
KRAS codon 13 mutation positive colorectal cancer/Phase 2
KRAS codon 61 mutation positive colorectal cancer/Phase 2
KRAS mutation negative adenocarcinoma of the colon/Phase 1
KRAS mutation negative adenocarcinoma of the colon/Phase 2
KRAS mutation negative adenocarcinoma of the rectum/Phase 1
KRAS mutation negative adenocarcinoma of the rectum/Phase 2
KRAS mutation negative and NRAS mutation negative colorectal cancer/Phase 1/Phase 2
KRAS mutation negative and NRAS mutation negative colorectal cancer/Phase 2
KRAS mutation negative and NRAS mutation negative colorectal cancer/Phase 3
KRAS mutation negative colon carcinoma/Phase 3
KRAS mutation negative colorectal adenocarcinoma/Phase 2
KRAS mutation negative head and neck squamous cell carcinoma/Phase 1
KRAS mutation negative NRAS mutation negative colon adenocarcinoma/Phase 2
KRAS mutation negative NRAS mutation negative colon adenocarcinoma/Phase 3
KRAS mutation negative NRAS

© 2000-2014 QIAGEN. All rights reserved.

mutation negative colorectal adenocarcinoma/Phase 1/Phase 2
KRAS mutation negative NRAS mutation negative rectal adenocarcinoma/Phase 2
KRAS mutation negative NRAS mutation negative rectal adenocarcinoma/Phase 3
KRAS mutation negative penis cancer/Phase 2
KRAS mutation negative penis squamous cell carcinoma/Phase 2
KRAS mutation negative rectal cancer/Phase 2
KRAS mutation negative rectal carcinoma/Phase 3
KRAS mutation positive colorectal cancer/Phase 2
KRAS wild-type liver metastasis associated with KRAS wild-type metastatic colorectal cancer/Phase 2
KRAS wild-type liver metastasis/Phase 2
KRAS wild-type metastatic colorectal cancer/Phase 1
KRAS wild-type metastatic colorectal cancer/Phase 2
KRAS wild-type positive colorectal cancer/Phase 1
KRAS wild-type positive colorectal cancer/Phase 2
large cell lung cancer/Phase 2
laryngeal cancer/Phase 1
laryngeal cancer/Phase 2
laryngeal carcinoma/Phase 2
laryngeal squamous cell carcinoma/Phase 1
laryngeal squamous cell carcinoma/Phase 1/Phase 2
laryngeal squamous cell carcinoma/Phase 2
laryngeal squamous cell carcinoma/Phase 2/Phase 3
laryngeal squamous cell carcinoma/Phase 3
laryngeal squamous cell carcinoma/Phase 4
laryngeal squamous cell carcinoma/Unspecified phase
laryngeal tumor/Phase 2
laryngeal verrucous carcinoma/Phase 1
laryngeal verrucous carcinoma/Phase 1/Phase 2
laryngeal verrucous carcinoma/Phase 2
laryngeal verrucous carcinoma/Phase 2/Phase 3
laryngeal verrucous carcinoma/Unspecified phase
liver cancer/Phase 1
liver cancer/Phase 2
liver lesion associated with metastatic colorectal cancer/Phase 2
liver metastasis associated with EGFR-expressing KRAS mutation-negative metastatic colorectal cancer/Phase 2
liver metastasis associated with KRAS wild-type metastatic colorectal cancer/Phase 2
liver metastasis associated with metastatic colorectal cancer/Phase 2
liver metastasis associated with metastatic KRAS-mutation-positive colorectal cancer/Phase 2
liver metastasis/Phase 0
liver metastasis/Phase 2
liver metastasis/Unspecified phase
loco-regionally advanced squamous cell carcinoma of the head and neck/Approved
lung adenocarcinoma/Phase 1/Phase 2
lung adenosquamous cancer/Phase 2
lung cancer/Phase 1
lung cancer/Phase 1/Phase 2
lung cancer/Phase 2
lung cancer/Phase 3
lung tumor/Phase 2
lymphangioleiomyomatosis/Phase 1
malignant glioma/Phase 2
malignant pleural effusion/Phase 2
malignant solid tumor/Phase 1
malignant tumor/Phase 3
mammary tumor/Phase 2
metastasis/Phase 1
metastasis/Phase 1/Phase 2
metastasis/Phase 2
metastasis/Phase 3
metastasis/Phase 4
metastatic breast cancer/Phase 2

© 2000-2014 QIAGEN. All rights reserved.

metastatic colorectal adenocarcinoma/Phase 1/Phase 2
metastatic colorectal cancer/Phase 1
metastatic colorectal cancer/Phase 1/Phase 2
metastatic colorectal cancer/Phase 2
metastatic colorectal cancer/Phase 3
metastatic colorectal cancer/Phase 4
metastatic colorectal cancer/Unspecified phase
metastatic colorectal carcinoma/Phase 1/Phase 2
metastatic head and neck squamous cell carcinoma/Phase 1
metastatic head and neck squamous cell carcinoma/Phase 1/Phase 2
metastatic head and neck squamous cell carcinoma/Phase 2
metastatic KRAS mutation negative colorectal adenocarcinoma/Phase 2
metastatic malignant colorectal neoplasm/Approved
metastatic non-small-cell lung cancer/Phase 1
metastatic non-small-cell lung cancer/Phase 1/Phase 2
metastatic non-small-cell lung cancer/Phase 2
metastatic non-small-cell lung cancer/Phase 2/Phase 3
metastatic non-small-cell lung cancer/Phase 3
metastatic pancreatic cancer/Phase 1
metastatic pancreatic cancer/Phase 1/Phase 2
metastatic pancreatic cancer/Phase 2
metastatic solid tumor/Phase 1
metastatic solid tumor/Phase 1/Phase 2
metastatic squamous cell carcinoma of the hypopharynx/Phase 1
metastatic squamous cell carcinoma of the hypopharynx/Phase 1/Phase 2
metastatic squamous cell carcinoma of the hypopharynx/Phase 2
metastatic squamous cell carcinoma of the larynx/Phase 1
metastatic squamous cell carcinoma of the larynx/Phase 1/Phase 2
metastatic squamous cell carcinoma of the larynx/Phase 2
metastatic squamous cell carcinoma of the oral cavity/Phase 1
metastatic squamous cell carcinoma of the oral cavity/Phase 1/Phase 2
metastatic squamous cell carcinoma of the oral cavity/Phase 2
mucinous adenocarcinoma of colon/Phase 0
mucinous adenocarcinoma of colon/Phase 1
mucinous adenocarcinoma of colon/Phase 2
mucinous adenocarcinoma of rectum/Phase 0
mucinous adenocarcinoma of rectum/Phase 1
multiple myeloma/Phase 2
nasopharyngeal carcinoma/Phase 1/Phase 2
nasopharyngeal carcinoma/Phase 2
neoplasia/Phase 2
neuralgia/Phase 2
non small cell lung adenocarcinoma/Phase 2
non-small cell lung cancer/Phase 1
non-small cell lung cancer/Phase 1/Phase 2
non-small cell lung cancer/Phase 2
non-small cell lung cancer/Phase 2/Phase 3
non-small cell lung cancer/Phase 3
non-squamous non-small cell lung cancer/Phase 2
oral cancer/Phase 1
oral squamous cell carcinoma/Phase 1
oral squamous cell carcinoma/Phase 2
oropharyngeal cancer/Phase 2
oropharyngeal squamous-cell

© 2000-2014 QIAGEN. All rights reserved.

carcinoma/Phase 1
oropharyngeal squamous-cell carcinoma/Phase 1/Phase 2
oropharyngeal squamous-cell carcinoma/Phase 2
oropharyngeal squamous-cell carcinoma/Phase 3
oropharyngeal squamous-cell carcinoma/Phase 4
ovarian cancer/Phase 2
pancreatic adenocarcinoma/Phase 2
pancreatic cancer/Phase 1
pancreatic cancer/Phase 1/Phase 2
pancreatic cancer/Phase 2
pancreatic cancer/Phase 3
pancreatic cancer/Unspecified phase
peritoneal cavity cancer/Phase 2
peritoneal tumor/Phase 2
pharyngeal cancer/Phase 1
pharyngeal tumor/Phase 2
precancerous condition/Phase 2
precancerous condition/Unspecified phase
primary glioblastoma/Phase 1/Phase 2
primary head and neck squamous cell carcinoma/Phase 1
primary laryngeal squamous cell carcinoma/Phase 1
primary oral squamous cell carcinoma/Phase 1
primary peritoneal cancer/Phase 2
prostate cancer/Phase 2
prostatic tumor/Phase 2
radiation toxicity/Phase 3
RAS mutation negative colon adenocarcinoma/Phase 2
RAS mutation negative colorectal adenocarcinoma/Phase 2
RAS mutation negative colorectal cancer/Phase 2
RAS mutation negative rectal adenocarcinoma/Phase 2
rectal adenocarcinoma/Phase 1
rectal adenocarcinoma/Phase 1/Phase 2
rectal adenocarcinoma/Phase 2
rectum cancer/Phase 0
rectum cancer/Phase 1
rectum cancer/Phase 1/Phase 2
rectum cancer/Phase 2
rectum cancer/Phase 3
rectum carcinoma/Phase 1
recurrent cancer/Phase 1
recurrent cervical carcinoma/Phase 2
recurrent laryngeal squamous cell carcinoma/Phase 1
recurrent laryngeal squamous cell carcinoma/Phase 1/Phase 2
recurrent laryngeal squamous cell carcinoma/Phase 2
recurrent loco-regionally advanced squamous cell carcinoma of the head and neck/Approved
recurrent oropharyngeal squamous cell carcinoma/Phase 1
recurrent oropharyngeal squamous cell carcinoma/Phase 1/Phase 2
recurrent oropharyngeal squamous cell carcinoma/Phase 2
recurrent rectal cancer/Phase 0
recurrent rectal cancer/Phase 1
recurrent rectal cancer/Phase 1/Phase 2
recurrent rectal cancer/Phase 2
recurrent rectal cancer/Phase 3
renal cancer/Phase 1
renal parenchyma cancer/Phase 1/Phase 2
salivary gland cancer/Phase 1
salivary gland cancer/Phase 1/Phase 2
salivary gland cancer/Phase 2
salivary gland carcinoma/Phase 1
salivary gland carcinoma/Phase 1/Phase 2
salivary gland carcinoma/Phase 2
sarcoma/Phase 1
sarcoma/Phase 2
signet ring adenocarcinoma of the colon/Phase 0
signet ring adenocarcinoma of the colon/Phase 1
signet ring adenocarcinoma of the colon/Phase 2
signet ring adenocarcinoma of the rectum/Phase 0
signet ring adenocarcinoma of the rectum/Phase 1
skin cancer/Unspecified phase
skin squamous cell carcinoma/Unspecified phase
small intestine cancer/Phase 1
solid tumor/Phase 1

© 2000-2014 QIAGEN. All rights reserved.

solid tumor/Phase 1/Phase 2
squamous cell carcinoma of nasopharynx/Phase 1
squamous cell carcinoma of nasopharynx/Phase 1/Phase 2
squamous cell carcinoma of nasopharynx/Phase 2
squamous cell carcinoma of the oral cavity/Phase 1/Phase 2
squamous cell carcinoma of the oral cavity/Phase 2
squamous cell carcinoma of the oral cavity/Phase 3
squamous cell carcinoma of the oral cavity/Phase 4
squamous cell carcinoma/Phase 1
squamous cell carcinoma/Phase 1/Phase 2
squamous cell carcinoma/Phase 2
squamous cell lung cancer/Phase 1
squamous cell lung cancer/Phase 2
stage IB2 cervical carcinoma/Phase 2
stage III head and neck squamous cell carcinoma/Phase 3
stage III oropharyngeal squamous cell carcinoma/Phase 1
stage III oropharyngeal squamous cell carcinoma/Phase 1/Phase 2
stage III oropharyngeal squamous cell carcinoma/Phase 2
stage III oropharyngeal squamous cell carcinoma/Phase 2/Phase 3
stage III oropharyngeal squamous cell carcinoma/Phase 3
stage III oropharyngeal squamous cell carcinoma/Phase 4
stage III oropharyngeal squamous cell carcinoma/Unspecified phase
stage IV gastric cancer/Phase 1
stage IV head and neck squamous cell carcinoma/Phase 2
stage IV head and neck squamous cell carcinoma/Phase 3
stage IV oropharyngeal squamous cell carcinoma/Phase 1
stage IV oropharyngeal squamous cell carcinoma/Phase 1/Phase 2
stage IV oropharyngeal squamous cell carcinoma/Phase 2
stage IV oropharyngeal squamous cell carcinoma/Phase 4
stage IVA oropharyngeal squamous cell carcinoma/Phase 1
stage IVA oropharyngeal squamous cell carcinoma/Phase 1/Phase 2
stage IVA oropharyngeal squamous cell carcinoma/Phase 2
stage IVA oropharyngeal squamous cell carcinoma/Phase 2/Phase 3
stage IVA oropharyngeal squamous cell carcinoma/Phase 3
stage IVA oropharyngeal squamous cell carcinoma/Unspecified phase
stage IVB cervical carcinoma/Phase 2
stage IVB oropharyngeal squamous cell carcinoma/Phase 1
stage IVB oropharyngeal squamous cell carcinoma/Phase 1/Phase 2
stage IVB oropharyngeal squamous cell carcinoma/Phase 2
stage IVB oropharyngeal squamous cell carcinoma/Phase 2/Phase 3
stage IVB oropharyngeal squamous cell carcinoma/Unspecified phase
stage IVC oropharyngeal squamous cell carcinoma/Phase 1
stage IVC oropharyngeal squamous cell carcinoma/Phase 1/Phase 2
stage IVC oropharyngeal squamous cell carcinoma/Phase 2
stage IVC oropharyngeal squamous cell carcinoma/Phase 3
thymic carcinoma/Phase 2
thymoma/Phase 2
tongue cancer/Phase 1
tongue cancer/Phase 1/Phase 2
tongue cancer/Phase 2
tongue cancer/Phase 2/Phase 3
tongue cancer/Unspecified phase
triple negative basal like breast carcinoma/Phase 1/Phase 2
tumor/Phase 1
undifferentiated nasopharyngeal carcinoma/Phase 1
undifferentiated nasopharyngeal carcinoma/Phase 1/Phase 2

© 2000-2014 QIAGEN. All rights reserved.
INGENUITY® SYSTEMS

Drug Name	Targets	Actions	Brand Names	Indications/Status
cetuximab/irinotecan [cetuximab]	EGFR	inhibitor	Erbitux with irinotecan, Erbitux/irinotecan	KRAS mutation negative adenocarcinoma of the colon/Phase 3<br>KRAS mutation negative adenocarcinoma of the rectum/Phase 3<br>KRAS wild-type positive colorectal cancer/Phase 3<br>metastatic malignant colorectal neoplasm/Approved
cetuximab/irinotecan [cetuximab]	EGFR	antibody	Erbitux with irinotecan, Erbitux/irinotecan	KRAS mutation negative adenocarcinoma of the colon/Phase 3<br>KRAS mutation negative adenocarcinoma of the rectum/Phase 3<br>KRAS wild-type positive colorectal cancer/Phase 3<br>metastatic malignant colorectal neoplasm/Approved
cetuximab/irinotecan [cetuximab]	EGFR	antagonist	Erbitux with irinotecan, Erbitux/irinotecan	KRAS mutation negative adenocarcinoma of the colon/Phase 3<br>KRAS mutation negative adenocarcinoma of the rectum/Phase 3<br>KRAS wild-type positive colorectal cancer/Phase 3<br>metastatic malignant colorectal neoplasm/Approved
chlorambucil/fludarabine phosphate [fludarabine phosphate]	RRM2	inhibitor		

© 2000-2014 QIAGEN. All rights reserved.

Drug Name	Targets	Actions	Brand Names	Indications/Status
cladribine	RRM2	inhibitor	Cladribine Novaplus, Leustatin, Mylinax	acute myeloid leukemia/Phase 1<br>acute myeloid leukemia/Phase 1/Phase 2<br>acute myeloid leukemia/Phase 2<br>acute myeloid leukemia/Phase 3<br>adult acute lymphocytic leukemia/Phase 4<br>adult acute myeloid leukemia with 11q23 abnormalities/Phase 1/Phase 2<br>adult acute myeloid leukemia/Phase 1/Phase 2<br>CD20-positive malignant lymphocytic neoplasm/Phase 2<br>central nervous system tumor/Phase 1<br>chronic B-cell leukemia/Phase 2<br>chronic lymphocytic leukemia/Phase 1/Phase 2<br>chronic lymphocytic leukemia/Phase 2<br>chronic lymphocytic leukemia/Phase 3<br>chronic myeloproliferative disorder/Phase 1<br>follicular non-Hodgkin's disease/Phase 2<br>graft-vs-host disease/Phase 1<br>graft-vs-host disease/Phase 2<br>hairy-cell leukemia/Approved<br>hairy-cell leukemia/Phase 2<br>Langerhans cell histiocytosis of lung/Phase 2<br>Langerhans cell histiocytosis/Phase 2/Phase 3<br>leukemia/Phase 1<br>leukemia/Phase 2<br>leukemia/Phase 3<br>lymphoblastic lymphoma/Phase 2<br>lymphoblastic lymphoma/Phase 4<br>lymphocytic cancer/Phase 1<br>lymphocytic cancer/Phase 1/Phase 2<br>lymphocytic cancer/Phase 2<br>lymphocytic cancer/Unspecified phase<br>lymphoplasmacytoid lymphoma/Phase 2<br>mantle cell lymphoma/Phase 1/Phase 2<br>mantle cell lymphoma/Phase 2<br>marginal zone cell lymphoma/Phase 2<br>multiple myeloma/Phase 1<br>multiple sclerosis/Phase 1<br>multiple sclerosis/Phase 2<br>multiple sclerosis/Phase 3<br>myelodysplastic syndrome/Phase 1<br>myelodysplastic syndrome/Phase 1/Phase 2<br>myelodysplastic syndrome/Phase 2<br>myeloid leukemia/Phase 2/Phase 3<br>primary sclerosing cholangitis/Phase 2<br>recurrent adult acute myeloid leukemia/Phase 1/Phase 2<br>refractory B-cell non-Hodgkin lymphoma/Phase 2<br>relapsed B-cell non-Hodgkin lymphoma/Phase 2<br>relapsing-remitting multiple sclerosis/Phase 3<br>secondary acute myeloid leukemia/Phase 1/Phase 2<br>small intestine cancer/Phase 1<br>Waldenstrom's macroglobulinemia/Phase 2
cladribine/cytarabine/daunorubicin [cladribine]	RRM2	inhibitor		
cladribine/cytarabine/decitabine [cladribine]	RRM2	inhibitor		
cladribine/cytarabine/filgrastim [cladribine]	RRM2	inhibitor		
cladribine/cytarabine/filgrastim/idarubicin [cladribine]	RRM2	inhibitor		
cladribine/cytarabine/filgrastim/idarubicin/plerixafor [cladribine]	RRM2	inhibitor		
cladribine/cytarabine/filgrastim/mitoxantrone [cladribine]	RRM2	inhibitor		
cladribine/peginterferon alfa-2a [cladribine]	RRM2	inhibitor		c-KIT D816V mutation positive systemic mastocytosis/Phase 2/Phase 3<br>c-KIT exon 17 mutation positive systemic mastocytosis/Phase 2/Phase 3<br>systemic mastocytosis/Phase 2/Phase 3
cladribine/rituximab [cladribine]	RRM2	inhibitor		

© 2000-2014 QIAGEN. All rights reserved.
INGENUITY SYSTEMS

Drug Name	Targets	Actions	Brand Names	Indications/Status
clotrimazole	CYP51A1	antagonist	Canastene, Canesten, Canesten 1-Day Cream Combi-Pak, Canesten 1-Day Therapy, Canesten 3-Day Therapy, Canesten 6-Day Therapy, Canesten Combi-Pak 1-Day Therapy, Canesten Combi-Pak 3-Day Therapy, Canesten Cream, Canesten Solution, Canestine, Canifug, Cimitidine, Clomaz, Clomine, Clotrimaderm, Empecid, FemCare, Fungicide, Gyne-Lotrimin, Gyne-Lotrimin 3, Gyne-Lotrimin 3 Combination Pack, Gyne-Lotrimin Combination Pack, Gynix, Lotrimin, Lotrimin Af, Lotrimin AF Cream, Lotrimin AF Jock-Itch Cream, Lotrimin AF Lotion, Lotrimin AF Solution, Lotrimin Cream, Lotrimin Lotion, Lotrimin Solution, Mono-baycuten, Mycelax, Mycelex, Mycelex 7, Mycelex Cream, Mycelex Solution, Mycelex Troches, Mycelex Twin Pack, Mycelex-7, Mycelex-7 Combination Pack, Mycelex-G, Myclo Cream, Myclo Solution, Myclo Spray Solution, Myclo-Gyne, Mycosporin, Mykosporin, Neo-Zol Cream, Trimysten, Trivagizole 3, Veltrim	candidiasis/Approved<br>candidiasis/Phase 3<br>Crohn's disease/Phase 1/Phase 2<br>folliculitis/Phase 2/Phase 3<br>infection/Phase 3<br>infection/Unspecified phase<br>inflammatory bowel disease/Phase 1/Phase 2<br>insulin-dependent diabetes mellitus/Unspecified phase<br>kidney disease/Phase 1/Phase 2<br>mycosis/Phase 3<br>oral candidiasis/Phase 3<br>oral candidiasis/Unspecified phase<br>sickle cell anemia/Phase 1/Phase 2<br>sickle cell anemia/Unspecified phase<br>tinea corporis/Approved<br>tinea corporis/Phase 3<br>tinea cruris/Approved<br>tinea cruris/Phase 3<br>tinea pedis/Approved<br>tinea pedis/Phase 3<br>ulcerative colitis/Phase 1/Phase 2<br>vaginitis/Phase 3
cyclophosphamide/fludarabine phosphate/mitoxantrone [fludarabine phosphate]	RRM2	inhibitor		
cyclophosphamide/fludarabine phosphate/mitoxantrone/rituximab [fludarabine phosphate]	RRM2	inhibitor		
cyclophosphamide/fludarabine phosphate/rituximab [fludarabine phosphate]	RRM2	inhibitor	Rituxin/cyclophosphamide/fludarabine phosphate	CD20 positive chronic lymphocytic leukemia/Approved<br>chronic lymphocytic leukemia/Phase 1<br>chronic lymphocytic leukemia/Phase 1/Phase 2<br>chronic lymphocytic leukemia/Phase 2
cytarabine/filgrastim/fludarabine phosphate [fludarabine phosphate]	RRM2	inhibitor		
cytarabine/fludarabine phosphate [fludarabine phosphate]	RRM2	inhibitor		
cytarabine/fludarabine phosphate/oxaliplatin/rituximab [fludarabine phosphate]	RRM2	inhibitor		
dexamethasone/fludarabine phosphate/mitoxantrone [fludarabine phosphate]	RRM2	inhibitor		
dexamethasone/fludarabine phosphate/mitoxantrone/rituximab [fludarabine phosphate]	RRM2	inhibitor		
docetaxel/gemcitabine [gemcitabine]	RRM2	inhibitor		esophageal squamous cell carcinoma/Phase 2<br>lung tumor/Phase 3<br>metastatic esophageal squamous cell carcinoma/Phase 2
docetaxel/gemcitabine/vincristine [gemcitabine]	RRM2	inhibitor		
econazole	CYP51A1	antagonist	Econazole Nitrate, Ecostatin, Ecostatin cream, Ecostatin Vaginal Ovules, Gyno-Pevaryl, Gyno-Pevaryl 150, Ifenec, Palavale, Pevaryl, Spectazole, Spectazole cream	candidiasis/Approved<br>tinea corporis/Approved<br>tinea cruris/Approved<br>tinea pedis/Approved<br>tinea pedis/Phase 2<br>tinea pedis/Phase 3
EGF816	EGFR	inhibitor		c-MET positive EGFR mutation negative non-small cell lung cancer/Phase 2<br>EGFR exon 19 deletion positive EGFR L858R mutation positive non-small cell lung cancer/Phase 1/Phase 2<br>EGFR exon 19 deletion positive non-small cell lung cancer/Phase 1/Phase 2<br>EGFR L858R mutation positive non-small cell lung cancer/Phase 1/Phase 2<br>EGFR mutation positive non-small cell lung cancer/Phase 1/Phase 2<br>EGFR mutation positive solid tumor/Phase 1/Phase 2<br>EGFR T790M mutation positive lung adenocarcinoma/Phase 2<br>EGFR T790M positive non-small cell lung cancer/Phase 1/Phase 2<br>EGFR T790M positive non-small cell lung cancer/Phase 2
EGFR antisense DNA	EGFR	binder		head and neck cancer/Phase 1<br>head and neck cancer/Phase 1/Phase 2<br>head and neck squamous cell carcinoma/Phase 1/Phase 2
EGFR tyrosine kinase inhibitor	EGFR	inhibitor		EGFR exon 19 mutation positive non-small cell lung cancer/Phase 2<br>EGFR exon 21 mutation positive non-small cell lung cancer/Phase 2<br>EGFR mutation positive non-small cell lung cancer/Unspecified phase<br>metastatic non-small-cell lung cancer/Phase 2

© 2000-2014 QIAGEN. All rights reserved.

Drug Name	Targets	Actions	Brand Names	Indications/Status
enzastaurin	GSK3B	inhibitor		adult solid tumor/Phase 1<br>B-cell non-Hodgkin's disease/Phase 2<br>breast cancer/Phase 2<br>cancer/Phase 2<br>carcinoma/Phase 2<br>central nervous system tumor/Phase 1<br>colorectal cancer/Phase 2<br>colorectal carcinoma/Phase 2<br>colorectal tumor/Phase 2<br>cutaneous T-cell lymphoma/Phase 2<br>fallopian tube neoplasm/Phase 2<br>follicular non-Hodgkin's disease/Phase 2<br>glioblastoma cancer/Phase 1/Phase 2<br>glioblastoma cancer/Phase 2<br>glioblastoma cancer/Phase 3<br>glioblastoma/Phase 1/Phase 2<br>glioblastoma/Phase 2<br>glioma cancer/Phase 1<br>glioma/Phase 1<br>gliosarcoma cancer/Phase 1/Phase 2<br>large-cell diffuse lymphoma/Phase 2<br>lung cancer/Phase 2<br>lymphocytic cancer/Phase 1<br>lymphocytic cancer/Phase 2<br>lymphocytic leukemia/Phase 1<br>malignant glioma/Phase 2<br>mammary tumor/Phase 2<br>mantle cell lymphoma/Phase 2<br>metastatic renal cell carcinoma/Phase 2<br>multiple myeloma/Phase 2<br>neuroblastoma/Phase 1<br>non-Hodgkin's disease/Phase 2<br>non-Hodgkin's disease/Phase 3<br>non-small cell lung cancer/Phase 1/Phase 2<br>non-small cell lung cancer/Phase 2<br>ovarian cancer/Phase 2<br>pancreatic neoplasia/Phase 2<br>peritoneal tumor/Phase 2<br>primary peritoneal cancer/Phase 2<br>prostate cancer/Phase 2<br>small cell lung cancer/Phase 2<br>solid tumor/Phase 1<br>T-cell non-Hodgkin's disease/Phase 2<br>tumor/Phase 2<br>Waldenstrom's macroglobulinemia/Phase 2
erlotinib	EGFR	inhibitor	Tarceva	adenocarcinoma/Phase 2<br>adenomatous polyp/Phase 2<br>adenomatous polyposis coli/Phase 2<br>adenosquamous carcinoma/Phase 3<br>adenosquamous lung carcinoma/Phase 2<br>adrenal gland tumor/Phase 2<br>adult solid tumor/Phase 1<br>adult solid tumor/Phase 1/Phase 2<br>advanced cancer/Phase 1<br>advanced non-small-cell lung cancer/Phase 2<br>advanced non-small-cell lung cancer/Phase 3<br>advanced solid tumor/Phase 1<br>advanced solid tumor/Phase 2<br>advanced solid tumor/Unspecified phase<br>ALK fusion negative EGFR mutation negative non-small cell lung adenocarcinoma/Phase 3<br>ALK fusion negative EGFR T790M mutation negative KRAS mutation negative non-small cell lung cancer/Phase 1<br>ALK fusion negative EGFR TK activating negative non-small cell lung cancer/Phase 3<br>ALK fusion negative EGFR-activating mutation negative non-small cell lung cancer/Phase 2<br>ALK fusion positive EGFR exon 19 deletion positive EGFR T790M mutation negative non-small cell lung carcinoma/Phase 3<br>ALK fusion positive EGFR L858R mutation positive EGFR T790M mutation negative non-small cell lung carcinoma/Phase 3<br>ALK fusion positive non-small cell lung cancer/Phase 2<br>ALK mutation positive non-small cell lung cancer/Phase 1<br>ALK mutation positive non-small cell lung cancer/Phase 1/Phase 2<br>ALK-EML4 fusion negative non-small cell lung cancer/Phase 2

© 2000-2014 QIAGEN. All rights reserved.
INGENUITY® SYSTEMS

ALK-EML4 fusion positive EGFR T790M mutation negative non-small cell lung cancer/Phase 2
anal cancer/Phase 1
anaplastic astrocytoma/Phase 1/Phase 2
anaplastic oligoastrocytoma/Phase 1/Phase 2
anaplastic oligodendroglioma/Phase 1/Phase 2
appendix carcinoma/Phase 1
basal-cell carcinoma/Phase 2
bile duct cancer/Phase 2
bladder cancer/Phase 1
bladder cancer/Phase 2
bladder carcinoma/Phase 2
BRAF activating mutation positive malignant solid tumor/Phase 2
BRAF mutation negative EGFR mutation positive KRAS mutation negative solid tumor/Phase 1
BRAF mutation negative KRAS mutation negative PTEN loss solid tumor/Phase 1
brain cancer/Phase 2
brain cancer/Unspecified phase
brain metastasis associated with EGFR mutation positive non-small cell lung cancer/Phase 2
brain metastasis associated with EGFR mutation positive non-small cell lung cancer/Phase 3
brain metastasis associated with lung adenocarcinoma/Phase 2
brain metastasis associated with non-small cell lung cancer/Phase 3
brain metastasis/Phase 1
brain metastasis/Phase 2
brainstem glioma/Phase 1
breast cancer/Phase 1
breast cancer/Phase 1/Phase 2
breast cancer/Phase 2
breast cancer/Unspecified phase
breast carcinoma/Phase 2
brenner tumor/Phase 2
bronchiolo-alveolar adenocarcinoma/Phase 2
c-MET amplified solid tumor/Phase 1
c-MET positive non-small cell lung cancer/Phase 1
c-MET positive non-small cell lung cancer/Phase 2
c-MET positive non-small cell lung cancer/Phase 3
c-MET positive non-small cell lung carcinoma/Phase 2
cancer/Phase 1
cancer/Phase 2
cancer/Unspecified phase
carcinoid tumor/Phase 2
carcinoma/Phase 2
castration refractory prostate cancer/Phase 1
central nervous system tumor/Phase 1
central nervous system tumor/Phase 1/Phase 2
central nervous system tumor/Phase 2
cervical cancer/Phase 1
cervical cancer/Phase 2
cervical squamous cell carcinoma/Phase 2
childhood brain tumor/Phase 1
childhood brain tumor/Unspecified phase
childhood cerebellar astrocytoma/Phase 1
childhood cerebral astrocytoma/Phase 1
childhood medulloblastoma/Phase 1
childhood rhabdomyosarcoma/Phase 1
childhood supratentorial primitive neuroectodermal tumor/Phase 1
cholangiocarcinoma/Phase 1
cholangiocarcinoma/Phase 2
clear cell ovarian cystadenocarcinoma/Phase 2
colon adenocarcinoma/Phase 1
colon adenocarcinoma/Phase 2
colon cancer/Phase 1
colon cancer/Phase 1/Phase 2
colon cancer/Phase 2
colon tumor/Phase 1
colorectal cancer/Phase 1
colorectal cancer/Phase 1/Phase 2
colorectal cancer/Phase 2
colorectal cancer/Phase 3
diffuse intrinsic pontine glioma/Phase 2
dysplasia/Phase 1
Ebola hemorrhagic fever/Phase

© 2000-2014 QIAGEN. All rights reserved.

1/Phase 2
EGFR (L858R) positive lung adenocarcinoma/Phase 2
EGFR (L858R) positive lung adenocarcinoma/Phase 3
EGFR activating mutation positive adenocarcinoma of the lung/Phase 1/Phase 2
EGFR activating mutation positive adenocarcinoma of the lung/Phase 4
EGFR activating mutation positive EGFR T790M mutation negative non-small cell lung cancer/Phase 3
EGFR activating mutation positive EGFR T790M mutation negative nonsquamous non-small cell lung cancer/Phase 2
EGFR activating mutation positive malignant solid tumor/Phase 2
EGFR activating mutation positive non-squamous non-small cell lung cancer/Phase 2
EGFR E746-A750del mutation positive cancer/Phase 1
EGFR E746-A750del mutation positive solid tumor/Phase 1
EGFR exon 19 deletion positive EGFR T790M mutation negative non-small cell lung carcinoma/Phase 3
EGFR exon 19 deletion positive L858R mutation positive lung adenocarcinoma/Phase 2
EGFR exon 19 deletion positive lung adenocarcinoma/Phase 2
EGFR exon 19 deletion positive lung adenocarcinoma/Phase 3
EGFR exon 19 deletion positive non-small cell lung cancer/Phase 1
EGFR exon 19 deletion positive non-small cell lung cancer/Phase 2
EGFR exon 19 deletion positive non-small cell lung cancer/Phase 3
EGFR exon 19 deletion positive nonsquamous non-small cell lung carcinoma/Phase 2
EGFR exon 19 mutation negative non-small cell lung cancer/Phase 3
EGFR exon 19 mutation positive KRAS mutation negative non-small cell lung cancer/Phase 2
EGFR exon 19 mutation positive non-small cell lung cancer/Phase 2
EGFR exon 19 mutation positive non-small cell lung cancer/Phase 2/Phase 3
EGFR exon 19 mutation positive non-small cell lung cancer/Phase 3
EGFR exon 20 mutation positive cancer/Phase 2
EGFR exon 21 mutation negative non-small cell lung cancer/Phase 3
EGFR exon 21 mutation positive KRAS mutation negative non-small cell lung cancer/Phase 2
EGFR exon 21 mutation positive non-small cell lung cancer/Phase 2
EGFR exon 21 mutation positive non-small cell lung cancer/Phase 2/Phase 3
EGFR exon 21 mutation positive non-small cell lung cancer/Phase 3
EGFR exon 21 mutation positive pancreatic cancer/Phase 2
EGFR G719A mutation positive non-small cell lung cancer/Phase 2
EGFR G719C mutation positive cancer/Phase 1
EGFR G719C mutation positive non-small cell lung cancer/Phase 2
EGFR G719C mutation positive solid tumor/Phase 1
EGFR G719S mutation positive non-small cell lung cancer/Phase 2
EGFR G719X mutation positive lung adenocarcinoma/Phase 2
EGFR G719X mutation positive non-small cell lung cancer/Phase 2
EGFR L858 mutation positive EGFR T790M mutation negative EML4-ALK fusion positive non-small cell lung cancer/Phase 2
EGFR L858 mutation positive EGFR T790M mutation negative non-small cell lung cancer/Phase 2
EGFR L858R mutation positive cancer/Phase 1
EGFR L858R mutation positive EGFR T790M mutation negative non-small cell lung carcinoma/Phase 3
EGFR L858R mutation positive non-small cell lung cancer/Phase 1
EGFR L858R mutation positive non-small cell lung cancer/Phase 2
EGFR L858R mutation positive

© 2000-2014 QIAGEN. All rights reserved.

non-small cell lung cancer/Phase 3
EGFR L858R mutation positive nonsquamous non small cell lung carcinoma/Phase 2
EGFR L858R mutation positive solid tumor/Phase 1
EGFR L861Q mutation positive lung adenocarcinoma/Phase 2
EGFR L861Q mutation positive non-small cell lung cancer/Phase 2
EGFR mutated c-MET-amplified non-small cell lung cancer/Phase 1
EGFR mutation negative non-small cell lung cancer/Phase 1/Phase 2
EGFR mutation negative non-small cell lung cancer/Phase 2
EGFR mutation positive adenocarcinoma of the lung/Phase 2
EGFR mutation positive EGFR exon 20 insertion activating mutation negative non-small cell lung cancer/Phase 2
EGFR mutation positive non small cell lung carcinoma/Phase 1
EGFR mutation positive non small cell lung carcinoma/Phase 2
EGFR mutation positive non-small cell lung cancer/Phase 1
EGFR mutation positive non-small cell lung cancer/Phase 1/Phase 2
EGFR mutation positive non-small cell lung cancer/Phase 2
EGFR mutation positive non-small cell lung cancer/Phase 3
EGFR resistant mutation positive cancer/Phase 1
EGFR sensitizing mutation positive cancer/Phase 1
EGFR sensitizing mutation positive non-small cell lung cancer/Phase 1
EGFR sensitizing mutation positive non-small cell lung cancer/Phase 1/Phase 2
EGFR sensitizing mutation positive non-small cell lung cancer/Phase 2
EGFR sensitizing mutation positive non-small cell lung cancer/Phase 3
EGFR sensitizing mutation positive non-small cell lung carcinoma/Phase 1/Phase 2
EGFR sensitizing mutation positive non-squamous non-small cell lung cancer/Phase 2
EGFR sensitizing mutation positive solid tumor/Phase 1
EGFR T790M mutation positive cancer/Phase 1
EGFR T790M mutation positive solid tumor/Phase 1
EGFR TK domain mutation positive malignant peritoneal mesothelioma/Phase 2
EGFR(L858R) positive metastatic non-small cell lung cancer/Approved
endometrial cancer/Phase 2
ependymoma/Phase 2
epithelial ovarian cancer/Phase 1
epithelial ovarian cancer/Phase 1/Phase 2
epithelial ovarian cancer/Phase 2
epithelioid mesothelioma/Phase 2
esophageal adenocarcinoma/Phase 1
esophageal adenocarcinoma/Phase 2
esophageal cancer/Phase 1
esophageal cancer/Phase 2
esophageal cancer/Phase 3
esophageal squamous cell cancer/Phase 3
esophageal squamous cell carcinoma/Phase 1
esophageal squamous cell carcinoma/Phase 2
estrogen receptor positive progesterone receptor positive cancer/Phase 1
estrogen receptor positive progesterone receptor positive solid malignant tumor/Phase 1
estrogen receptor-negative breast cancer/Phase 2
extrahepatic bile duct cancer/Phase 1
extrahepatic bile duct cancer/Phase 2
extrahepatic cholangiocarcinoma/Phase 2
fallopian tube cancer/Phase 1/Phase 2
fallopian tube cancer/Phase 2
fallopian tube cancer/Phase 3
gall bladder adenocarcinoma/Phase 2
gall bladder carcinoma/Phase 2

© 2000-2014 QIAGEN. All rights reserved.

gallbladder cancer/Phase 1
gallbladder cancer/Phase 2
gastric adenocarcinoma/Phase 1
gastric cancer/Phase 1
gastric cancer/Phase 2
gastrointestinal stromal tumor/Phase 1
gastrointestinal tract cancer/Phase 2
gastrointestinal tract cancer/Unspecified phase
glioblastoma cancer/Phase 1
glioblastoma cancer/Phase 2
glioblastoma/Phase 1/Phase 2
glioblastoma/Phase 2
glioma cancer/Unspecified phase
gliosarcoma cancer/Phase 1
gliosarcoma cancer/Phase 1/Phase 2
gliosarcoma cancer/Phase 2
head and neck cancer/Phase 0
head and neck cancer/Phase 1
head and neck cancer/Phase 1/Phase 2
head and neck cancer/Phase 2
head and neck cancer/Phase 2/Phase 3
head and neck cancer/Phase 3
head and neck cancer/Unspecified phase
head and neck squamous cell carcinoma/Phase 1/Phase 2
head and neck squamous cell carcinoma/Phase 2
head and neck tumor/Unspecified phase
hematologic cancer/Phase 2
hepatocellular carcinoma/Phase 1
hepatocellular carcinoma/Phase 1/Phase 2
hepatocellular carcinoma/Phase 2
HER2 activating mutation positive breast cancer/Phase 2
HER2 activating mutation positive gastric cancer/Phase 2
HER2 activating mutation positive gastroesophageal junction cancer/Phase 2
HER2 negative hormone receptor negative breast cancer/Phase 1
HER2 positive solid malignant tumor/Phase 2
HER2-negative breast cancer/Phase 2
HER3 positive carcinoma/Phase 1
hereditary leiomyomatosis and renal cell cancer/Phase 2
hormone receptor positive solid tumor/Phase 1
hypopharyngeal squamous cell carcinoma/Phase 1
hypopharyngeal squamous cell carcinoma/Phase 1/Phase 2
hypopharyngeal squamous cell carcinoma/Phase 2
islet cell tumor/Phase 2
Klatskin's tumor/Phase 2
KRAS codon 12 mutation positive non-small cell lung cancer/Phase 3
KRAS codon 13 mutation positive non-small cell lung cancer/Phase 3
KRAS mutation negative colorectal carcinoma/Phase 1
KRAS mutation negative non-small cell lung cancer/Phase 2
KRAS mutation positive non small cell lung cancer/Phase 1
KRAS mutation positive non small cell lung cancer/Phase 2
KRAS wild-type positive colorectal cancer/Phase 2
large cell lung cancer/Phase 1
large cell lung cancer/Phase 2
large cell lung carcinoma/Phase 2
large-cell carcinoma/Phase 3
laryngeal squamous cell carcinoma/Phase 1
laryngeal squamous cell carcinoma/Phase 1/Phase 2
laryngeal squamous cell carcinoma/Phase 2
laryngeal verrucous carcinoma/Phase 1
laryngeal verrucous carcinoma/Phase 1/Phase 2
laryngeal verrucous carcinoma/Phase 2
leukemia/Phase 2
liver cancer/Phase 1
liver cancer/Phase 1/Phase 2
liver cancer/Phase 2
local neoplasm recurrence/Phase 2
locally advanced EGFR mutation positive lung adenocarcinoma/Phase 2
locally advanced KRAS mutation positive non small cell lung

© 2000-2014 QIAGEN. All rights reserved.

cancer/Phase 2
locally advanced non small cell lung cancer/Approved
locally advanced non small cell lung cancer/Phase 2
locally advanced pancreatic cancer/Phase 2
low-grade glioma/Phase 1
lung adenocarcinoma/Phase 1/Phase 2
lung adenocarcinoma/Phase 2
lung adenocarcinoma/Phase 4
lung adenosquamous cancer/Phase 2
lung cancer/Phase 1
lung cancer/Phase 1/Phase 2
lung cancer/Phase 2
lung cancer/Phase 3
lung cancer/Unspecified phase
lung squamous cell carcinoma/Phase 2
lung tumor/Phase 2
lymphocytic cancer/Phase 1
lymphoepithelial carcinoma/Phase 1/Phase 2
lymphoma/Phase 1
male breast cancer/Phase 1
malignant brain neoplasm/Phase 1
malignant glioma/Phase 1/Phase 2
malignant mesothelioma/Phase 1
malignant mesothelioma/Phase 2
malignant solid tumor/Phase 1
malignant thyroid gland neoplasm/Phase 1
mammary tumor/Phase 1
mammary tumor/Phase 2
medulloblastoma/Phase 2
melanoma/Phase 1
melanoma/Phase 2
mesothelioma/Phase 2
metaplasia/Phase 1
metastasis/Phase 1
metastasis/Phase 2
metastasis/Phase 3
metastatic breast cancer/Phase 1
metastatic breast cancer/Phase 2
metastatic colorectal cancer/Phase 2
metastatic colorectal cancer/Phase 3
metastatic KRAS mutation positive non-small-cell lung cancer/Phase 2
metastatic non-small-cell lung cancer/Approved
metastatic non-small-cell lung cancer/Phase 1
metastatic non-small-cell lung cancer/Phase 1/Phase 2
metastatic non-small-cell lung cancer/Phase 2
metastatic non-small-cell lung cancer/Phase 3
metastatic non-small-cell lung cancer/Phase 4
metastatic non-small-cell lung cancer/Unspecified phase
metastatic pancreatic adenocarcinoma/Phase 1
metastatic pancreatic adenocarcinoma/Phase 2
metastatic pancreatic cancer/Phase 1
metastatic pancreatic cancer/Phase 1/Phase 2
metastatic pancreatic cancer/Phase 2
metastatic papillary renal cancer/Phase 2
metastatic solid tumor/Phase 1
metastatic uveal melanoma/Phase 1
mucinous adenocarcinoma of colon/Phase 1
mucinous adenocarcinoma of rectum/Phase 1
multiple endocrine neoplasia/Phase 2
multiple myeloma/Phase 1
muscle invasive bladder cancer/Phase 2
myelodysplastic syndrome/Phase 1/Phase 2
myelodysplastic syndrome/Phase 2
nasopharyngeal cancer/Phase 2
neoplasia/Phase 1
neuroblastoma/Phase 1
neuroblastoma/Phase 2
neuroendocrine tumor/Phase 1
neuroendocrine tumor/Phase 2
non small cell lung adenocarcinoma/Phase 1
non small cell lung adenocarcinoma/Phase 1/Phase 2
non small cell lung adenocarcinoma/Phase 2
non-small cell lung cancer with activating EGFR mutations/Phase 2

© 2000-2014 QIAGEN. All rights reserved.

non-small cell lung cancer with activating EGFR mutations/Phase 3
non-small cell lung cancer/Phase 0
non-small cell lung cancer/Phase 1
non-small cell lung cancer/Phase 1/Phase 2
non-small cell lung cancer/Phase 2
non-small cell lung cancer/Phase 3
non-small cell lung cancer/Phase 4
non-small cell lung cancer/Unspecified phase
non-small-cell lung carcinoma/Approved
non-squamous non-small cell lung cancer/Phase 2
non-squamous non-small cell lung cancer/Phase 3
occult primary tumor/Phase 2
oral cancer/Phase 3
oropharyngeal squamous-cell carcinoma/Phase 1
oropharyngeal squamous-cell carcinoma/Phase 1/Phase 2
oropharyngeal squamous-cell carcinoma/Phase 2
osteosarcoma/Phase 1
ovarian cancer/Phase 1/Phase 2
ovarian cancer/Phase 2
ovarian cancer/Phase 3
ovarian endometrioid carcinoma/Phase 2
ovarian mucinous cystadenocarcinoma/Phase 2
ovarian serous cystadenocarcinoma/Phase 2
ovarian tumor/Phase 2
pancreatic acinar cell carcinoma/Phase 1
pancreatic acinar cell carcinoma/Phase 2/Phase 3
pancreatic adenocarcinoma/Phase 1
pancreatic adenocarcinoma/Phase 1/Phase 2
pancreatic adenocarcinoma/Phase 2
pancreatic cancer/Phase 1
pancreatic cancer/Phase 1/Phase 2
pancreatic cancer/Phase 2
pancreatic cancer/Phase 2/Phase 3
pancreatic cancer/Phase 3
pancreatic carcinoma/Phase 1
pancreatic carcinoma/Phase 2
pancreatic ductal adenocarcinoma/Phase 1
pancreatic ductal adenocarcinoma/Phase 2
pancreatic ductal adenocarcinoma/Phase 2/Phase 3
pancreatic intraductal papillary mucinous tumor/Phase 2
papillary renal cancer/Phase 2
papillary renal cell carcinoma/Phase 1
papillary thyroid cancer/Phase 2
papillary type II renal carcinoma/Phase 2
parotid gland cancer/Phase 1
peritoneal cavity cancer/Phase 1/Phase 2
peritoneal cavity cancer/Phase 2
pharyngeal cancer/Phase 0
polycythemia vera/Phase 2
precancerous condition/Phase 1/Phase 2
primary head and neck squamous cell carcinoma/Phase 1
primary head and neck squamous cell carcinoma/Phase 1/Phase 2
primary laryngeal squamous cell carcinoma/Phase 1/Phase 2
primary oral squamous cell carcinoma/Phase 1/Phase 2
primary peritoneal cancer/Phase 2
primary peritoneal cancer/Phase 3
primary sclerosing cholangitis/Phase 1
progesterone receptor-negative breast cancer/Phase 2
progressive ALK fusion negative EGFR mutation negative squamous cell non-small cell lung cancer/Phase 2/Phase 3
progressive recurrent EGFR activating mutation positive lung cancer/Phase 1/Phase 2
progressive unresectable EGFR activating mutation positive lung cancer/Phase 1/Phase 2
prostate cancer/Phase 1
prostate cancer/Phase 1/Phase 2
prostate cancer/Phase 2
psoriasis/Phase 2
PTCH1 loss-of-function mutation positive malignant solid tumor/Phase 2

© 2000-2014 QIAGEN. All rights reserved.

rectal adenocarcinoma/Phase 1
rectal adenocarcinoma/Phase 1/Phase 2
rectal adenocarcinoma/Phase 2
rectum cancer/Phase 1
rectum cancer/Phase 1/Phase 2
rectum cancer/Phase 2
recurrent breast cancer/Phase 1
recurrent breast cancer/Phase 2
recurrent EGFR activating mutation positive non-squamous non-small cell lung cancer/Phase 2
recurrent EGFR exon 19 deletion positive non-small cell lung cancer/Phase 2
recurrent EGFR L858R mutation positive non-small cell lung cancer/Phase 2
recurrent laryngeal squamous cell carcinoma/Phase 1
recurrent laryngeal squamous cell carcinoma/Phase 1/Phase 2
recurrent laryngeal squamous cell carcinoma/Phase 2
recurrent prostate cancer/Phase 1
recurrent rectal cancer/Phase 1
recurrent rectal cancer/Phase 1/Phase 2
recurrent rectal cancer/Phase 2
renal cancer/Phase 1
renal cancer/Phase 2
renal clear cell adenocarcinoma/Phase 1
renal clear cell adenocarcinoma/Phase 1/Phase 2
renal parenchyma cancer/Phase 1/Phase 2
renal parenchyma cancer/Phase 2
renal parenchyma cancer/Phase 4
renal-cell carcinoma/Phase 1
renal-cell carcinoma/Phase 1/Phase 2
renal-cell carcinoma/Phase 2
ROS1 fusion positive non-small cell lung cancer/Phase 2
salivary gland cancer/Phase 1
salivary gland cancer/Phase 1/Phase 2
salivary gland cancer/Phase 2
sarcoma/Phase 2
signet ring adenocarcinoma of the colon/Phase 1
signet ring adenocarcinoma of the rectum/Phase 1
skin cancer/Phase 1/Phase 2
skin cancer/Phase 2
skin squamous cell carcinoma/Phase 1/Phase 2
skin squamous cell carcinoma/Phase 2
small cell lung cancer/Phase 2
small intestine cancer/Phase 1
SMO activating mutation positive malignant solid tumor/Phase 2
solid tumor/Phase 1
sporadic renal papillary cancer/Phase 2
squamous cell carcinoma of nasopharynx/Phase 1
squamous cell carcinoma of nasopharynx/Phase 1/Phase 2
squamous cell carcinoma of nasopharynx/Phase 2
squamous cell carcinoma of the esophagogastric junction/Phase 3
squamous cell carcinoma of the oral cavity/Phase 1/Phase 2
squamous cell carcinoma/Phase 1
squamous cell carcinoma/Phase 2
squamous cell carcinoma/Phase 2/Phase 3
squamous cell carcinoma/Phase 3
squamous cell lung cancer/Phase 1
squamous cell lung cancer/Phase 2
squamous cell lung cancer/Phase 2/Phase 3
stage 1 rectal cancer/Phase 2
stage 3a non small cell lung carcinoma/Phase 2
stage II gastric cancer/Phase 1
stage II oropharyngeal squamous cell carcinoma/Phase 1
stage III gastric cancer/Phase 1
stage III oropharyngeal squamous cell carcinoma/Phase 1
stage III oropharyngeal squamous cell carcinoma/Phase 1/Phase 2
stage III oropharyngeal squamous cell carcinoma/Phase 2
stage IV gastric cancer/Phase 1
stage IV oropharyngeal squamous cell carcinoma/Phase 1
stage IV oropharyngeal squamous cell carcinoma/Phase 1/Phase 2
stage IV oropharyngeal squamous cell carcinoma/Phase 2
stage IVA oropharyngeal

© 2000-2014 QIAGEN. All rights reserved.

				squamous cell carcinoma/Phase 1<br>stage IVA oropharyngeal squamous cell carcinoma/Phase 1/Phase 2<br>stage IVB oropharyngeal squamous cell carcinoma/Phase 1<br>stage IVB oropharyngeal squamous cell carcinoma/Phase 1/Phase 2<br>stage IVC oropharyngeal squamous cell carcinoma/Phase 1<br>stage IVC oropharyngeal squamous cell carcinoma/Phase 1/Phase 2<br>thymoma/Phase 2<br>thymus cancer/Phase 2<br>tongue cancer/Phase 1<br>tongue cancer/Phase 2<br>transitional cell epithelial cancer/Phase 2<br>tumor/Phase 1<br>type M4 acute myeloid leukemia/Phase 2<br>undifferentiated nasopharyngeal carcinoma/Phase 1<br>undifferentiated nasopharyngeal carcinoma/Phase 1/Phase 2<br>unresectable EGFR activating mutation positive MET-positive non small cell lung cancer/Phase 3
erlotinib	EGFR	antagonist	Tarceva	adenocarcinoma/Phase 2<br>adenomatous polyp/Phase 2<br>adenomatous polyposis coli/Phase 2<br>adenosquamous carcinoma/Phase 3<br>adenosquamous lung carcinoma/Phase 2<br>adrenal gland tumor/Phase 2<br>adult solid tumor/Phase 1<br>adult solid tumor/Phase 1/Phase 2<br>advanced cancer/Phase 1<br>advanced non-small-cell lung cancer/Phase 2<br>advanced non-small-cell lung cancer/Phase 3<br>advanced solid tumor/Phase 1<br>advanced solid tumor/Phase 2<br>advanced solid tumor/Unspecified phase<br>ALK fusion negative EGFR mutation negative non-small cell lung adenocarcinoma/Phase 3<br>ALK fusion negative EGFR T790M mutation negative KRAS mutation negative non-small cell lung cancer/Phase 1<br>ALK fusion negative EGFR TK activating negative non-small cell lung cancer/Phase 3<br>ALK fusion negative EGFR-activating mutation negative non-small cell lung cancer/Phase 2<br>ALK fusion positive EGFR exon 19 deletion positive EGFR T790M mutation negative non-small cell lung carcinoma/Phase 3<br>ALK fusion positive EGFR L858R mutation positive EGFR T790M mutation negative non-small cell lung carcinoma/Phase 3<br>ALK fusion positive non-small cell lung cancer/Phase 2<br>ALK mutation positive non-small cell lung cancer/Phase 1<br>ALK mutation positive non-small cell lung cancer/Phase 1/Phase 2<br>ALK-EML4 fusion negative non-small cell lung cancer/Phase 2<br>ALK-EML4 fusion positive EGFR T790M mutation negative non-small cell lung cancer/Phase 2<br>anal cancer/Phase 1<br>anaplastic astrocytoma/Phase 1/Phase 2<br>anaplastic oligoastrocytoma/Phase 1/Phase 2<br>anaplastic oligodendroglioma/Phase 1/Phase 2<br>appendix carcinoma/Phase 1<br>basal-cell carcinoma/Phase 2<br>bile duct cancer/Phase 2<br>bladder cancer/Phase 1<br>bladder cancer/Phase 2<br>bladder carcinoma/Phase 2<br>BRAF activating mutation positive malignant solid tumor/Phase 2<br>BRAF mutation negative EGFR mutation positive KRAS mutation negative solid tumor/Phase 1<br>BRAF mutation negative KRAS mutation negative PTEN loss solid tumor/Phase 1<br>brain cancer/Phase 2<br>brain cancer/Unspecified phase<br>brain metastasis associated with

© 2000-2014 QIAGEN. All rights reserved.
INGENUITY® SYSTEMS

EGFR mutation positive non-small cell lung cancer/Phase 2
brain metastasis associated with EGFR mutation positive non-small cell lung cancer/Phase 3
brain metastasis associated with lung adenocarcinoma/Phase 2
brain metastasis associated with non-small cell lung cancer/Phase 3
brain metastasis/Phase 1
brain metastasis/Phase 2
brainstem glioma/Phase 1
breast cancer/Phase 1
breast cancer/Phase 1/Phase 2
breast cancer/Phase 2
breast cancer/Unspecified phase
breast carcinoma/Phase 2
brenner tumor/Phase 2
bronchiolo-alveolar adenocarcinoma/Phase 2
c-MET amplified solid tumor/Phase 1
c-MET positive non-small cell lung cancer/Phase 1
c-MET positive non-small cell lung cancer/Phase 2
c-MET positive non-small cell lung cancer/Phase 3
c-MET positive non-small cell lung carcinoma/Phase 2
cancer/Phase 1
cancer/Phase 2
cancer/Unspecified phase
carcinoid tumor/Phase 2
carcinoma/Phase 2
castration refractory prostate cancer/Phase 1
central nervous system tumor/Phase 1
central nervous system tumor/Phase 1/Phase 2
central nervous system tumor/Phase 2
cervical cancer/Phase 1
cervical cancer/Phase 2
cervical squamous cell carcinoma/Phase 2
childhood brain tumor/Phase 1
childhood brain tumor/Unspecified phase
childhood cerebellar astrocytoma/Phase 1
childhood cerebral astrocytoma/Phase 1
childhood medulloblastoma/Phase 1
childhood rhabdomyosarcoma/Phase 1
childhood supratentorial primitive neuroectodermal tumor/Phase 1
cholangiocarcinoma/Phase 1
cholangiocarcinoma/Phase 2
clear cell ovarian cystadenocarcinoma/Phase 2
colon adenocarcinoma/Phase 1
colon adenocarcinoma/Phase 2
colon cancer/Phase 1
colon cancer/Phase 1/Phase 2
colon cancer/Phase 2
colon tumor/Phase 1
colorectal cancer/Phase 1
colorectal cancer/Phase 1/Phase 2
colorectal cancer/Phase 2
colorectal cancer/Phase 3
diffuse intrinsic pontine glioma/Phase 2
dysplasia/Phase 1
Ebola hemorrhagic fever/Phase 1/Phase 2
EGFR (L858R) positive lung adenocarcinoma/Phase 2
EGFR (L858R) positive lung adenocarcinoma/Phase 3
EGFR activating mutation positive adenocarcinoma of the lung/Phase 1/Phase 2
EGFR activating mutation positive adenocarcinoma of the lung/Phase 4
EGFR activating mutation positive EGFR T790M mutation negative non-small cell lung cancer/Phase 3
EGFR activating mutation positive EGFR T790M mutation negative nonsquamous non-small cell lung cancer/Phase 2
EGFR activating mutation positive malignant solid tumor/Phase 2
EGFR activating mutation positive non-squamous non-small cell lung cancer/Phase 2
EGFR E746-A750del mutation positive cancer/Phase 1
EGFR E746-A750del mutation positive solid tumor/Phase 1
EGFR exon 19 deletion positive

© 2000-2014 QIAGEN. All rights reserved.

EGFR T790M mutation negative
non-small cell lung
carcinoma/Phase 3
EGFR exon 19 deletion positive
L858R mutation positive lung
adenocarcinoma/Phase 2
EGFR exon 19 deletion positive
lung adenocarcinoma/Phase 2
EGFR exon 19 deletion positive
lung adenocarcinoma/Phase 3
EGFR exon 19 deletion positive
non-small cell lung cancer/Phase 1
EGFR exon 19 deletion positive
non-small cell lung cancer/Phase 2
EGFR exon 19 deletion positive
non-small cell lung cancer/Phase 3
EGFR exon 19 deletion positive
nonsquamous non-small cell lung
carcinoma/Phase 2
EGFR exon 19 mutation negative
non-small cell lung cancer/Phase 3
EGFR exon 19 mutation positive
KRAS mutation negative non-small
cell lung cancer/Phase 2
EGFR exon 19 mutation positive
non-small cell lung cancer/Phase 2
EGFR exon 19 mutation positive
non-small cell lung cancer/Phase
2/Phase 3
EGFR exon 19 mutation positive
non-small cell lung cancer/Phase 3
EGFR exon 20 mutation positive
cancer/Phase 2
EGFR exon 21 mutation negative
non-small cell lung cancer/Phase 3
EGFR exon 21 mutation positive
KRAS mutation negative non-small
cell lung cancer/Phase 2
EGFR exon 21 mutation positive
non-small cell lung cancer/Phase 2
EGFR exon 21 mutation positive
non-small cell lung cancer/Phase
2/Phase 3
EGFR exon 21 mutation positive
non-small cell lung cancer/Phase 3
EGFR exon 21 mutation positive
pancreatic cancer/Phase 2
EGFR G719A mutation positive
non-small cell lung cancer/Phase 2
EGFR G719C mutation positive
cancer/Phase 1
EGFR G719C mutation positive
non-small cell lung cancer/Phase 2
EGFR G719C mutation positive
solid tumor/Phase 1
EGFR G719S mutation positive
non-small cell lung cancer/Phase 2
EGFR G719X mutation positive
lung adenocarcinoma/Phase 2
EGFR G719X mutation positive
non-small cell lung cancer/Phase 2
EGFR L858 mutation positive
EGFR T790M mutation negative
EML4-ALK fusion positive non-
small cell lung cancer/Phase 2
EGFR L858 mutation positive
EGFR T790M mutation negative
non-small cell lung cancer/Phase 2
EGFR L858R mutation positive
cancer/Phase 1
EGFR L858R mutation positive
EGFR T790M mutation negative
non-small cell lung
carcinoma/Phase 3
EGFR L858R mutation positive
non-small cell lung cancer/Phase 1
EGFR L858R mutation positive
non-small cell lung cancer/Phase 2
EGFR L858R mutation positive
non-small cell lung cancer/Phase 3
EGFR L858R mutation positive
nonsquamous non small cell lung
carcinoma/Phase 2
EGFR L858R mutation positive
solid tumor/Phase 1
EGFR L861Q mutation positive
lung adenocarcinoma/Phase 2
EGFR L861Q mutation positive
non-small cell lung cancer/Phase 2
EGFR mutated c-MET-amplified
non-small cell lung cancer/Phase 1
EGFR mutation negative non-small
cell lung cancer/Phase 1/Phase 2
EGFR mutation negative non-small
cell lung cancer/Phase 2
EGFR mutation positive
adenocarcinoma of the lung/Phase
2
EGFR mutation positive EGFR
exon 20 insertion activating
mutation negative non-small cell
lung cancer/Phase 2
EGFR mutation positive non small
cell lung carcinoma/Phase 1
EGFR mutation positive non small
cell lung carcinoma/Phase 2
EGFR mutation positive non-small

© 2000-2014 QIAGEN. All rights reserved.

cell lung cancer/Phase 1
EGFR mutation positive non-small cell lung cancer/Phase 1/Phase 2
EGFR mutation positive non-small cell lung cancer/Phase 2
EGFR mutation positive non-small cell lung cancer/Phase 3
EGFR resistant mutation positive cancer/Phase 1
EGFR sensitizing mutation positive cancer/Phase 1
EGFR sensitizing mutation positive non-small cell lung cancer/Phase 1
EGFR sensitizing mutation positive non-small cell lung cancer/Phase 1/Phase 2
EGFR sensitizing mutation positive non-small cell lung cancer/Phase 2
EGFR sensitizing mutation positive non-small cell lung cancer/Phase 3
EGFR sensitizing mutation positive non-small cell lung carcinoma/Phase 1/Phase 2
EGFR sensitizing mutation positive non-squamous non-small cell lung cancer/Phase 2
EGFR sensitizing mutation positive solid tumor/Phase 1
EGFR T790M mutation positive cancer/Phase 1
EGFR T790M mutation positive solid tumor/Phase 1
EGFR TK domain mutation positive malignant peritoneal mesothelioma/Phase 2
EGFR(L858R) positive metastatic non-small cell lung cancer/Approved
endometrial cancer/Phase 2
ependymoma/Phase 2
epithelial ovarian cancer/Phase 1
epithelial ovarian cancer/Phase 1/Phase 2
epithelial ovarian cancer/Phase 2
epithelioid mesothelioma/Phase 2
esophageal adenocarcinoma/Phase 1
esophageal adenocarcinoma/Phase 2
esophageal cancer/Phase 1
esophageal cancer/Phase 2
esophageal cancer/Phase 3
esophageal squamous cell cancer/Phase 3
esophageal squamous cell carcinoma/Phase 1
esophageal squamous cell carcinoma/Phase 2
estrogen receptor positive progesterone receptor positive cancer/Phase 1
estrogen receptor positive progesterone receptor positive solid malignant tumor/Phase 1
estrogen receptor-negative breast cancer/Phase 2
extrahepatic bile duct cancer/Phase 1
extrahepatic bile duct cancer/Phase 2
extrahepatic cholangiocarcinoma/Phase 2
fallopian tube cancer/Phase 1/Phase 2
fallopian tube cancer/Phase 2
fallopian tube cancer/Phase 3
gall bladder adenocarcinoma/Phase 2
gall bladder carcinoma/Phase 2
gallbladder cancer/Phase 1
gallbladder cancer/Phase 2
gastric adenocarcinoma/Phase 1
gastric cancer/Phase 1
gastric cancer/Phase 2
gastrointestinal stromal tumor/Phase 1
gastrointestinal tract cancer/Phase 2
gastrointestinal tract cancer/Unspecified phase
glioblastoma cancer/Phase 1
glioblastoma cancer/Phase 2
glioblastoma/Phase 1/Phase 2
glioblastoma/Phase 2
glioma cancer/Unspecified phase
gliosarcoma cancer/Phase 1
gliosarcoma cancer/Phase 1/Phase 2
gliosarcoma cancer/Phase 2
head and neck cancer/Phase 0
head and neck cancer/Phase 1
head and neck cancer/Phase 1/Phase 2
head and neck cancer/Phase 2
head and neck cancer/Phase 2/Phase 3
head and neck cancer/Phase 3

© 2000-2014 QIAGEN. All rights reserved.

head and neck cancer/Unspecified phase
head and neck squamous cell carcinoma/Phase 1/Phase 2
head and neck squamous cell carcinoma/Phase 2
head and neck tumor/Unspecified phase
hematologic cancer/Phase 2
hepatocellular carcinoma/Phase 1
hepatocellular carcinoma/Phase 1/Phase 2
hepatocellular carcinoma/Phase 2
HER2 activating mutation positive breast cancer/Phase 2
HER2 activating mutation positive gastric cancer/Phase 2
HER2 activating mutation positive gastroesophageal junction cancer/Phase 2
HER2 negative hormone receptor negative breast cancer/Phase 1
HER2 positive solid malignant tumor/Phase 2
HER2-negative breast cancer/Phase 2
HER3 positive carcinoma/Phase 1
hereditary leiomyomatosis and renal cell cancer/Phase 2
hormone receptor positive solid tumor/Phase 1
hypopharyngeal squamous cell carcinoma/Phase 1
hypopharyngeal squamous cell carcinoma/Phase 1/Phase 2
hypopharyngeal squamous cell carcinoma/Phase 2
islet cell tumor/Phase 2
Klatskin's tumor/Phase 2
KRAS codon 12 mutation positive non-small cell lung cancer/Phase 3
KRAS codon 13 mutation positive non-small cell lung cancer/Phase 3
KRAS mutation negative colorectal carcinoma/Phase 1
KRAS mutation negative non-small cell lung cancer/Phase 2
KRAS mutation positive non small cell lung cancer/Phase 1
KRAS mutation positive non small cell lung cancer/Phase 2
KRAS wild-type positive colorectal cancer/Phase 2
large cell lung cancer/Phase 1
large cell lung cancer/Phase 2
large cell lung carcinoma/Phase 2
large-cell carcinoma/Phase 3
laryngeal squamous cell carcinoma/Phase 1
laryngeal squamous cell carcinoma/Phase 1/Phase 2
laryngeal squamous cell carcinoma/Phase 2
laryngeal verrucous carcinoma/Phase 1
laryngeal verrucous carcinoma/Phase 1/Phase 2
laryngeal verrucous carcinoma/Phase 2
leukemia/Phase 2
liver cancer/Phase 1
liver cancer/Phase 1/Phase 2
liver cancer/Phase 2
local neoplasm recurrence/Phase 2
locally advanced EGFR mutation positive lung adenocarcinoma/Phase 2
locally advanced KRAS mutation positive non small cell lung cancer/Phase 2
locally advanced non small cell lung cancer/Approved
locally advanced non small cell lung cancer/Phase 2
locally advanced pancreatic cancer/Phase 2
low-grade glioma/Phase 1
lung adenocarcinoma/Phase 1/Phase 2
lung adenocarcinoma/Phase 2
lung adenocarcinoma/Phase 4
lung adenosquamous cancer/Phase 2
lung cancer/Phase 1
lung cancer/Phase 1/Phase 2
lung cancer/Phase 2
lung cancer/Phase 3
lung cancer/Unspecified phase
lung squamous cell carcinoma/Phase 2
lung tumor/Phase 2
lymphocytic cancer/Phase 1
lymphoepithelial carcinoma/Phase 1/Phase 2
lymphoma/Phase 1
male breast cancer/Phase 1
malignant brain neoplasm/Phase 1

© 2000-2014 QIAGEN. All rights reserved.

malignant glioma/Phase 1/Phase 2
malignant mesothelioma/Phase 1
malignant mesothelioma/Phase 2
malignant solid tumor/Phase 1
malignant thyroid gland neoplasm/Phase 1
mammary tumor/Phase 1
mammary tumor/Phase 2
medulloblastoma/Phase 2
melanoma/Phase 1
melanoma/Phase 2
mesothelioma/Phase 2
metaplasia/Phase 1
metastasis/Phase 1
metastasis/Phase 2
metastasis/Phase 3
metastatic breast cancer/Phase 1
metastatic breast cancer/Phase 2
metastatic colorectal cancer/Phase 2
metastatic colorectal cancer/Phase 3
metastatic KRAS mutation positive non-small-cell lung cancer/Phase 2
metastatic non-small-cell lung cancer/Approved
metastatic non-small-cell lung cancer/Phase 1
metastatic non-small-cell lung cancer/Phase 1/Phase 2
metastatic non-small-cell lung cancer/Phase 2
metastatic non-small-cell lung cancer/Phase 3
metastatic non-small-cell lung cancer/Phase 4
metastatic non-small-cell lung cancer/Unspecified phase
metastatic pancreatic adenocarcinoma/Phase 1
metastatic pancreatic adenocarcinoma/Phase 2
metastatic pancreatic cancer/Phase 1
metastatic pancreatic cancer/Phase 1/Phase 2
metastatic pancreatic cancer/Phase 2
metastatic papillary renal cancer/Phase 2
metastatic solid tumor/Phase 1
metastatic uveal melanoma/Phase 1
mucinous adenocarcinoma of colon/Phase 1
mucinous adenocarcinoma of rectum/Phase 1
multiple endocrine neoplasia/Phase 2
multiple myeloma/Phase 1
muscle invasive bladder cancer/Phase 2
myelodysplastic syndrome/Phase 1/Phase 2
myelodysplastic syndrome/Phase 2
nasopharyngeal cancer/Phase 2
neoplasia/Phase 1
neuroblastoma/Phase 1
neuroblastoma/Phase 2
neuroendocrine tumor/Phase 1
neuroendocrine tumor/Phase 2
non small cell lung adenocarcinoma/Phase 1
non small cell lung adenocarcinoma/Phase 1/Phase 2
non small cell lung adenocarcinoma/Phase 2
non-small cell lung cancer with activating EGFR mutations/Phase 2
non-small cell lung cancer with activating EGFR mutations/Phase 3
non-small cell lung cancer/Phase 0
non-small cell lung cancer/Phase 1
non-small cell lung cancer/Phase 1/Phase 2
non-small cell lung cancer/Phase 2
non-small cell lung cancer/Phase 3
non-small cell lung cancer/Phase 4
non-small cell lung cancer/Unspecified phase
non-small-cell lung carcinoma/Approved
non-squamous non-small cell lung cancer/Phase 2
non-squamous non-small cell lung cancer/Phase 3
occult primary tumor/Phase 2
oral cancer/Phase 3
oropharyngeal squamous-cell carcinoma/Phase 1
oropharyngeal squamous-cell carcinoma/Phase 1/Phase 2
oropharyngeal squamous-cell carcinoma/Phase 2
osteosarcoma/Phase 1
ovarian cancer/Phase 1/Phase 2
ovarian cancer/Phase 2

© 2000-2014 QIAGEN. All rights reserved.

ovarian cancer/Phase 3
ovarian endometrioid carcinoma/Phase 2
ovarian mucinous cystadenocarcinoma/Phase 2
ovarian serous cystadenocarcinoma/Phase 2
ovarian tumor/Phase 2
pancreatic acinar cell carcinoma/Phase 1
pancreatic acinar cell carcinoma/Phase 2/Phase 3
pancreatic adenocarcinoma/Phase 1
pancreatic adenocarcinoma/Phase 1/Phase 2
pancreatic adenocarcinoma/Phase 2
pancreatic cancer/Phase 1
pancreatic cancer/Phase 1/Phase 2
pancreatic cancer/Phase 2
pancreatic cancer/Phase 2/Phase 3
pancreatic cancer/Phase 3
pancreatic carcinoma/Phase 1
pancreatic carcinoma/Phase 2
pancreatic ductal adenocarcinoma/Phase 1
pancreatic ductal adenocarcinoma/Phase 2
pancreatic ductal adenocarcinoma/Phase 2/Phase 3
pancreatic intraductal papillary mucinous tumor/Phase 2
papillary renal cancer/Phase 2
papillary renal cell carcinoma/Phase 1
papillary thyroid cancer/Phase 2
papillary type II renal carcinoma/Phase 2
parotid gland cancer/Phase 1
peritoneal cavity cancer/Phase 1/Phase 2
peritoneal cavity cancer/Phase 2
pharyngeal cancer/Phase 0
polycythemia vera/Phase 2
precancerous condition/Phase 1/Phase 2
primary head and neck squamous cell carcinoma/Phase 1
primary head and neck squamous cell carcinoma/Phase 1/Phase 2
primary laryngeal squamous cell carcinoma/Phase 1/Phase 2
primary oral squamous cell carcinoma/Phase 1/Phase 2
primary peritoneal cancer/Phase 2
primary peritoneal cancer/Phase 3
primary sclerosing cholangitis/Phase 1
progesterone receptor-negative breast cancer/Phase 2
progressive ALK fusion negative EGFR mutation negative squamous cell non-small cell lung cancer/Phase 2/Phase 3
progressive recurrent EGFR activating mutation positive lung cancer/Phase 1/Phase 2
progressive unresectable EGFR activating mutation positive lung cancer/Phase 1/Phase 2
prostate cancer/Phase 1
prostate cancer/Phase 1/Phase 2
prostate cancer/Phase 2
psoriasis/Phase 2
PTCH1 loss-of-function mutation positive malignant solid tumor/Phase 2
rectal adenocarcinoma/Phase 1
rectal adenocarcinoma/Phase 1/Phase 2
rectal adenocarcinoma/Phase 2
rectum cancer/Phase 1
rectum cancer/Phase 1/Phase 2
rectum cancer/Phase 2
recurrent breast cancer/Phase 1
recurrent breast cancer/Phase 2
recurrent EGFR activating mutation positive non-squamous non-small cell lung cancer/Phase 2
recurrent EGFR exon 19 deletion positive non-small cell lung cancer/Phase 2
recurrent EGFR L858R mutation positive non-small cell lung cancer/Phase 2
recurrent laryngeal squamous cell carcinoma/Phase 1
recurrent laryngeal squamous cell carcinoma/Phase 1/Phase 2
recurrent laryngeal squamous cell carcinoma/Phase 2
recurrent prostate cancer/Phase 1
recurrent rectal cancer/Phase 1
recurrent rectal cancer/Phase 1/Phase 2

© 2000-2014 QIAGEN. All rights reserved.

recurrent rectal cancer/Phase 2
renal cancer/Phase 1
renal cancer/Phase 2
renal clear cell adenocarcinoma/Phase 1
renal clear cell adenocarcinoma/Phase 1/Phase 2
renal parenchyma cancer/Phase 1/Phase 2
renal parenchyma cancer/Phase 2
renal parenchyma cancer/Phase 4
renal-cell carcinoma/Phase 1
renal-cell carcinoma/Phase 1/Phase 2
renal-cell carcinoma/Phase 2
ROS1 fusion positive non-small cell lung cancer/Phase 2
salivary gland cancer/Phase 1
salivary gland cancer/Phase 1/Phase 2
salivary gland cancer/Phase 2
sarcoma/Phase 2
signet ring adenocarcinoma of the colon/Phase 1
signet ring adenocarcinoma of the rectum/Phase 1
skin cancer/Phase 1/Phase 2
skin cancer/Phase 2
skin squamous cell carcinoma/Phase 1/Phase 2
skin squamous cell carcinoma/Phase 2
small cell lung cancer/Phase 2
small intestine cancer/Phase 1
SMO activating mutation positive malignant solid tumor/Phase 2
solid tumor/Phase 1
sporadic renal papillary cancer/Phase 2
squamous cell carcinoma of nasopharynx/Phase 1
squamous cell carcinoma of nasopharynx/Phase 1/Phase 2
squamous cell carcinoma of nasopharynx/Phase 2
squamous cell carcinoma of the esophagogastric junction/Phase 3
squamous cell carcinoma of the oral cavity/Phase 1/Phase 2
squamous cell carcinoma/Phase 1
squamous cell carcinoma/Phase 2
squamous cell carcinoma/Phase 2/Phase 3
squamous cell carcinoma/Phase 3
squamous cell lung cancer/Phase 1
squamous cell lung cancer/Phase 2
squamous cell lung cancer/Phase 2/Phase 3
stage 1 rectal cancer/Phase 2
stage 3a non small cell lung carcinoma/Phase 2
stage II gastric cancer/Phase 1
stage II oropharyngeal squamous cell carcinoma/Phase 1
stage III gastric cancer/Phase 1
stage III oropharyngeal squamous cell carcinoma/Phase 1
stage III oropharyngeal squamous cell carcinoma/Phase 1/Phase 2
stage III oropharyngeal squamous cell carcinoma/Phase 2
stage IV gastric cancer/Phase 1
stage IV oropharyngeal squamous cell carcinoma/Phase 1
stage IV oropharyngeal squamous cell carcinoma/Phase 1/Phase 2
stage IV oropharyngeal squamous cell carcinoma/Phase 2
stage IVA oropharyngeal squamous cell carcinoma/Phase 1
stage IVA oropharyngeal squamous cell carcinoma/Phase 1/Phase 2
stage IVB oropharyngeal squamous cell carcinoma/Phase 1
stage IVB oropharyngeal squamous cell carcinoma/Phase 1/Phase 2
stage IVC oropharyngeal squamous cell carcinoma/Phase 1
stage IVC oropharyngeal squamous cell carcinoma/Phase 1/Phase 2
thymoma/Phase 2
thymus cancer/Phase 2
tongue cancer/Phase 1
tongue cancer/Phase 2
transitional cell epithelial cancer/Phase 2
tumor/Phase 1
type M4 acute myeloid leukemia/Phase 2
undifferentiated nasopharyngeal carcinoma/Phase 1
undifferentiated nasopharyngeal carcinoma/Phase 1/Phase 2
unresectable EGFR activating

© 2000-2014 QIAGEN. All rights reserved.
INGENUITY® SYSTEMS

				mutation positive MET-positive non small cell lung cancer/Phase 3
erlotinib/gemcitabine [erlotinib]	EGFR	antagonist	Tarceva/gemcitabine	locally advanced pancreatic cancer/Approved<br>metastatic pancreatic cancer/Approved
erlotinib/vismodegib [erlotinib]	EGFR	inhibitor		
erlotinib/vismodegib [erlotinib]	EGFR	antagonist		
fluconazole	CYP51A1	inhibitor	Biocanol, Biozolene, Diflucan, Elazor, Flucazol, Flucostat, Flukezol, Flunizol, Flusol, Pritenzol, Triflucan	acquired immunodeficiency syndrome/Phase 4<br>advanced solid tumor/Phase 1<br>Alzheimer's disease/Phase 1<br>aspergillosis/Phase 3<br>bacterial disease/Phase 3<br>blastomycosis/Phase 2<br>cancer/Phase 3<br>candidemia/Phase 2<br>candidemia/Phase 3<br>candidiasis of lung/Phase 4<br>candidiasis/Approved<br>candidiasis/Phase 1<br>candidiasis/Phase 2/Phase 3<br>candidiasis/Phase 3<br>candidiasis/Phase 4<br>candidiasis/Unspecified phase<br>cardiorespiratory arrest/Phase 1<br>chronic obstructive pulmonary disease/Phase 1<br>coccidioidomycosis/Phase 3<br>coccidioidomycosis/Unspecified phase<br>cryptococcal meningitis/Approved<br>cryptococcal meningitis/Phase 1/Phase 2<br>cryptococcal meningitis/Phase 2<br>cryptococcal meningitis/Phase 3<br>cryptococcal meningitis/Phase 4<br>cryptococcal meningitis/Unspecified phase<br>cryptococcosis/Phase 4<br>endometriosis/Phase 1<br>esophageal candidiasis/Approved<br>esophageal candidiasis/Phase 3<br>esophageal candidiasis/Unspecified phase<br>fungemia/Phase 3<br>fungemia/Phase 4<br>graft-vs-host disease/Phase 1<br>graft-vs-host disease/Unspecified phase<br>head and neck cancer/Phase 2/Phase 3<br>heart disease/Unspecified phase<br>histoplasmosis/Phase 2<br>histoplasmosis/Unspecified phase<br>hyperlipidemia/Phase 2<br>infection/Phase 1<br>infection/Phase 1/Phase 2<br>infection/Phase 2<br>infection/Phase 2/Phase 3<br>infection/Phase 3<br>infection/Phase 4<br>infection/Unspecified phase<br>leukemia/Phase 3<br>leukemia/Unspecified phase<br>leukopenia/Phase 3<br>localized cutaneous leishmaniasis/Phase 3<br>lymphocytic cancer/Phase 3<br>meningitis/Approved<br>meningitis/Unspecified phase<br>multiple myeloma/Unspecified phase<br>mycosis/Phase 1<br>mycosis/Phase 2<br>mycosis/Phase 3<br>mycosis/Phase 4<br>mycosis/Unspecified phase<br>myelodysplastic syndrome/Unspecified phase<br>oral candidiasis/Phase 2<br>oral candidiasis/Phase 3<br>oral candidiasis/Phase 4<br>oral candidiasis/Unspecified phase<br>oropharyngeal candidiasis/Approved<br>oropharyngeal candidiasis/Phase 4<br>pervasive developmental disorder/Unspecified phase<br>prostate cancer/Phase 1<br>sepsis/Phase 4<br>solid tumor/Phase 1<br>tinea capitis/Phase 3<br>vaginal candidiasis/Approved<br>vaginal discharge/Phase 3<br>vaginitis/Phase 3<br>vascular disease/Unspecified phase<br>very low birth weight/Phase 2
fludarabine phosphate	RRM2	inhibitor	Fludara, Fludura	5q deletion acute myeloid leukemia/Phase 1<br>5q deletion acute myeloid leukemia/Phase 2<br>5q deletion acute myeloid leukemia/Phase 3<br>accelerated phase chronic

© 2000-2014 QIAGEN. All rights reserved.

myelogenous leukemia/Phase 1
accelerated phase chronic myelogenous leukemia/Phase 1/Phase 2
accelerated phase chronic myelogenous leukemia/Phase 2
accelerated phase chronic myelogenous leukemia/Phase 3
accelerated phase chronic myelogenous leukemia/Unspecified phase
acquired aplastic anemia/Phase 2
acute adult T-cell leukemia/Phase 1/Phase 2
acute biphenotypic leukemia/Phase 1
acute biphenotypic leukemia/Phase 2
acute leukemia/Phase 1
acute leukemia/Phase 1/Phase 2
acute leukemia/Phase 2
acute leukemia/Unspecified phase
acute lymphocytic leukemia, type L3/Phase 2
acute lymphocytic leukemia/Phase 1
acute lymphocytic leukemia/Phase 1/Phase 2
acute lymphocytic leukemia/Phase 2
acute lymphocytic leukemia/Phase 2/Phase 3
acute lymphocytic leukemia/Phase 3
acute lymphocytic leukemia/Unspecified phase
acute myeloid leukemia with 11q23 abnormality/Phase 1
acute myeloid leukemia with 11q23 abnormality/Phase 2
acute myeloid leukemia with 11q23 abnormality/Phase 3
acute myeloid leukemia with inv(16)/Phase 1
acute myeloid leukemia with inv(16)/Phase 2
acute myeloid leukemia with multilineage dysplasia following myelodysplastic syndrome/Phase 1
acute myeloid leukemia with multilineage dysplasia following myelodysplastic syndrome/Phase 2
acute myeloid leukemia with multilineage dysplasia following myelodysplastic syndrome/Phase 3
acute myeloid leukemia with multilineage dysplasia following myelodysplastic syndrome/Unspecified phase
acute myeloid leukemia with multilineage dysplasia/Phase 2
acute myeloid leukemia with t(8;21)/Phase 1
acute myeloid leukemia with t(8;21)/Phase 2
acute myeloid leukemia with t(8;21)/Phase 4
acute myeloid leukemia/Phase 1
acute myeloid leukemia/Phase 1/Phase 2
acute myeloid leukemia/Phase 2
acute myeloid leukemia/Phase 3
acute myeloid leukemia/Phase 4
acute myeloid leukemia/Unspecified phase
acute unclassified leukemia/Phase 1/Phase 2
acute unclassified leukemia/Phase 2
acute unclassified leukemia/Unspecified phase
adenocarcinoma/Phase 2
adenosquamous carcinoma/Phase 2
adrenoleukodystrophy/Phase 2
adult acute erythroid leukemia/Phase 2
adult acute lymphocytic leukemia/Phase 1/Phase 2
adult acute lymphocytic leukemia/Phase 2
adult acute lymphocytic leukemia/Phase 4
adult acute lymphocytic leukemia/Unspecified phase
adult acute monocytic leukemia/Phase 2
adult acute monocytic leukemia/Unspecified phase
adult acute myeloid leukemia with 11q23 abnormalities/Phase 1
adult acute myeloid leukemia with 11q23 abnormalities/Phase 1/Phase 2
adult acute myeloid leukemia with 11q23 abnormalities/Phase 2
adult acute myeloid leukemia with

© 2000-2014 QIAGEN. All rights reserved.

11q23 abnormalities/Phase 3
adult acute myeloid leukemia with 11q23 abnormalities/Unspecified phase
adult acute myeloid leukemia with del(5q)/Phase 1
adult acute myeloid leukemia with del(5q)/Phase 1/Phase 2
adult acute myeloid leukemia with del(5q)/Phase 2
adult acute myeloid leukemia with del(5q)/Phase 3
adult acute myeloid leukemia with del(5q)/Unspecified phase
adult acute myeloid leukemia with inv(16)(p13.1q22)/Phase 1
adult acute myeloid leukemia with inv(16)(p13.1q22)/Phase 1/Phase 2
adult acute myeloid leukemia with inv(16)(p13.1q22)/Phase 2
adult acute myeloid leukemia with inv(16)(p13.1q22)/Phase 3
adult acute myeloid leukemia with inv(16)(p13.1q22)/Unspecified phase
adult acute myeloid leukemia with t(16;16)(p13;q22)/Phase 1
adult acute myeloid leukemia with t(16;16)(p13;q22)/Phase 1/Phase 2
adult acute myeloid leukemia with t(16;16)(p13;q22)/Phase 2
adult acute myeloid leukemia with t(16;16)(p13;q22)/Phase 3
adult acute myeloid leukemia with t(16;16)(p13;q22)/Unspecified phase
adult acute myeloid leukemia with t(8;21)(q22;q22)/Phase 1
adult acute myeloid leukemia with t(8;21)(q22;q22)/Phase 1/Phase 2
adult acute myeloid leukemia with t(8;21)(q22;q22)/Phase 2
adult acute myeloid leukemia with t(8;21)(q22;q22)/Phase 3
adult acute myeloid leukemia with t(8;21)(q22;q22)/Unspecified phase
adult acute myeloid leukemia/Phase 2
adult Burkitt lymphoma/Phase 1
adult Burkitt lymphoma/Phase 1/Phase 2
adult Burkitt lymphoma/Phase 2
adult Burkitt lymphoma/Unspecified phase
adult diffuse large-cell lymphoma/Phase 1
adult diffuse large-cell lymphoma/Phase 1/Phase 2
adult diffuse large-cell lymphoma/Phase 2
adult diffuse large-cell lymphoma/Unspecified phase
adult diffuse mixed-cell lymphoma/Phase 1
adult diffuse mixed-cell lymphoma/Phase 1/Phase 2
adult diffuse mixed-cell lymphoma/Phase 2
adult diffuse mixed-cell lymphoma/Unspecified phase
adult Hodgkin lymphoma/Phase 1
adult Hodgkin lymphoma/Phase 1/Phase 2
adult Hodgkin lymphoma/Phase 2
adult Hodgkin lymphoma/Unspecified phase
adult lymphoblastic lymphoma/Phase 1
adult lymphoblastic lymphoma/Phase 1/Phase 2
adult lymphoblastic lymphoma/Phase 2
adult lymphoblastic lymphoma/Unspecified phase
adult Philadelphia chromosome negative precursor B cell acute lymphoblastic leukemia/Phase 3
adult solid tumor/Phase 1
adult solid tumor/Phase 2
adult solid tumor/Phase 2/Phase 3
adult solid tumor/Unspecified phase
adult T cell leukemia/Phase 1
adult T cell leukemia/Phase 1/Phase 2
adult T cell leukemia/Phase 2
adult T cell leukemia/Unspecified phase
advanced cancer/Phase 1
advanced non-small-cell lung cancer/Phase 2
aggressive non-Hodgkin lymphoma/Phase 1/Phase 2
aggressive non-Hodgkin lymphoma/Phase 3
AIDS-related primary CNS lymphoma/Unspecified phase
AIDS-related small noncleaved cell

© 2000-2014 QIAGEN. All rights reserved.

lymphoma/Unspecified phase
ALK fusion negative non-cutaneous anaplastic large cell lymphoma/Phase 2
alpha-mannosidosis/Phase 2
anal cancer/Phase 1/Phase 2
anal cancer/Phase 2
anemia/Phase 1/Phase 2
anemia/Phase 2
angiocentric T-cell lymphoma/Phase 1/Phase 2
angiocentric T-cell lymphoma/Phase 2
angiocentric T-cell lymphoma/Phase 3
angioimmunoblastic T-cell lymphoma/Phase 1
angioimmunoblastic T-cell lymphoma/Phase 1/Phase 2
angioimmunoblastic T-cell lymphoma/Phase 2
angioimmunoblastic T-cell lymphoma/Phase 3
angioimmunoblastic T-cell lymphoma/Unspecified phase
aplastic anemia/Phase 1/Phase 2
aplastic anemia/Phase 2
aplastic anemia/Phase 2/Phase 3
aplastic anemia/Unspecified phase
aspartylglucosaminuria/Phase 2
atypical chronic myeloid leukemia/Phase 1/Phase 2
atypical chronic myeloid leukemia/Phase 2
atypical chronic myeloid leukemia/Unspecified phase
autoimmune lymphoproliferative syndrome/Phase 2
B-cell leukemia/Phase 2
B-cell non-Hodgkin's disease/Phase 2
B-cell prolymphocytic leukemia/Phase 2
benign tumor/Phase 2/Phase 3
benign tumor/Unspecified phase
beta-thalassemia major/Phase 1/Phase 2
beta-thalassemia major/Phase 2
Binet stage C chronic lymphocytic leukemia/Phase 2
bladder carcinoma/Phase 1
blast crisis phase chronic myeloid leukemia/Phase 1/Phase 2
blastic plasmacytoid dendritic cell neoplasm/Unspecified phase
bone marrow failure/Phase 2
bone marrow failure/Phase 2/Phase 3
bone marrow failure/Unspecified phase
BRAF mutation positive melanoma/Phase 1/Phase 2
BRAF V600D positive melanoma/Phase 2
BRAF V600K positive melanoma/Phase 1
BRAF V600K positive melanoma/Phase 2
BRAFV600E positive melanoma/Phase 1
BRAFV600E positive melanoma/Phase 2
BRCA1 mutation positive Fanconi anemia/Phase 2/Phase 3
BRCA2 mutation positive Fanconi anemia/Phase 2
BRCA2 mutation positive Fanconi anemia/Phase 2/Phase 3
breast cancer/Phase 1
breast cancer/Phase 1/Phase 2
breast cancer/Phase 2
Burkitt's lymphoma/Phase 1
Burkitt's lymphoma/Phase 1/Phase 2
Burkitt's lymphoma/Phase 2
Burkitt's lymphoma/Phase 3
Burkitt's lymphoma/Unspecified phase
C-KIT mutation negative inv(16) positive t(16;16) negative acute myeloid leukemia/Phase 2
C-KIT mutation negative t(8;21) positive acute myeloid leukemia/Phase 2
c-KIT mutation positive acute myeloid leukemia/Phase 1/Phase 2
c-MYC positive diffuse large B-cell lymphoma/Phase 2
cancer/Phase 1/Phase 2
cancer/Phase 2
CCND1 negative CD5 positive CD19 positive chronic lymphocytic leukemia/Phase 3
CCND1 negative CD5 positive CD20 positive chronic lymphocytic leukemia/Phase 3
CCND1 negative CD5 positive

© 2000-2014 QIAGEN. All rights reserved.

CD23 positive chronic lymphocytic leukemia/Phase 3
CD20 positive c-MYC positive lymphoma/Phase 2
cervical cancer/Phase 1/Phase 2
cervical cancer/Phase 2
Chediak-Steinbrinck-Higashi syndrome/Phase 2
childhood acute lymphoblastic leukemia/Phase 1
childhood acute lymphoblastic leukemia/Phase 1/Phase 2
childhood acute lymphoblastic leukemia/Phase 2
childhood acute lymphoblastic leukemia/Unspecified phase
childhood acute myeloid leukemia/Phase 1
childhood acute myeloid leukemia/Phase 1/Phase 2
childhood acute myeloid leukemia/Phase 2
childhood acute myeloid leukemia/Phase 3
childhood acute myeloid leukemia/Unspecified phase
childhood anaplastic large cell lymphoma/Phase 1
childhood anaplastic large cell lymphoma/Phase 1/Phase 2
childhood anaplastic large cell lymphoma/Phase 2
childhood anaplastic large cell lymphoma/Unspecified phase
childhood chronic myelogenous leukemia/Phase 1
childhood chronic myelogenous leukemia/Phase 1/Phase 2
childhood chronic myelogenous leukemia/Phase 2
childhood chronic myelogenous leukemia/Unspecified phase
childhood solid tumor/Phase 2
childhood solid tumor/Phase 2/Phase 3
childhood solid tumor/Unspecified phase
cholangiocarcinoma/Phase 2
chronic B-cell leukemia/Discontinued
chronic B-cell leukemia/Phase 1
chronic B-cell leukemia/Phase 1/Phase 2
chronic B-cell leukemia/Phase 2
chronic B-cell leukemia/Phase 3
chronic B-cell leukemia/Phase 4
chronic B-cell leukemia/Unspecified phase
chronic eosinophilic leukemia/Phase 2
chronic eosinophilic leukemia/Unspecified phase
chronic granulomatous disease/Phase 2
chronic granulomatous disease/Unspecified phase
chronic leukemia/Phase 2
chronic lymphocytic leukemia with del(17p)/Phase 1
chronic lymphocytic leukemia/Phase 1
chronic lymphocytic leukemia/Phase 1/Phase 2
chronic lymphocytic leukemia/Phase 2
chronic lymphocytic leukemia/Phase 2/Phase 3
chronic lymphocytic leukemia/Phase 3
chronic lymphocytic leukemia/Phase 4
chronic lymphocytic leukemia/Unspecified phase
chronic myeloid leukemia/Phase 1
chronic myeloid leukemia/Phase 1/Phase 2
chronic myeloid leukemia/Phase 2
chronic myeloid leukemia/Phase 3
chronic myeloid leukemia/Unspecified phase
chronic myelomonocytic leukemia/Phase 1
chronic myelomonocytic leukemia/Phase 1/Phase 2
chronic myelomonocytic leukemia/Phase 2
chronic myelomonocytic leukemia/Phase 3
chronic myelomonocytic leukemia/Unspecified phase
chronic myeloproliferative disorder/Phase 1
chronic myeloproliferative disorder/Phase 1/Phase 2
chronic myeloproliferative disorder/Phase 2
chronic myeloproliferative

© 2000-2014 QIAGEN. All rights reserved.

disorder/Phase 2/Phase 3
chronic myeloproliferative disorder/Unspecified phase
chronic neutrophilic leukemia/Phase 2
chronic neutrophilic leukemia/Unspecified phase
chronic phase chronic myeloid leukemia/Phase 1
chronic phase chronic myeloid leukemia/Phase 1/Phase 2
chronic phase chronic myeloid leukemia/Phase 2
chronic phase chronic myeloid leukemia/Phase 3
chronic phase chronic myeloid leukemia/Unspecified phase
clear-cell adenocarcinoma/Phase 1
cold agglutinin disease/Phase 2
colorectal cancer/Phase 1
common variable immunodeficiency/Phase 2
congenital amegakaryocytic thrombocytopenia/Phase 1
congenital amegakaryocytic thrombocytopenia/Phase 1/Phase 2
congenital amegakaryocytic thrombocytopenia/Phase 2
congenital dyserythropoietic anemia/Phase 1/Phase 2
Crohn's disease/Phase 2
cutaneous B-cell lymphoma/Phase 1
cutaneous B-cell lymphoma/Phase 1/Phase 2
cutaneous B-cell lymphoma/Phase 2
cutaneous B-cell lymphoma/Phase 3
cutaneous B-cell lymphoma/Unspecified phase
cutaneous T-cell lymphoma/Phase 1
cutaneous T-cell lymphoma/Phase 1/Phase 2
cutaneous T-cell lymphoma/Phase 2
cutaneous T-cell lymphoma/Phase 3
cutaneous T-cell lymphoma/Unspecified phase
cyclin D1 negative chronic lymphocytic leukemia/Phase 2
cyclin D1 negative small lymphocytic lymphoma/Phase 2
cyclin D1 negative small lymphocytic lymphoma/Phase 3
cyclin D1 positive mantle cell lymphoma/Phase 1/Phase 2
cyclin D1 positive mantle cell lymphoma/Phase 3
DEK-NUP214 mutation positive acute myeloid leukemia/Phase 2
del 17p mutation multiple myeloma/Phase 2
del13 mutation multiple myeloma/Phase 2
Diamond-Blackfan anemia/Phase 1/Phase 2
Diamond-Blackfan anemia/Phase 2
Diamond-Blackfan anemia/Phase 2/Phase 3
diffuse large B-cell lymphoma/Phase 1
diffuse large B-cell lymphoma/Phase 1/Phase 2
diffuse large B-cell lymphoma/Phase 2
diffuse large B-cell lymphoma/Phase 3
diffuse large B-cell lymphoma/Unspecified phase
diffuse mixed-cell lymphoma/Phase 2
diffuse mixed-cell lymphoma/Phase 3
diffuse small cleaved-cell lymphoma/Phase 2
diffuse small cleaved-cell lymphoma/Phase 3
DiGeorge syndrome/Phase 2
DNMT3A mutation positive acute myeloid leukemia/Phase 1/Phase 2
DOCK8 deficiency/Phase 2
double mutated CEBP-alpha acute myeloid leukemia/Phase 2
dyskeratosis congenita/Phase 2/Phase 3
dyskeratosis congenita/Unspecified phase
EGFRvIII mutation positive glioblastoma/Phase 1/Phase 2
EGFRvIII mutation positive gliosarcoma/Phase 1/Phase 2
epidermolysis bullosa/Phase 2
epidermolysis bullosa/Unspecified

© 2000-2014 QIAGEN. All rights reserved.

phase
esophageal cancer/Phase 2
esophageal carcinoma/Phase 1
essential thrombocythemia/Phase 2
essential thrombocythemia/Unspecified phase
Ewing's sarcoma/Phase 1
extramedullary plasmacytoma/Unspecified phase
fallopian tube cancer/Phase 2
fallopian tube carcinoma/Phase 1
FANCC exon 14 mutation positive Fanconi anemia/Phase 2/Phase 3
Fanconi's anemia/Phase 1
Fanconi's anemia/Phase 1/Phase 2
Fanconi's anemia/Phase 2
Fanconi's anemia/Phase 2/Phase 3
Fanconi's anemia/Unspecified phase
FLT3 activating mutation positive acute myeloid leukemia/Phase 1
FLT3 activating mutation positive acute myeloid leukemia/Phase 1/Phase 2
FLT3 activating mutation positive acute myeloid leukemia/Phase 2
FLT3-ITD mutated acute myeloid leukemia/Phase 1/Phase 2
FLT3-ITD mutated acute myeloid leukemia/Phase 2
FLT3-ITD mutation negative NPM1 mutation positive acute myeloid leukemia/Phase 2
follicular non-Hodgkin's disease/Phase 1
follicular non-Hodgkin's disease/Phase 1/Phase 2
follicular non-Hodgkin's disease/Phase 2
follicular non-Hodgkin's disease/Phase 2/Phase 3
follicular non-Hodgkin's disease/Phase 3
follicular non-Hodgkin's disease/Unspecified phase
fucosidosis/Phase 2
gangliosidosis/Phase 2
gastric cancer/Phase 1
gastrointestinal tumor/Phase 1
GATA2 mutation positive myelodysplastic syndrome/Phase 2
germ cell tumor/Phase 1
glioblastoma/Phase 1/Phase 2
grade 1 follicular lymphoma/Phase 1
grade 1 follicular lymphoma/Phase 2
grade 1 follicular lymphoma/Phase 3
grade 1 follicular lymphoma/Unspecified phase
grade 2 follicular lymphoma/Phase 1
grade 2 follicular lymphoma/Phase 2
grade 2 follicular lymphoma/Phase 3
grade 2 follicular lymphoma/Unspecified phase
grade 3 follicular lymphoma/Phase 1
grade 3 follicular lymphoma/Phase 1/Phase 2
grade 3 follicular lymphoma/Phase 2
grade 3 follicular lymphoma/Phase 3
grade 3 follicular lymphoma/Unspecified phase
graft-vs-host disease/Phase 1
graft-vs-host disease/Phase 1/Phase 2
graft-vs-host disease/Phase 2
graft-vs-host disease/Phase 2/Phase 3
graft-vs-host disease/Unspecified phase
Griscelli syndrome/Phase 2
hairy-cell leukemia/Phase 1
hairy-cell leukemia/Phase 1/Phase 2
hairy-cell leukemia/Phase 2
head and neck cancer/Phase 1
hematologic cancer/Phase 1
hematologic cancer/Phase 1/Phase 2
hematologic cancer/Phase 2
hematological disorder/Phase 2
hematological system tumor/Phase 2
hemoglobin SC disease/Phase 2
hemoglobin SC disease/Unspecified phase
hemoglobinopathy/Phase 2
hemoglobinopathy/Unspecified phase

© 2000-2014 QIAGEN. All rights reserved.

hemophagocytic lymphohistiocytosis/Phase 1/Phase 2
hemophagocytic lymphohistiocytosis/Phase 2
HER2 positive osteosarcoma/Phase 1
HER2 positive sarcoma/Phase 1
high-risk karyotype acute myeloid leukemia/Phase 2
Hodgkin's disease/Phase 1
Hodgkin's disease/Phase 1/Phase 2
Hodgkin's disease/Phase 2
Hodgkin's disease/Phase 2/Phase 3
Hodgkin's disease/Phase 3
Hodgkin's disease/Unspecified phase
Hurler's disease/Phase 2
hypodiploid multiple myeloma/Phase 2
IGH/CCND1 negative small lymphocytic lymphoma/Phase 3
IKZF1 mutation positive acute myeloid leukemia/Phase 2
immunodeficiency/Phase 1
immunodeficiency/Phase 1/Phase 2
immunodeficiency/Phase 2
indolent non-Hodgkin lymphoma/Phase 1/Phase 2
indolent non-Hodgkin lymphoma/Phase 3
infantile malignant osteopetrosis/Phase 1/Phase 2
infection/Phase 2
infection/Unspecified phase
juvenile myelomonocytic leukemia/Phase 1
juvenile myelomonocytic leukemia/Phase 1/Phase 2
juvenile myelomonocytic leukemia/Phase 2
juvenile myelomonocytic leukemia/Unspecified phase
Krabbe's disease/Phase 2
Langerhans cell histiocytosis/Phase 2
large-cell diffuse lymphoma/Phase 2
large-cell diffuse lymphoma/Phase 3
large-cell Ki-1 lymphoma/Phase 1
large-cell Ki-1 lymphoma/Phase 1/Phase 2
large-cell Ki-1 lymphoma/Phase 2
large-cell Ki-1 lymphoma/Phase 3
large-cell Ki-1 lymphoma/Unspecified phase
large-cell lymphoma/Phase 2
large-cell lymphoma/Phase 3
leukemia/Phase 1
leukemia/Phase 1/Phase 2
leukemia/Phase 2
leukemia/Phase 2/Phase 3
leukemia/Phase 3
leukemia/Unspecified phase
low-grade non Hodgkin's lymphoma/Phase 2
lung cancer/Phase 1
lupus erythematosus/Phase 1/Phase 2
lymphoblastic lymphoma/Phase 1/Phase 2
lymphoblastic lymphoma/Phase 2
lymphoblastic lymphoma/Phase 3
lymphoblastic lymphoma/Unspecified phase
lymphocytic cancer/Phase 1
lymphocytic cancer/Phase 1/Phase 2
lymphocytic cancer/Phase 2
lymphocytic cancer/Phase 2/Phase 3
lymphocytic cancer/Phase 3
lymphocytic cancer/Phase 4
lymphocytic cancer/Unspecified phase
lymphocytic leukemia/Phase 2
lymphocytic leukemia/Phase 4
lymphocytic leukemia/Unspecified phase
lymphomatoid granulomatosis/Phase 2
lymphomatoid granulomatosis/Phase 3
lymphoplasmacytoid lymphoma/Phase 1/Phase 2
lymphoplasmacytoid lymphoma/Phase 2
lymphoplasmacytoid lymphoma/Phase 3
lymphoplasmacytoid lymphoma/Unspecified phase
lymphoproliferative disorder/Phase 1/Phase 2

© 2000-2014 QIAGEN. All rights reserved.
INGENUITY® SYSTEMS

lymphoproliferative disorder/Phase 2
M1 adult acute myeloid leukemia/Phase 2
M1 adult acute myeloid leukemia/Unspecified phase
M1 childhood acute myeloid leukemia/Phase 3
M2 adult acute myeloid leukemia/Phase 2
M2 adult acute myeloid leukemia/Unspecified phase
M2 childhood acute myeloid leukemia/Phase 3
M4 adult acute myeloid leukemia/Phase 2
M4 adult acute myeloid leukemia/Unspecified phase
M4 childhood acute myeloid leukemia/Phase 3
M7 childhood acute myeloid leukemia/Phase 2
M7 childhood acute myeloid leukemia/Phase 3
malignant cutaneous melanoma cancer/Phase 1
malignant cutaneous melanoma/Phase 1
malignant cutaneous melanoma/Phase 1/Phase 2
malignant cutaneous melanoma/Phase 2
malignant lymphoid neoplasm/Phase 1/Phase 2
malignant lymphoid neoplasm/Unspecified phase
malignant myeloid neoplasm/Phase 1/Phase 2
malignant myeloid neoplasm/Unspecified phase
malignant tumor/Phase 2
mammary tumor/Phase 1/Phase 2
mantle cell lymphoma/Phase 1
mantle cell lymphoma/Phase 1/Phase 2
mantle cell lymphoma/Phase 2
mantle cell lymphoma/Phase 3
mantle cell lymphoma/Unspecified phase
marginal zone cell lymphoma/Phase 1
marginal zone cell lymphoma/Phase 1/Phase 2
marginal zone cell lymphoma/Phase 2
marginal zone cell lymphoma/Phase 3
marginal zone cell lymphoma/Unspecified phase
mast cell leukemia/Phase 1/Phase 2
mast cell leukemia/Phase 2
mast cell leukemia/Unspecified phase
mastocytosis/Phase 2
mediastinal large B-cell lymphoma/Phase 1
melanoma metastasis/Phase 1
melanoma metastasis/Phase 1/Phase 2
melanoma metastasis/Phase 2
melanoma metastasis/Phase 3
melanoma metastasis/Unspecified phase
melanoma/Phase 1
melanoma/Phase 1/Phase 2
melanoma/Phase 2
mesothelioma/Phase 1/Phase 2
metabolic disorder/Unspecified phase
metachromatic leukodystrophy/Phase 2
metastasis/Phase 1
metastasis/Phase 1/Phase 2
metastasis/Phase 2
metastatic colorectal cancer/Phase 2
metastatic cutaneous melanoma/Phase 2
metastatic gastric cancer/Phase 2
metastatic hepatocellular carcinoma/Phase 2
metastatic non-small-cell lung cancer/Phase 2
metastatic pancreatic cancer/Phase 2
metastatic uveal melanoma/Phase 2
mixed-cell follicular lymphoma/Phase 1
MLL positive infantile acute lymphoblastic leukemia/Phase 1/Phase 2
MLL translocation acute lymphocytic leukemia/Phase 1
MLL translocation acute lymphocytic leukemia/Phase 2

© 2000-2014 QIAGEN. All rights reserved.

monosomy 7 acute myeloid leukemia/Phase 2
mucolipidosis, type II/Phase 2
mucopolysaccharidosis I/Phase 2
mucopolysaccharidosis type VI/Phase 2
mucopolysaccharidosis, type II/Phase 2
mucosa-associated lymphoid tissue lymphoma/Phase 2
multiple myeloma/Phase 0
multiple myeloma/Phase 1
multiple myeloma/Phase 1/Phase 2
multiple myeloma/Phase 2
multiple myeloma/Phase 2/Phase 3
multiple myeloma/Phase 3
multiple myeloma/Phase 4
multiple myeloma/Unspecified phase
multiple sclerosis/Phase 2
myelodysplastic myeloproliferative neoplasm/Phase 1
myelodysplastic myeloproliferative neoplasm/Phase 1/Phase 2
myelodysplastic myeloproliferative neoplasm/Phase 2
myelodysplastic myeloproliferative neoplasm/Phase 3
myelodysplastic myeloproliferative neoplasm/Unspecified phase
myelodysplastic syndrome with 5q-syndrome/Phase 2
myelodysplastic syndrome/Phase 1
myelodysplastic syndrome/Phase 1/Phase 2
myelodysplastic syndrome/Phase 2
myelodysplastic syndrome/Phase 2/Phase 3
myelodysplastic syndrome/Phase 3
myelodysplastic syndrome/Phase 4
myelodysplastic syndrome/Unspecified phase
myelodysplastic-myeloproliferative disease/Phase 1
myelodysplastic-myeloproliferative disease/Phase 2
myelodysplastic-myeloproliferative disease/Phase 2/Phase 3
myelodysplastic-myeloproliferative disease/Unspecified phase
myelofibrosis with myeloid metaplasia/Phase 2
myelofibrosis with myeloid metaplasia/Unspecified phase
myelofibrosis/Phase 1
myelofibrosis/Phase 2
myelofibrosis/Unspecified phase
myeloid leukemia/Phase 2
myeloid sarcoma/Phase 2
myelomonocytic leukemia/Phase 2
myeloproliferative disorder/Phase 1
myeloproliferative disorder/Phase 1/Phase 2
myeloproliferative disorder/Phase 2
myeloproliferative disorder/Phase 4
myeloproliferative disorder/Unspecified phase
natural killer cell leukemia/Phase 2
natural killer cell leukemia/Unspecified phase
neoplasia/Phase 1/Phase 2
neuroblastoma/Phase 0
neuroblastoma/Phase 1
neuroblastoma/Phase 1/Phase 2
neuroblastoma/Phase 2
neutropenia/Phase 1/Phase 2
non-Hodgkin's disease/Phase 1
non-Hodgkin's disease/Phase 1/Phase 2
non-Hodgkin's disease/Phase 2
non-Hodgkin's disease/Phase 2/Phase 3
non-Hodgkin's disease/Phase 3
non-Hodgkin's disease/Unspecified phase
ocular melanoma/Phase 2
oropharyngeal cancer/Phase 1/Phase 2
oropharyngeal cancer/Phase 2
osteopetrosis/Phase 2
osteopetrosis/Phase 2/Phase 3
ovarian cancer/Phase 1
ovarian cancer/Phase 2
ovarian metastasis/Phase 1
pancreatic cancer/Phase 1
pancreatic cancer/Phase 1/Phase 2
pancytopenia/Phase 1/Phase 2
papillary type II renal carcinoma/Phase 1/Phase 2
papillary type II renal carcinoma/Unspecified phase
paroxysmal nocturnal hemoglobinuria/Phase 1/Phase 2
paroxysmal nocturnal hemoglobinuria/Phase 2
paroxysmal nocturnal hemoglobinuria/Unspecified phase

© 2000-2014 QIAGEN. All rights reserved.

pediatric cancer/Phase 2
penis cancer/Phase 1/Phase 2
penis cancer/Phase 2
peripheral T-cell lymphoma/Phase 1
peripheral T-cell lymphoma/Phase 1/Phase 2
peripheral T-cell lymphoma/Phase 2
peripheral T-cell lymphoma/Phase 3
peripheral T-cell lymphoma/Unspecified phase
peritoneal cavity cancer/Phase 2
peroxisomal disorder/Phase 2
Philadelphia chromosome negative chronic myelogenous leukemia/Phase 2
Philadelphia chromosome-negative acute lymphoblastic leukemia/Phase 3
Philadelphia chromosome-negative acute lymphoblastic leukemia/Phase 4
Philadelphia positive adult precursor cell acute lymphoblastic leukemia/Phase 2
Philadelphia positive childhood chronic myelogenous leukemia/Phase 2
Philadelphia positive childhood precursor cell acute lymphoblastic leukemia/Phase 2
Philadelphia-positive acute lymphoblastic leukemia/Phase 1
Philadelphia-positive acute lymphoblastic leukemia/Phase 1/Phase 2
Philadelphia-positive acute lymphoblastic leukemia/Phase 2
Philadelphia-positive acute lymphoblastic leukemia/Phase 3
Philadelphia-positive chronic myeloid leukemia/Phase 1/Phase 2
Philadelphia-positive chronic myeloid leukemia/Phase 2
plasma cell dyscrasia/Phase 1/Phase 2
plasma cell leukemia/Phase 2
plasma cell leukemia/Unspecified phase
plasma cell neoplasm/Phase 1
plasma cell neoplasm/Phase 1/Phase 2
plasma cell neoplasm/Phase 2
plasmablastic lymphoma/Phase 2
pleural mesothelioma/Phase 1/Phase 2
PML-RARA mutation positive acute myeloid leukemia/Phase 1
PML-RARA mutation positive acute myeloid leukemia/Phase 2
PML-RARA mutation positive adult acute myeloid leukemia/Phase 1
PML-RARA mutation positive adult acute myeloid leukemia/Phase 1/Phase 2
PML-RARA mutation positive adult acute myeloid leukemia/Phase 2
PML-RARA mutation positive adult acute myeloid leukemia/Phase 3
PML-RARA mutation positive adult acute myeloid leukemia/Unspecified phase
polycythemia vera/Phase 2
polycythemia vera/Unspecified phase
post-transplant lymphoproliferative disorder/Phase 1/Phase 2
post-transplant lymphoproliferative disorder/Phase 2
post-transplant lymphoproliferative disorder/Phase 4
post-transplant lymphoproliferative disorder/Unspecified phase
precancerous condition/Phase 1/Phase 2
precancerous condition/Phase 2
precancerous condition/Unspecified phase
precursor T-cell lymphoblastic leukemia-lymphoma/Phase 2
primary central nervous system lymphoma/Unspecified phase
primary peritoneal cancer/Phase 1
primary peritoneal cancer/Phase 2
prolymphocytic leukemia/Phase 1
prolymphocytic leukemia/Phase 1/Phase 2
prolymphocytic leukemia/Phase 2
prolymphocytic leukemia/Unspecified phase
prostate cancer/Phase 1
psoriasis/Phase 2
psoriatic arthritis/Phase 2
PTPN11 mutation negative juvenile

© 2000-2014 QIAGEN. All rights reserved.

myelomonocytic leukemia/Phase 2
RAS mutation positive acute myeloid leukemia/Phase 1/Phase 2
recurrent adult acute lymphocytic leukemia/Phase 1
recurrent adult acute lymphocytic leukemia/Phase 1/Phase 2
recurrent adult acute lymphocytic leukemia/Phase 2
recurrent adult acute lymphocytic leukemia/Unspecified phase
recurrent adult acute myeloid leukemia/Phase 1
recurrent adult acute myeloid leukemia/Phase 1/Phase 2
recurrent adult acute myeloid leukemia/Phase 2
recurrent adult acute myeloid leukemia/Phase 3
recurrent adult acute myeloid leukemia/Unspecified phase
recurrent adult diffuse large-cell lymphoma/Phase 1
recurrent adult diffuse large-cell lymphoma/Phase 1/Phase 2
recurrent adult diffuse large-cell lymphoma/Phase 2
recurrent adult diffuse large-cell lymphoma/Unspecified phase
recurrent cancer/Phase 1/Phase 2
recurrent childhood acute lymphoblastic leukemia/Phase 1
recurrent childhood acute lymphoblastic leukemia/Phase 1/Phase 2
recurrent childhood acute lymphoblastic leukemia/Phase 2
recurrent childhood acute lymphoblastic leukemia/Unspecified phase
recurrent grade 1 follicular lymphoma/Phase 1
recurrent grade 1 follicular lymphoma/Phase 1/Phase 2
recurrent grade 1 follicular lymphoma/Phase 2
recurrent grade 1 follicular lymphoma/Unspecified phase
recurrent grade 2 follicular lymphoma/Phase 1
recurrent grade 2 follicular lymphoma/Phase 1/Phase 2
recurrent grade 2 follicular lymphoma/Phase 2
recurrent grade 2 follicular lymphoma/Unspecified phase
recurrent grade 3 follicular lymphoma/Phase 1
recurrent grade 3 follicular lymphoma/Phase 1/Phase 2
recurrent grade 3 follicular lymphoma/Phase 2
recurrent grade 3 follicular lymphoma/Unspecified phase
recurrent melanoma/Phase 1/Phase 2
recurrent melanoma/Phase 2
recurrent non Hodgkin's lymphoma/Unspecified phase
refractory anemia with excess blasts in transformation/Phase 1
refractory anemia with excess blasts in transformation/Phase 2
refractory anemia with excess blasts in transformation/Phase 3
refractory anemia with excess blasts/Phase 1
refractory anemia with excess blasts/Phase 2
refractory anemia with excess blasts/Phase 3
refractory anemia with ringed sideroblasts/Phase 1
refractory anemia with ringed sideroblasts/Phase 1/Phase 2
refractory anemia with ringed sideroblasts/Phase 2
refractory anemia with ringed sideroblasts/Phase 3
refractory anemia/Phase 1
refractory anemia/Phase 1/Phase 2
refractory anemia/Phase 2
refractory anemia/Phase 3
refractory B-cell non-Hodgkin lymphoma/Phase 2
refractory chronic lymphocytic leukemia/Phase 1
refractory chronic lymphocytic leukemia/Phase 1/Phase 2
refractory chronic lymphocytic leukemia/Phase 2
refractory chronic lymphocytic leukemia/Phase 3
refractory chronic lymphocytic leukemia/Unspecified phase
refractory cytopenia with multilineage dysplasia/Phase 1

© 2000-2014 QIAGEN. All rights reserved.

refractory cytopenia with multilineage dysplasia/Phase 2
refractory hairy cell leukemia/Phase 1
refractory hairy cell leukemia/Phase 1/Phase 2
refractory hairy cell leukemia/Phase 2
refractory hairy cell leukemia/Unspecified phase
refractory multiple myeloma/Phase 1
refractory multiple myeloma/Phase 1/Phase 2
refractory multiple myeloma/Phase 2
refractory multiple myeloma/Phase 3
refractory multiple myeloma/Unspecified phase
refractory non Hodgkin's lymphoma/Phase 2
refractory non Hodgkin's lymphoma/Unspecified phase
relapsed acute myeloid leukemia/Phase 1
relapsed acute myeloid leukemia/Phase 2
relapsed acute myeloid leukemia/Phase 3
relapsed Hodgkin disease/Phase 1/Phase 2
relapsed Hodgkin disease/Phase 2
relapsed Hodgkin disease/Phase 3
relapsed Hodgkin disease/Unspecified phase
relapsed mantle cell lymphoma/Phase 1
relapsed mantle cell lymphoma/Phase 1/Phase 2
relapsed mantle cell lymphoma/Phase 2
relapsed mantle cell lymphoma/Phase 3
relapsed mantle cell lymphoma/Unspecified phase
relapsed multiple myeloma/Phase 1
renal cancer/Phase 1
renal cancer/Phase 1/Phase 2
renal cancer/Phase 2
renal cancer/Unspecified phase
renal clear cell adenocarcinoma/Phase 1/Phase 2
renal clear cell adenocarcinoma/Unspecified phase
renal medullary carcinoma/Phase 1/Phase 2
renal parenchyma cancer/Phase 1/Phase 2
renal parenchyma cancer/Phase 2
renal parenchyma cancer/Unspecified phase
renal tumor/Phase 1
renal-cell carcinoma/Phase 1
rhabdoid tumor/Phase 1
rhabdomyosarcoma/Phase 1
rhabdomyosarcoma/Phase 1/Phase 2
rheumatoid arthritis/Phase 2
sarcoma/Phase 2
scleroderma/Phase 1
secondary acute myeloid leukemia/Phase 1
secondary acute myeloid leukemia/Phase 1/Phase 2
secondary acute myeloid leukemia/Phase 2
secondary acute myeloid leukemia/Phase 3
secondary acute myeloid leukemia/Unspecified phase
secondary myelofibrosis/Phase 1
secondary myelofibrosis/Phase 2
secondary myelofibrosis/Unspecified phase
severe aplastic anemia/Phase 1/Phase 2
severe aplastic anemia/Phase 2
severe combined immunodeficiency/Phase 1/Phase 2
severe combined immunodeficiency/Phase 2
severe combined immunodeficiency/Phase 2/Phase 3
severe congenital neutropenia/Phase 1
severe congenital neutropenia/Phase 1/Phase 2
severe congenital neutropenia/Phase 2
severe congenital neutropenia/Phase 2/Phase 3
Shwachman syndrome/Phase 2
Shwachman syndrome/Phase

© 2000-2014 QIAGEN. All rights reserved.

2/Phase 3
sickle cell anemia/Phase 1
sickle cell anemia/Phase 1/Phase 2
sickle cell anemia/Phase 2
sickle cell anemia/Phase 2/Phase 3
sickle cell anemia/Unspecified phase
skin cancer/Phase 1/Phase 2
skin cancer/Phase 2
small cleaved-cell follicular lymphoma/Phase 1
small intestine cancer/Phase 2
small noncleaved-cell lymphoma/Phase 2
small noncleaved-cell lymphoma/Phase 3
solid tumor/Phase 1
solid tumor/Phase 1/Phase 2
solid tumor/Phase 2
sphingolipidosis/Phase 2
splenic marginal zone B-cell lymphoma/Phase 1
splenic marginal zone B-cell lymphoma/Phase 1/Phase 2
splenic marginal zone B-cell lymphoma/Phase 2
splenic marginal zone B-cell lymphoma/Phase 3
splenic marginal zone B-cell lymphoma/Unspecified phase
squamous cell carcinoma/Phase 2
stage I multiple myeloma/Phase 1
stage I multiple myeloma/Phase 2
stage I multiple myeloma/Phase 3
stage I multiple myeloma/Unspecified phase
stage II multiple myeloma/Phase 1
stage II multiple myeloma/Phase 1/Phase 2
stage II multiple myeloma/Phase 2
stage II multiple myeloma/Phase 3
stage II multiple myeloma/Unspecified phase
stage III melanoma/Phase 2
stage III multiple myeloma/Phase 1
stage III multiple myeloma/Phase 1/Phase 2
stage III multiple myeloma/Phase 2
stage III multiple myeloma/Phase 3
stage III multiple myeloma/Unspecified phase
stage IV adult diffuse large cell lymphoma/Phase 1
stage IV adult diffuse large cell lymphoma/Phase 1/Phase 2
stage IV adult diffuse large cell lymphoma/Phase 2
stage IV adult diffuse large cell lymphoma/Unspecified phase
synovial sarcoma/Phase 1
synovitis/Phase 2
systemic amyloidosis/Phase 1/Phase 2
systemic amyloidosis/Unspecified phase
systemic lupus erythematosus/Phase 1
systemic lupus erythematosus/Phase 2
systemic sclerosis/Phase 1
systemic sclerosis/Phase 1/Phase 2
systemic sclerosis/Phase 2
t(14;16) mutation multiple myeloma/Phase 2
t(14;20) mutation positive multiple myeloma/Phase 1
t(16;16)(p13;q22) acute myeloid leukemia/Phase 1
t(16;16)(p13;q22) acute myeloid leukemia/Phase 2
t(1;14) multiple myeloma/Phase 2
t(4;11) acute lymphocytic leukemia/Phase 1
t(4;11) acute lymphocytic leukemia/Phase 2
T-cell large granular lymphocyte leukemia/Phase 1/Phase 2
T-cell large granular lymphocyte leukemia/Phase 2
T-cell large granular lymphocyte leukemia/Unspecified phase
T-cell prolymphocytic leukemia/Phase 2
TERT mutation positive aplastic anemia/Phase 2
testicular germ cell tumor/Phase 1
TET2 mutation positive acute myeloid leukemia/Phase 1/Phase 2
thalassemia/Phase 2
thalassemia/Phase 2/Phase 3
thalassemia/Unspecified phase
thrombocytopenia/Phase 1/Phase 2
thyroid cancer/Phase 1/Phase 2
TP53 mutation positive chronic lymphocytic leukemia/Phase 1
TP53 mutation positive chronic

© 2000-2014 QIAGEN. All rights reserved.

				lymphocytic leukemia/Phase 2<br>transformed non Hodgkin's lymphoma/Phase 2<br>transient myeloproliferative disorder/Phase 3<br>treatment-related acute myeloid leukemia/Phase 1/Phase 2<br>treatment-related acute myeloid leukemia/Phase 2<br>type M6 acute myeloid leukemia/Phase 1/Phase 2<br>type M7 acute myeloid leukemia/Phase 1/Phase 2<br>type M7 acute myeloid leukemia/Phase 2<br>unresectable BRAF mutation positive melanoma/Phase 1/Phase 2<br>untreated adult acute myeloid leukemia/Phase 1<br>untreated adult acute myeloid leukemia/Phase 1/Phase 2<br>untreated adult acute myeloid leukemia/Phase 2<br>untreated adult acute myeloid leukemia/Unspecified phase<br>uveal melanoma/Phase 1<br>vaginal cancer/Phase 1/Phase 2<br>vaginal cancer/Phase 2<br>Waldenstrom's macroglobulinemia/Phase 1<br>Waldenstrom's macroglobulinemia/Phase 1/Phase 2<br>Waldenstrom's macroglobulinemia/Phase 2<br>Waldenstrom's macroglobulinemia/Phase 3<br>Waldenstrom's macroglobulinemia/Unspecified phase<br>Wilm's tumorigenesis/Phase 1<br>Wiskott-Aldrich syndrome/Phase 1/Phase 2<br>Wiskott-Aldrich syndrome/Phase 2<br>X-linked agammaglobulinemia/Phase 2<br>X-linked lymphoproliferative syndrome/Phase 2
fludarabine phosphate/mitoxantrone [fludarabine phosphate]	RRM2	inhibitor		
fludarabine phosphate/mitoxantrone/rituximab [fludarabine phosphate]	RRM2	inhibitor		
fludarabine phosphate/rituximab [fludarabine phosphate]	RRM2	inhibitor		
gallium nitrate	RRM2	inhibitor	Ganite	central nervous system tumor/Phase 1<br>childhood solid tumor/Phase 1<br>cystic fibrosis/Phase 1<br>cystic fibrosis/Phase 2<br>hypercalcemia associated with neoplasia/Approved<br>hypercalcemia/Approved<br>intermediate-grade lymphoma/Phase 2<br>low-grade lymphoma/Phase 2<br>lymphocytic cancer/Phase 1<br>lymphocytic cancer/Phase 2<br>neuroblastoma/Phase 1<br>non-Hodgkin lymphoma/Approved<br>non-Hodgkin's disease/Phase 2<br>sarcoma/Phase 1
gefitinib	EGFR	inhibitor	Iressa, Irressat	activating EGFR mutation positive non small cell lung carcinoma/Phase 2<br>acute myeloid leukemia/Phase 2<br>adult solid tumor/Phase 1<br>advanced non-small-cell lung cancer/Phase 2<br>anaplastic thyroid cancer/Unspecified phase<br>bladder cancer/Phase 2<br>bladder cancer/Phase 3<br>brain metastasis associated with EGFR mutation positive non-small cell lung cancer/Phase 2<br>brain metastasis/Phase 2<br>brain metastasis/Unspecified phase<br>brain tumor/Phase 2<br>breast cancer/Phase 1/Phase 2<br>breast cancer/Phase 2<br>breast cancer/Phase 3<br>c-MET positive non-small cell lung cancer/Phase 1/Phase 2<br>cancer/Phase 2<br>cancer/Phase 3<br>carcinoma/Phase 3<br>central nervous system tumor/Phase 1<br>central nervous system tumor/Phase 1/Phase 2<br>central nervous system tumor/Phase 2<br>cervical cancer/Phase 2<br>childhood anaplastic astrocytoma/Phase 1/Phase 2

© 2000-2014 QIAGEN. All rights reserved.

childhood anaplastic oligodendroglioma/Phase 1/Phase 2
childhood brain stem glioma/Phase 1/Phase 2
childhood giant cell glioblastoma/Phase 1/Phase 2
childhood gliomatosis cerebri/Phase 1/Phase 2
childhood gliosarcoma/Phase 1/Phase 2
childhood solid tumor/Phase 1
colon adenocarcinoma/Phase 2
colon cancer/Phase 2
colorectal cancer/Phase 1
colorectal cancer/Phase 1/Phase 2
colorectal cancer/Phase 2
colorectal cancer/Phase 2/Phase 3
EGFR (L858R) positive lung adenocarcinoma/Phase 3
EGFR activating mutation positive EGFR resistance mutation negative nonsquamous non-small-cell lung cancer/Phase 2/Phase 3
EGFR activating mutation positive MET positive nonsquamous non-small cell lung cancer/Phase 1/Phase 2
EGFR activating mutation positive squamous non-small cell lung cancer/Phase 2
EGFR exon 18 mutation positive nonsquamous non-small cell lung cancer/Phase 2
EGFR exon 19 deletion positive EGFR T790M mutation negative non-small cell lung cancer/Phase 1
EGFR exon 19 deletion positive lung adenocarcinoma/Phase 3
EGFR exon 19 deletion positive non-small cell lung cancer/Phase 1
EGFR exon 19 deletion positive non-small cell lung cancer/Phase 2
EGFR exon 19 deletion positive non-small cell lung cancer/Phase 2/Phase 3
EGFR exon 19 deletion positive non-small cell lung cancer/Phase 3
EGFR exon 19 mutation positive non-small cell lung cancer/Phase 3
EGFR exon 19 mutation positive nonsquamous non-small cell lung cancer/Phase 2
EGFR exon 21 mutation positive non-small cell lung cancer/Phase 2
EGFR exon 21 mutation positive non-small cell lung cancer/Phase 3
EGFR exon 21 mutation positive nonsquamous non-small cell lung cancer/Phase 2
EGFR exon 21 point mutation positive non-small cell lung cancer/Phase 2
EGFR expressing non-small cell lung cancer/Phase 1
EGFR G719X mutation positive non-small cell lung cancer/Phase 1
EGFR L858 mutation positive non-small cell lung cancer/Phase 2
EGFR L858 mutation positive non-small cell lung cancer/Phase 2/Phase 3
EGFR L858R mutation positive EGFR T790M mutation negative non-small cell lung cancer/Phase 1
EGFR L858R mutation positive non-small cell lung cancer/Phase 1
EGFR L858R mutation positive non-small cell lung cancer/Phase 2
EGFR L858R mutation positive non-small cell lung cancer/Phase 3
EGFR L861Q mutation positive non-small cell lung cancer/Phase 1
EGFR mutation negative non squamous non-small cell lung cancer/Phase 2
EGFR mutation negative non squamous non-small cell lung cancer/Phase 2/Phase 3
EGFR mutation negative non-small cell lung cancer/Phase 2
EGFR mutation positive adenocarcinoma of the lung/Unspecified phase
EGFR mutation positive non small cell lung carcinoma/Phase 1
EGFR mutation positive non small cell lung carcinoma/Phase 1/Phase 2
EGFR mutation positive non-small cell lung cancer/Phase 1
EGFR mutation positive non-small cell lung cancer/Phase 2
EGFR mutation positive non-small cell lung cancer/Phase 2/Phase 3
EGFR sensitizing mutation positive lung adenocarcinoma/Phase 4

© 2000-2014 QIAGEN. All rights reserved.

EGFR sensitizing mutation positive MET positive non-small cell lung cancer/Phase 1
EGFR sensitizing mutation positive non-small cell lung cancer/Phase 1
EGFR sensitizing mutation positive non-small cell lung cancer/Phase 2
EGFR sensitizing mutation positive non-small cell lung cancer/Phase 4
EGFR T790M positive nonsquamous non-small cell lung cancer/Phase 2/Phase 3
epithelioid mesothelioma/Phase 2
esophageal cancer/Phase 1
esophageal cancer/Phase 1/Phase 2
esophageal cancer/Phase 2
estrogen receptor positive breast cancer/Phase 2
fallopian tube cancer/Phase 1/Phase 2
fallopian tube cancer/Phase 2
follicular thyroid cancer/Unspecified phase
gastric cancer/Phase 2
gastrinoma/Phase 2
gastrointestinal tract cancer/Unspecified phase
glioblastoma cancer/Phase 2
glucagonoma/Phase 2
head and neck cancer/Phase 1
head and neck cancer/Phase 1/Phase 2
head and neck cancer/Phase 2
head and neck cancer/Phase 3
head and neck cancer/Phase 4
head and neck squamous cell carcinoma/Phase 3
head and neck tumor/Phase 1/Phase 2
head and neck tumor/Phase 2
hypopharyngeal squamous cell carcinoma/Phase 1
hypopharyngeal squamous cell carcinoma/Unspecified phase
insular thyroid cancer/Unspecified phase
insulinoma/Phase 2
large cell lung cancer/Phase 2
large cell lung cancer/Phase 3
laryngeal squamous cell carcinoma/Phase 1
laryngeal squamous cell carcinoma/Unspecified phase
laryngeal verrucous carcinoma/Unspecified phase
liver cancer/Phase 2
locally advanced EGFR mutation positive non-small cell lung cancer/Phase 1
locally advanced EGFR mutation positive non-small cell lung cancer/Phase 2
locally advanced non small cell lung cancer/Approved
locally advanced non small cell lung cancer/Phase 1
lung adenocarcinoma/Unspecified phase
lung adenosquamous cancer/Phase 2
lung adenosquamous cancer/Phase 3
lung cancer/Phase 1
lung cancer/Phase 1/Phase 2
lung cancer/Phase 2
lung cancer/Phase 3
lung cancer/Unspecified phase
lung tumor/Phase 1/Phase 2
lung tumor/Phase 2
lymphocytic cancer/Phase 1
male breast cancer/Phase 2
malignant mesothelioma/Phase 2
mammary tumor/Phase 2
mesothelioma/Phase 2
metastasis/Phase 2
metastasis/Phase 3
metastatic breast cancer/Phase 2
metastatic colorectal cancer/Phase 2
metastatic non-small-cell lung cancer/Approved
metastatic non-small-cell lung cancer/Phase 1
metastatic non-small-cell lung cancer/Phase 1/Phase 2
metastatic non-small-cell lung cancer/Phase 2
metastatic non-small-cell lung cancer/Phase 3
metastatic non-small-cell lung cancer/Unspecified phase
mucinous adenocarcinoma of colon/Phase 2
mucinous adenocarcinoma of rectum/Phase 2
nasopharyngeal carcinoma/Phase

© 2000-2014 QIAGEN. All rights reserved.

1/Phase 2
neoplasia/Phase 1
neoplasia/Phase 3
non small cell lung adenocarcinoma/Phase 2
non small cell lung adenocarcinoma/Phase 3
non-small cell lung cancer with activating EGFR mutations/Phase 1
non-small cell lung cancer with activating EGFR mutations/Phase 2
non-small cell lung cancer with activating EGFR mutations/Phase 3
non-small cell lung cancer/Phase 1
non-small cell lung cancer/Phase 1/Phase 2
non-small cell lung cancer/Phase 2
non-small cell lung cancer/Phase 2/Phase 3
non-small cell lung cancer/Phase 3
non-small cell lung cancer/Phase 4
non-small cell lung cancer/Unspecified phase
non-small-cell lung carcinoma/Approved
non-squamous non-small cell lung cancer/Phase 2
oropharyngeal squamous-cell carcinoma/Unspecified phase
ovarian cancer/Phase 1/Phase 2
ovarian cancer/Phase 2
pancreatic cancer/Phase 1/Phase 2
pancreatic carcinoma/Phase 2
papillary thyroid cancer/Unspecified phase
parathyroid cancer/Unspecified phase
pediatric glioblastoma/Phase 1/Phase 2
peritoneal carcinoma/Phase 2
peritoneal cavity cancer/Phase 2
peritoneal tumor/Phase 1/Phase 2
pituitary ACTH hypersecretion/Phase 2
primary peritoneal cancer/Phase 2
progesterone receptor-positive breast cancer/Phase 2
prostate cancer/Phase 1/Phase 2
prostate cancer/Phase 2
rectal adenocarcinoma/Phase 2
rectum cancer/Phase 2
recurrent breast cancer/Phase 2
recurrent EGFR exon 19 deletion positive non-small cell lung cancer/Phase 1
recurrent EGFR exon 19 deletion positive non-small cell lung cancer/Phase 2
recurrent EGFR L858R mutation positive non-small cell lung cancer/Phase 1
recurrent islet cell carcinoma/Phase 2
recurrent laryngeal squamous cell carcinoma/Unspecified phase
recurrent rectal cancer/Phase 2
renal cancer/Phase 2
renal-cell carcinoma/Phase 1/Phase 2
salivary gland cancer/Phase 2
salivary gland cancer/Unspecified phase
sarcoma/Phase 2
signet ring adenocarcinoma of the colon/Phase 2
signet ring adenocarcinoma of the rectum/Phase 2
skin cancer/Phase 2
skin squamous cell carcinoma/Phase 2
small cell lung cancer/Phase 2
solid tumor/Phase 1
solid tumor/Phase 2
somatostatinoma/Phase 2
squamous cell carcinoma of nasopharynx/Unspecified phase
squamous cell carcinoma/Phase 1
squamous cell carcinoma/Phase 2
squamous cell lung cancer/Phase 2
squamous cell lung cancer/Phase 3
squamous cell tumor/Phase 2
stage III oropharyngeal squamous cell carcinoma/Phase 1
stage IV lung adenocarcinoma/Phase 3
stage IV oropharyngeal squamous cell carcinoma/Phase 1
stage IVA oropharyngeal squamous cell carcinoma/Unspecified phase
stage IVB oropharyngeal squamous cell carcinoma/Unspecified phase
stage IVC oropharyngeal squamous cell carcinoma/Unspecified phase
thyroid cancer/Unspecified phase

© 2000-2014 QIAGEN. All rights reserved.

				tongue cancer/Unspecified phase<br>triple-negative EGFR positive breast cancer/Phase 2<br>tumor/Phase 1<br>undifferentiated nasopharyngeal carcinoma/Unspecified phase<br>urethral cancer/Phase 2
gefitinib	EGFR	antagonist	Iressa, Irressat	activating EGFR mutation positive non small cell lung carcinoma/Phase 2<br>acute myeloid leukemia/Phase 2<br>adult solid tumor/Phase 1<br>advanced non-small-cell lung cancer/Phase 2<br>anaplastic thyroid cancer/Unspecified phase<br>bladder cancer/Phase 2<br>bladder cancer/Phase 3<br>brain metastasis associated with EGFR mutation positive non-small cell lung cancer/Phase 2<br>brain metastasis/Phase 2<br>brain metastasis/Unspecified phase<br>brain tumor/Phase 2<br>breast cancer/Phase 1/Phase 2<br>breast cancer/Phase 2<br>breast cancer/Phase 3<br>c-MET positive non-small cell lung cancer/Phase 1/Phase 2<br>cancer/Phase 2<br>cancer/Phase 3<br>carcinoma/Phase 3<br>central nervous system tumor/Phase 1<br>central nervous system tumor/Phase 1/Phase 2<br>central nervous system tumor/Phase 2<br>cervical cancer/Phase 2<br>childhood anaplastic astrocytoma/Phase 1/Phase 2<br>childhood anaplastic oligodendroglioma/Phase 1/Phase 2<br>childhood brain stem glioma/Phase 1/Phase 2<br>childhood giant cell glioblastoma/Phase 1/Phase 2<br>childhood gliomatosis cerebri/Phase 1/Phase 2<br>childhood gliosarcoma/Phase 1/Phase 2<br>childhood solid tumor/Phase 1<br>colon adenocarcinoma/Phase 2<br>colon cancer/Phase 2<br>colorectal cancer/Phase 1<br>colorectal cancer/Phase 1/Phase 2<br>colorectal cancer/Phase 2<br>colorectal cancer/Phase 2/Phase 3<br>EGFR (L858R) positive lung adenocarcinoma/Phase 3<br>EGFR activating mutation positive EGFR resistance mutation negative nonsquamous non-small-cell lung cancer/Phase 2/Phase 3<br>EGFR activating mutation positive MET positive nonsquamous non-small cell lung cancer/Phase 1/Phase 2<br>EGFR activating mutation positive squamous non-small cell lung cancer/Phase 2<br>EGFR exon 18 mutation positive nonsquamous non-small cell lung cancer/Phase 2<br>EGFR exon 19 deletion positive EGFR T790M mutation negative non-small cell lung cancer/Phase 1<br>EGFR exon 19 deletion positive lung adenocarcinoma/Phase 3<br>EGFR exon 19 deletion positive non-small cell lung cancer/Phase 1<br>EGFR exon 19 deletion positive non-small cell lung cancer/Phase 2<br>EGFR exon 19 deletion positive non-small cell lung cancer/Phase 2/Phase 3<br>EGFR exon 19 deletion positive non-small cell lung cancer/Phase 3<br>EGFR exon 19 mutation positive non-small cell lung cancer/Phase 3<br>EGFR exon 19 mutation positive nonsquamous non-small cell lung cancer/Phase 2<br>EGFR exon 21 mutation positive non-small cell lung cancer/Phase 2<br>EGFR exon 21 mutation positive non-small cell lung cancer/Phase 3<br>EGFR exon 21 mutation positive nonsquamous non-small cell lung cancer/Phase 2<br>EGFR exon 21 point mutation positive non-small cell lung cancer/Phase 2<br>EGFR expressing non-small cell lung cancer/Phase 1

© 2000-2014 QIAGEN. All rights reserved.

EGFR G719X mutation positive non-small cell lung cancer/Phase 1
EGFR L858 mutation positive non-small cell lung cancer/Phase 2
EGFR L858 mutation positive non-small cell lung cancer/Phase 2/Phase 3
EGFR L858R mutation positive EGFR T790M mutation negative non-small cell lung cancer/Phase 1
EGFR L858R mutation positive non-small cell lung cancer/Phase 1
EGFR L858R mutation positive non-small cell lung cancer/Phase 2
EGFR L858R mutation positive non-small cell lung cancer/Phase 3
EGFR L861Q mutation positive non-small cell lung cancer/Phase 1
EGFR mutation negative non squamous non-small cell lung cancer/Phase 2
EGFR mutation negative non squamous non-small cell lung cancer/Phase 2/Phase 3
EGFR mutation negative non-small cell lung cancer/Phase 2
EGFR mutation positive adenocarcinoma of the lung/Unspecified phase
EGFR mutation positive non small cell lung carcinoma/Phase 1
EGFR mutation positive non small cell lung carcinoma/Phase 1/Phase 2
EGFR mutation positive non-small cell lung cancer/Phase 1
EGFR mutation positive non-small cell lung cancer/Phase 2
EGFR mutation positive non-small cell lung cancer/Phase 2/Phase 3
EGFR sensitizing mutation positive lung adenocarcinoma/Phase 4
EGFR sensitizing mutation positive MET positive non-small cell lung cancer/Phase 1
EGFR sensitizing mutation positive non-small cell lung cancer/Phase 1
EGFR sensitizing mutation positive non-small cell lung cancer/Phase 2
EGFR sensitizing mutation positive non-small cell lung cancer/Phase 4
EGFR T790M positive nonsquamous non-small cell lung cancer/Phase 2/Phase 3
epithelioid mesothelioma/Phase 2
esophageal cancer/Phase 1
esophageal cancer/Phase 1/Phase 2
esophageal cancer/Phase 2
estrogen receptor positive breast cancer/Phase 2
fallopian tube cancer/Phase 1/Phase 2
fallopian tube cancer/Phase 2
follicular thyroid cancer/Unspecified phase
gastric cancer/Phase 2
gastrinoma/Phase 2
gastrointestinal tract cancer/Unspecified phase
glioblastoma cancer/Phase 2
glucagonoma/Phase 2
head and neck cancer/Phase 1
head and neck cancer/Phase 1/Phase 2
head and neck cancer/Phase 2
head and neck cancer/Phase 3
head and neck cancer/Phase 4
head and neck squamous cell carcinoma/Phase 3
head and neck tumor/Phase 1/Phase 2
head and neck tumor/Phase 2
hypopharyngeal squamous cell carcinoma/Phase 1
hypopharyngeal squamous cell carcinoma/Unspecified phase
insular thyroid cancer/Unspecified phase
insulinoma/Phase 2
large cell lung cancer/Phase 2
large cell lung cancer/Phase 3
laryngeal squamous cell carcinoma/Phase 1
laryngeal squamous cell carcinoma/Unspecified phase
laryngeal verrucous carcinoma/Unspecified phase
liver cancer/Phase 2
locally advanced EGFR mutation positive non-small cell lung cancer/Phase 1
locally advanced EGFR mutation positive non-small cell lung cancer/Phase 2
locally advanced non small cell lung cancer/Approved

© 2000-2014 QIAGEN. All rights reserved.

locally advanced non small cell lung cancer/Phase 1
lung adenocarcinoma/Unspecified phase
lung adenosquamous cancer/Phase 2
lung adenosquamous cancer/Phase 3
lung cancer/Phase 1
lung cancer/Phase 1/Phase 2
lung cancer/Phase 2
lung cancer/Phase 3
lung cancer/Unspecified phase
lung tumor/Phase 1/Phase 2
lung tumor/Phase 2
lymphocytic cancer/Phase 1
male breast cancer/Phase 2
malignant mesothelioma/Phase 2
mammary tumor/Phase 2
mesothelioma/Phase 2
metastasis/Phase 2
metastasis/Phase 3
metastatic breast cancer/Phase 2
metastatic colorectal cancer/Phase 2
metastatic non-small-cell lung cancer/Approved
metastatic non-small-cell lung cancer/Phase 1
metastatic non-small-cell lung cancer/Phase 1/Phase 2
metastatic non-small-cell lung cancer/Phase 2
metastatic non-small-cell lung cancer/Phase 3
metastatic non-small-cell lung cancer/Unspecified phase
mucinous adenocarcinoma of colon/Phase 2
mucinous adenocarcinoma of rectum/Phase 2
nasopharyngeal carcinoma/Phase 1/Phase 2
neoplasia/Phase 1
neoplasia/Phase 3
non small cell lung adenocarcinoma/Phase 2
non small cell lung adenocarcinoma/Phase 3
non-small cell lung cancer with activating EGFR mutations/Phase 1
non-small cell lung cancer with activating EGFR mutations/Phase 2
non-small cell lung cancer with activating EGFR mutations/Phase 3
non-small cell lung cancer/Phase 1
non-small cell lung cancer/Phase 1/Phase 2
non-small cell lung cancer/Phase 2
non-small cell lung cancer/Phase 2/Phase 3
non-small cell lung cancer/Phase 3
non-small cell lung cancer/Phase 4
non-small cell lung cancer/Unspecified phase
non-small-cell lung carcinoma/Approved
non-squamous non-small cell lung cancer/Phase 2
oropharyngeal squamous-cell carcinoma/Unspecified phase
ovarian cancer/Phase 1/Phase 2
ovarian cancer/Phase 2
pancreatic cancer/Phase 1/Phase 2
pancreatic carcinoma/Phase 2
papillary thyroid cancer/Unspecified phase
parathyroid cancer/Unspecified phase
pediatric glioblastoma/Phase 1/Phase 2
peritoneal carcinoma/Phase 2
peritoneal cavity cancer/Phase 2
peritoneal tumor/Phase 1/Phase 2
pituitary ACTH hypersecretion/Phase 2
primary peritoneal cancer/Phase 2
progesterone receptor-positive breast cancer/Phase 2
prostate cancer/Phase 1/Phase 2
prostate cancer/Phase 2
rectal adenocarcinoma/Phase 2
rectum cancer/Phase 2
recurrent breast cancer/Phase 2
recurrent EGFR exon 19 deletion positive non-small cell lung cancer/Phase 1
recurrent EGFR exon 19 deletion positive non-small cell lung cancer/Phase 2
recurrent EGFR L858R mutation positive non-small cell lung cancer/Phase 1
recurrent islet cell carcinoma/Phase 2
recurrent laryngeal squamous cell carcinoma/Unspecified phase

© 2000-2014 QIAGEN. All rights reserved.

				recurrent rectal cancer/Phase 2<br>renal cancer/Phase 2<br>renal-cell carcinoma/Phase 1/Phase 2<br>salivary gland cancer/Phase 2<br>salivary gland cancer/Unspecified phase<br>sarcoma/Phase 2<br>signet ring adenocarcinoma of the colon/Phase 2<br>signet ring adenocarcinoma of the rectum/Phase 2<br>skin cancer/Phase 2<br>skin squamous cell carcinoma/Phase 2<br>small cell lung cancer/Phase 2<br>solid tumor/Phase 1<br>solid tumor/Phase 2<br>somatostatinoma/Phase 2<br>squamous cell carcinoma of nasopharynx/Unspecified phase<br>squamous cell carcinoma/Phase 1<br>squamous cell carcinoma/Phase 2<br>squamous cell lung cancer/Phase 2<br>squamous cell lung cancer/Phase 3<br>squamous cell tumor/Phase 2<br>stage III oropharyngeal squamous cell carcinoma/Phase 1<br>stage IV lung adenocarcinoma/Phase 3<br>stage IV oropharyngeal squamous cell carcinoma/Phase 1<br>stage IVA oropharyngeal squamous cell carcinoma/Unspecified phase<br>stage IVB oropharyngeal squamous cell carcinoma/Unspecified phase<br>stage IVC oropharyngeal squamous cell carcinoma/Unspecified phase<br>thyroid cancer/Unspecified phase<br>tongue cancer/Unspecified phase<br>triple-negative EGFR positive breast cancer/Phase 2<br>tumor/Phase 1<br>undifferentiated nasopharyngeal carcinoma/Unspecified phase<br>urethral cancer/Phase 2
gemcitabine	RRM2	inhibitor	DDFC, DFDC, Gemcin, Gemcitabina [INN-Spanish], Gemcitabinum [INN-Latin], Gemtro, Gemzar, GEO	adenocarcinoma/Phase 1<br>adenocarcinoma/Phase 1/Phase 2<br>adenocarcinoma/Phase 2<br>adenocarcinoma/Phase 3<br>adult Burkitt lymphoma/Phase 1<br>adult Burkitt lymphoma/Phase 2<br>adult diffuse large-cell lymphoma/Phase 1<br>adult diffuse mixed-cell lymphoma/Phase 1<br>adult diffuse mixed-cell lymphoma/Phase 2<br>adult Hodgkin lymphoma/Phase 1<br>adult Hodgkin lymphoma/Phase 1/Phase 2<br>adult Hodgkin lymphoma/Phase 2<br>adult lymphoblastic lymphoma/Phase 1<br>adult lymphoblastic lymphoma/Phase 2<br>adult solid tumor/Phase 1<br>adult solid tumor/Phase 1/Phase 2<br>adult solid tumor/Phase 2<br>adult T cell leukemia/Phase 1<br>adult T cell leukemia/Phase 2<br>advanced breast cancer/Phase 3<br>advanced cancer/Phase 1<br>advanced cancer/Phase 1/Phase 2<br>advanced colorectal cancer/Phase 2<br>advanced non-small-cell lung cancer/Phase 2<br>advanced non-small-cell lung cancer/Phase 2/Phase 3<br>advanced non-small-cell lung cancer/Phase 3<br>advanced soft tissue sarcoma/Phase 1/Phase 2<br>advanced solid tumor/Phase 1<br>advanced solid tumor/Phase 3<br>aggressive non-Hodgkin lymphoma/Phase 1<br>aggressive non-Hodgkin lymphoma/Phase 2<br>ALK fusion negative anaplastic large cell lymphoma/Phase 2<br>ALK fusion negative EGFR TK activating negative non-small cell lung cancer/Phase 3<br>ALK fusion negative squamous cell lung cancer/Phase 2<br>ALK fusion positive solid tumor/Phase 1<br>ALK mutation negative acute myeloid leukemia/Phase 2<br>ALK translocation negative EGFR mutation negative PD-L1 positive

© 2000-2014 QIAGEN. All rights reserved.

non-small cell lung cancer/Phase 3
ALK translocation negative EGFR sensitizing mutation negative PD-L1 expression positive non-small cell lung cancer/Phase 3
ALK translocation negative EGFR sensitizing mutation negative PD-L1 expression positive non-small cell lung carcinoma/Phase 3
ALK translocation positive non-small cell lung cancer/Phase 2
ALK-EML4 fusion negative non-small cell lung cancer/Phase 2
ALK-EML4 mutation negative EGFR mutation negative lung adenocarcinoma/Phase 2
ALK-EML4 translocation negative non-small cell lung adenocarcinoma/Phase 2
ALK/EML4 fusion negative EGFR mutation negative non-small cell lung cancer/Phase 2
alveolar soft part sarcoma/Phase 2
ampullary cancer/Phase 2
androgen receptor positive pancreatic adenocarcinoma/Phase 1
androgen receptor positive pancreatic cancer/Phase 1
angiocentric T-cell lymphoma/Phase 2
angioimmunoblastic T-cell lymphoma/Phase 1
angioimmunoblastic T-cell lymphoma/Phase 2
angiosarcoma/Phase 2
anxiety disorder/Phase 2
B-cell non-Hodgkin's disease/Phase 1
B-cell non-Hodgkin's disease/Phase 1/Phase 2
B-cell non-Hodgkin's disease/Phase 2
bile duct cancer/Phase 1
bile duct cancer/Phase 2
biliary tract cancer/Phase 1
biliary tract cancer/Phase 1/Phase 2
biliary tract cancer/Phase 2
biliary tract cancer/Phase 3
biliary tract carcinoma/Phase 1
biliary tract carcinoma/Phase 1/Phase 2
biliary tract carcinoma/Phase 2
biliary tract disease/Phase 2
biliary tract tumor/Phase 1/Phase 2
biliary tract tumor/Phase 2
biliary tract tumor/Phase 2/Phase 3
bladder cancer/Phase 1
bladder cancer/Phase 1/Phase 2
bladder cancer/Phase 2
bladder cancer/Phase 2/Phase 3
bladder cancer/Phase 3
bladder cancer/Unspecified phase
bladder carcinoma/Phase 1
bladder carcinoma/Phase 1/Phase 2
bladder carcinoma/Phase 2
bladder carcinoma/Phase 3
bladder squamous cell carcinoma/Phase 2
bladder tumor/Phase 2
bladder tumor/Phase 3
bone sarcoma/Phase 1
BRCA1 mutation positive pancreatic adenocarcinoma/Phase 2
BRCA2 mutation positive pancreatic adenocarcinoma/Phase 2
breast cancer/Phase 1
breast cancer/Phase 1/Phase 2
breast cancer/Phase 2
breast cancer/Phase 3
breast carcinoma/Phase 1/Phase 2
C-KIT expressing malignant peritooneal mesothelioma/Phase 2
C-KIT expressing malignant pleural mesothelioma/Phase 2
cachexia/Phase 2
cancer/Phase 1
cancer/Phase 1/Phase 2
cancer/Phase 2
cancer/Phase 3
carcinoma of ampulla of Vater/Phase 2
carcinoma/Phase 1
carcinoma/Phase 2
carcinoma/Phase 3
central nervous system tumor/Phase 1/Phase 2
central nervous system tumor/Phase 2
cervical adenocarcinoma/Phase 1
cervical adenocarcinoma/Phase 3
cervical adenosquamous carcinoma/Phase 1

© 2000-2014 QIAGEN. All rights reserved.

cervical adenosquamous carcinoma/Phase 3
cervical cancer/Phase 1
cervical cancer/Phase 1/Phase 2
cervical cancer/Phase 2
cervical cancer/Phase 3
cervical carcinoma/Phase 1
cervical small cell carcinoma/Phase 1
cervical squamous cell carcinoma/Phase 1
cervical squamous cell carcinoma/Phase 3
chemotherapy induced thrombocytopenia/Phase 2
childhood solid tumor/Phase 1
cholangiocarcinoma/Phase 0
cholangiocarcinoma/Phase 1
cholangiocarcinoma/Phase 1/Phase 2
cholangiocarcinoma/Phase 2
cholestasis/Phase 1
chronic B-cell leukemia/Phase 1
chronic B-cell leukemia/Phase 2
cKIT expression positive PDGFR-beta expression positive malignant peritoneal mesothelioma/Phase 2
cKIT expression positive PDGFR-beta expression positive malignant pleural mesothelioma/Phase 2
clear-cell adenocarcinoma/Phase 3
clear-cell ovarian carcinoma/Phase 3
colon cancer/Phase 1/Phase 2
colon cancer/Phase 2
colorectal cancer/Phase 1
colorectal cancer/Phase 1/Phase 2
colorectal cancer/Phase 2
cutaneous B-cell lymphoma/Phase 2
cutaneous T-cell lymphoma/Phase 1
cutaneous T-cell lymphoma/Phase 2
de novo diffuse large B-cell lymphoma/Phase 3
depressive disorder/Phase 2
dermatofibrosarcoma protuberans/Phase 2
desmoplastic small round cell tumor/Phase 2
diffuse large B-cell lymphoma/Phase 1
diffuse large B-cell lymphoma/Phase 1/Phase 2
diffuse large B-cell lymphoma/Phase 2
diffuse large B-cell lymphoma/Phase 2/Phase 3
diffuse large B-cell lymphoma/Phase 3
disorder of ovary/Phase 1
disorder of pancreas/Phase 2
EGFR activating mutation negative adenocarcinoma of the lung/Phase 2
EGFR exon 19 deletion positive non-small cell lung cancer/Phase 2
EGFR exon 19 mutation positive non-small cell lung cancer/Phase 2
EGFR exon 21 mutation positive non-small cell lung cancer/Phase 2
EGFR exon 21 point mutation positive non-small cell lung cancer/Phase 2
EGFR mutation negative non squamous non-small cell lung cancer/Phase 1
EGFR mutation negative non squamous non-small cell lung cancer/Phase 3
EGFR mutation negative non-small cell lung adenocarcinoma/Phase 2
EGFR mutation negative non-small cell lung cancer/Phase 2
EGFR mutation negative non-small cell lung cancer/Phase 3
EGFR mutation negative squamous non-small cell lung cancer/Phase 1
EGFR mutation negative squamous non-small cell lung cancer/Phase 2
EGFR mutation positive EGFR exon 20 insertion activating mutation negative non-small cell lung cancer/Phase 3
EGFR mutation positive non-small cell lung cancer/Phase 3
EGFR sensitizing mutation positive non-squamous non-small cell lung cancer/Phase 2
endometrial adenocarcinoma/Phase 2
endometrial adenosquamous carcinoma/Phase 2
endometrial cancer/Phase 1
endometrial cancer/Phase 2
endometrial carcinoma/Phase 1

© 2000-2014 QIAGEN. All rights reserved.

endometrial carcinoma/Unspecified phase
endometrial clear cell carcinoma/Phase 2
epithelial ovarian cancer/Phase 1
epithelial ovarian cancer/Phase 1/Phase 2
epithelial ovarian cancer/Phase 2
epithelial ovarian cancer/Phase 3
epithelial ovarian cancer/Unspecified phase
epithelioid hemangioendothelioma/Phase 2
epithelioid mesothelioma/Phase 2
epithelioid sarcoma/Phase 2
esophageal cancer/Phase 1
esophageal cancer/Phase 2
exocrine pancreatic cancer/Phase 2
extragonadal germ cell tumor/Phase 1/Phase 2
extrahepatic bile duct cancer/Phase 1
extrahepatic bile duct cancer/Phase 1/Phase 2
extrahepatic bile duct cancer/Phase 2
extrahepatic bile duct cancer/Phase 2/Phase 3
extrahepatic bile duct cancer/Phase 3
extrahepatic bile duct cancer/Unspecified phase
extrahepatic cholangiocarcinoma/Phase 2
extrahepatic cholangiocarcinoma/Phase 3
extraskeletal osteosarcoma/Phase 2
fallopian tube cancer/Phase 1
fallopian tube cancer/Phase 1/Phase 2
fallopian tube cancer/Phase 2
fallopian tube cancer/Unspecified phase
fallopian tube carcinoma/Phase 2
fallopian tube carcinoma/Phase 3
fallopian tube clear cell adenocarcinoma/Phase 3
fallopian tube endometrioid adenocarcinoma/Phase 3
fallopian tube neoplasm/Phase 1
fallopian tube neoplasm/Phase 3
fallopian tube serous adenocarcinoma/Phase 3
fatigue/Phase 2
female genital neoplasm/Phase 3
fibrosarcoma/Phase 2
follicular non-Hodgkin's disease/Phase 1/Phase 2
follicular non-Hodgkin's disease/Phase 3
gall bladder adenocarcinoma/Phase 1
gall bladder adenocarcinoma/Phase 2
gall bladder carcinoma/Phase 1
gallbladder cancer/Phase 1
gallbladder cancer/Phase 1/Phase 2
gallbladder cancer/Phase 2
gallbladder cancer/Phase 2/Phase 3
gallbladder cancer/Phase 3
gallbladder neoplasia/Phase 1/Phase 2
gastric cancer/Phase 1
gastric cancer/Phase 2
germ cell tumor/Phase 2
grade 1 follicular lymphoma/Phase 1
grade 2 follicular lymphoma/Phase 1
grade 3 follicular lymphoma/Phase 1
head and neck cancer/Phase 1
head and neck cancer/Phase 1/Phase 2
head and neck cancer/Phase 2
head and neck cancer/Phase 2/Phase 3
head and neck squamous cell carcinoma/Phase 1
hepatic adenocarcinoma/Phase 2
hepatocellular carcinoma/Phase 1
hepatocellular carcinoma/Phase 2
hepatocellular carcinoma/Phase 3
hepatocellular carcinoma/Unspecified phase
HER2 negative hormone receptor negative breast cancer/Phase 1
HER2 negative hormone receptor negative breast cancer/Phase 1/Phase 2
HER2 negative hormone receptor negative breast cancer/Phase 2
HER2 negative hormone receptor

© 2000-2014 QIAGEN. All rights reserved.

negative breast cancer/Phase 2/Phase 3
HER2 negative metastatic breast cancer/Phase 2
HER2 positive invasive breast cancer/Phase 2/Phase 3
HER2-negative breast cancer/Phase 1
HER2-negative breast cancer/Phase 2
HER2-positive breast cancer/Phase 1/Phase 2
HER2-positive breast cancer/Phase 2
Hodgkin's disease/Phase 1
Hodgkin's disease/Phase 1/Phase 2
Hodgkin's disease/Phase 2
invasive breast cancer/Phase 2
Klatskin's tumor/Phase 3
KRAS mutation negative cholangiocarcinoma/Phase 2
KRAS mutation negative epithelial ovarian cancer/Phase 2
KRAS mutation negative fallopian tube cancer/Phase 2
KRAS mutation negative gall bladder carcinoma/Phase 2
KRAS mutation negative pancreatic adenocarcinoma/Phase 3
KRAS mutation negative pancreatic cancer/Phase 3
KRAS mutation negative peritoneal carcinomatosis/Phase 2
large-cell diffuse lymphoma/Phase 2
large-cell Ki-1 lymphoma/Phase 1
large-cell Ki-1 lymphoma/Phase 2
leiomyosarcoma/Phase 1
leiomyosarcoma/Phase 2
leukemia/Phase 1/Phase 2
leukemia/Phase 2
leydig cell tumor/Phase 1
liposarcoma/Phase 1
liposarcoma/Phase 2
liver cancer/Phase 1
liver cancer/Phase 1/Phase 2
liver cancer/Phase 2
liver cancer/Phase 2/Phase 3
liver cancer/Phase 3
liver cholangiocarcinoma/Phase 1
liver cholangiocarcinoma/Phase 2
liver cholangiocarcinoma/Phase 3
liver cholangiocarcinoma/Unspecified phase
liver metastasis/Phase 1/Phase 2
liver tumor/Phase 0
locally advanced breast cancer/Phase 2
locally advanced HER2 negative breast cancer/Phase 2
locally advanced KRAS mutation positive non small cell lung cancer/Phase 2
locally advanced pancreatic cancer/Approved
locally advanced pancreatic cancer/Phase 1
locally advanced pancreatic cancer/Phase 1/Phase 2
locally advanced pancreatic cancer/Phase 2
locally advanced pancreatic cancer/Phase 3
locally advanced triple negative breast cancer/Phase 1
lung cancer/Phase 1
lung cancer/Phase 1/Phase 2
lung cancer/Phase 2
lung cancer/Phase 2/Phase 3
lung cancer/Phase 3
lung carcinoma/Approved
lung tumor/Phase 1/Phase 2
lung tumor/Phase 2
lymphocytic cancer/Phase 1
lymphocytic cancer/Phase 1/Phase 2
lymphocytic cancer/Phase 2
lymphocytic cancer/Phase 3
lymphoma/Phase 1
lymphoproliferative disorder/Phase 1
male breast cancer/Phase 2
male genital neoplasm/Phase 2
malignant cutaneous melanoma/Phase 1
malignant fibrous histiocytoma/Phase 2
malignant gastrointestinal neoplasm/Phase 1
malignant gastrointestinal stromal tumor/Phase 2
malignant hemangiopericytoma/Phase 2
malignant mesothelioma/Phase 2
malignant pleural effusion/Phase 1

© 2000-2014 QIAGEN. All rights reserved.

malignant pleural effusion/Phase 2
malignant pleural mesothelioma/Phase 1
malignant solid tumor/Phase 1
malignant solid tumor/Phase 2
malignant tumor/Phase 0
malignant tumor/Phase 1
malignant tumor/Phase 1/Phase 2
malignant tumor/Phase 3
malignant urothelial neoplasm/Phase 1
malignant urothelial neoplasm/Phase 1/Phase 2
malignant urothelial neoplasm/Phase 2
mammary tumor/Phase 2
mammary tumor/Phase 3
mantle cell lymphoma/Phase 1
marginal zone cell lymphoma/Phase 1
marginal zone cell lymphoma/Phase 2
medulloblastoma/Phase 2
meningitis/Phase 1
mesenchymoma/Phase 2
mesothelioma/Phase 2
metastasis/Phase 1
metastasis/Phase 1/Phase 2
metastasis/Phase 2
metastasis/Phase 2/Phase 3
metastasis/Phase 3
metastatic adenocarcinoma/Phase 1
metastatic bone cancer/Phase 3
metastatic breast cancer/Phase 1
metastatic breast cancer/Phase 1/Phase 2
metastatic breast cancer/Phase 2
metastatic breast cancer/Phase 3
metastatic colorectal adenocarcinoma/Phase 2
metastatic colorectal cancer/Phase 2
metastatic HER2 positive invasive breast cancer/Phase 2/Phase 3
metastatic KRAS mutation positive non-small-cell lung cancer/Phase 2
metastatic leiomyosarcoma/Phase 2
metastatic non-small-cell lung cancer/Phase 1
metastatic non-small-cell lung cancer/Phase 2
metastatic non-small-cell lung cancer/Phase 3
metastatic non-small-cell lung cancer/Unspecified phase
metastatic pancreatic adenocarcinoma/Phase 0
metastatic pancreatic adenocarcinoma/Phase 1
metastatic pancreatic adenocarcinoma/Phase 1/Phase 2
metastatic pancreatic adenocarcinoma/Phase 2
metastatic pancreatic adenocarcinoma/Phase 3
metastatic pancreatic cancer/Approved
metastatic pancreatic cancer/Phase 1
metastatic pancreatic cancer/Phase 1/Phase 2
metastatic pancreatic cancer/Phase 2
metastatic pancreatic cancer/Phase 3
metastatic pancreatic cancer/Unspecified phase
metastatic pancreatic ductal adenocarcinoma/Phase 2
metastatic renal cell carcinoma/Phase 2
metastatic sarcoma/Phase 1/Phase 2
metastatic sarcoma/Phase 2
metastatic soft tissue sarcoma/Phase 2
metastatic solid tumor/Phase 1
metastatic triple negative breast cancer/Phase 1
metastatic triple negative breast cancer/Phase 2/Phase 3
metastatic uterine leiomyosarcoma/Phase 2
mucinous adenocarcinoma/Phase 3
multiple myeloma/Phase 1/Phase 2
multiple myeloma/Phase 2
muscle invasive bladder carcinoma/Phase 2
myeloproliferative disorder/Phase 2
nasopharyngeal cancer/Phase 2
nasopharyngeal carcinoma/Phase 2
nasopharyngeal carcinoma/Phase

© 2000-2014 QIAGEN. All rights reserved.

3
nasopharyngeal tumor/Phase 2
nasopharyngeal tumor/Phase 3
nausea/Unspecified phase
neoplasia/Phase 3
neuroblastoma/Phase 2
neurofibrosarcoma/Phase 2
non-Hodgkin's disease/Phase 1
non-Hodgkin's disease/Phase 2
non-Hodgkin's disease/Phase 3
non-small cell lung cancer/Phase 1
non-small cell lung cancer/Phase 1/Phase 2
non-small cell lung cancer/Phase 2
non-small cell lung cancer/Phase 2/Phase 3
non-small cell lung cancer/Phase 3
non-small cell lung cancer/Phase 4
non-small cell lung cancer/Unspecified phase
non-squamous non-small cell lung cancer/Phase 2
non-squamous non-small cell lung cancer/Phase 3
occult primary carcinoma/Phase 1
occult primary carcinoma/Phase 2
occult primary tumor/Phase 2
occult primary tumor/Phase 3
ocular melanoma/Phase 2
osteosarcoma/Phase 2
osteosarcoma/Unspecified phase
ovarian cancer/Phase 1
ovarian cancer/Phase 1/Phase 2
ovarian cancer/Phase 2
ovarian cancer/Phase 3
ovarian endometrioid carcinoma/Phase 3
ovarian germ cell tumor/Phase 1
ovarian sarcoma/Phase 1
ovarian serous tumor/Phase 2
ovarian serous tumor/Phase 3
ovarian transitional cell carcinoma/Phase 2
ovarian transitional cell carcinoma/Phase 3
ovarian tumor/Phase 1
ovarian tumor/Phase 1/Phase 2
ovarian tumor/Phase 2
ovarian tumor/Phase 3
pain/Phase 2
PALB2 mutation positive pancreatic adenocarcinoma/Phase 2
pancreatic acinar cell carcinoma/Phase 1
pancreatic acinar cell carcinoma/Phase 1/Phase 2
pancreatic acinar cell carcinoma/Phase 2/Phase 3
pancreatic adenocarcinoma/Phase 1
pancreatic adenocarcinoma/Phase 1/Phase 2
pancreatic adenocarcinoma/Phase 2
pancreatic adenocarcinoma/Phase 2/Phase 3
pancreatic adenocarcinoma/Phase 3
pancreatic cancer/Phase 1
pancreatic cancer/Phase 1/Phase 2
pancreatic cancer/Phase 2
pancreatic cancer/Phase 2/Phase 3
pancreatic cancer/Phase 3
pancreatic cancer/Unspecified phase
pancreatic carcinoma/Approved
pancreatic carcinoma/Phase 1
pancreatic carcinoma/Phase 1/Phase 2
pancreatic carcinoma/Phase 2
pancreatic ductal adenocarcinoma/Phase 1
pancreatic ductal adenocarcinoma/Phase 1/Phase 2
pancreatic ductal adenocarcinoma/Phase 2
pancreatic ductal adenocarcinoma/Phase 2/Phase 3
pancreatic ductal carcinoma/Phase 2
pancreatic ductal carcinoma/Phase 3
pancreatic neoplasia/Phase 1
pancreatic neoplasia/Phase 1/Phase 2
pancreatic neoplasia/Phase 2
pancreatic neoplasia/Phase 3
PDGFR-beta expression positive malignant peritoneal mesothelioma/Phase 2
PDGFR-beta expression positive malignant pleural mesothelioma/Phase 2
pelvic tumor/Phase 3
peripheral T-cell lymphoma/Phase 1
peripheral T-cell lymphoma/Phase

© 2000-2014 QIAGEN. All rights reserved.

1/Phase 2
peripheral T-cell lymphoma/Phase 2
peripheral T-cell lymphoma/Phase 3
peritoneal carcinoma/Phase 2
peritoneal cavity cancer/Phase 1/Phase 2
peritoneal cavity cancer/Phase 2
peritoneal tumor/Phase 2
peritoneal tumor/Phase 3
platinum resistant TP53 mutation positive epithelial ovarian carcinoma/Phase 2
platinum resistant TP53 mutation positive fallopian tube carcinoma/Phase 2
platinum resistant TP53 mutation positive peritoneal carcinoma/Phase 2
platinum resistant TP53 mutation positive serous ovarian cancer/Phase 2
primary liver cancer/Phase 1
primary peritoneal cancer/Phase 1
primary peritoneal cancer/Phase 1/Phase 2
primary peritoneal cancer/Phase 2
primary peritoneal cancer/Phase 3
primary peritoneal cancer/Unspecified phase
primary peritoneal serous adenocarcinoma/Phase 2
primary peritoneal serous adenocarcinoma/Phase 3
primary peritoneal tumor/Phase 1
primary recurrent peritoneal carcinoma/Phase 2
primary recurrent peritoneal carcinoma/Phase 3
prostate cancer/Phase 1
prostate cancer/Phase 1/Phase 2
prostate cancer/Phase 2
prostate cancer/Phase 3
prostatic carcinoma/Phase 3
pseudomyxoma peritonei/Phase 1
rectal adenocarcinoma/Phase 2
rectum cancer/Phase 1/Phase 2
recurrent adult diffuse large-cell lymphoma/Phase 1
recurrent adult diffuse large-cell lymphoma/Phase 2
recurrent breast cancer/Phase 2
recurrent cervical carcinoma/Phase 3
recurrent EGFR exon 19 deletion positive non-small cell lung cancer/Phase 2
recurrent grade 1 follicular lymphoma/Phase 1
recurrent grade 1 follicular lymphoma/Phase 2
recurrent grade 2 follicular lymphoma/Phase 1
recurrent grade 2 follicular lymphoma/Phase 2
recurrent grade 3 follicular lymphoma/Phase 1
recurrent grade 3 follicular lymphoma/Phase 2
recurrent nonsquamous non-small cell lung cancer/Phase 2
recurrent ovarian cancer/Phase 1
recurrent ovarian cancer/Phase 3
recurrent ovarian carcinoma/Phase 1
recurrent ovarian carcinoma/Phase 2
recurrent ovarian carcinoma/Phase 3
recurrent peritoneal cancer/Phase 1
recurrent rectal cancer/Phase 1/Phase 2
relapsed mantle cell lymphoma/Phase 1
relapsed mantle cell lymphoma/Phase 2
relapsed peripheral T-cell lymphoma/Phase 3
renal cancer/Phase 1
renal cancer/Phase 2
renal parenchyma cancer/Phase 1/Phase 2
renal pelvis cancer/Phase 1
renal-cell carcinoma/Phase 1/Phase 2
renal-cell carcinoma/Phase 2
renal-cell carcinoma/Phase 4
rhabdomyosarcoma/Phase 2
sarcoma/Phase 1
sarcoma/Phase 1/Phase 2
sarcoma/Phase 2
serous ovarian adenocarcinoma/Phase 3
small cell lung cancer/Phase 1
small cell lung cancer/Phase

© 2000-2014 QIAGEN. All rights reserved.

1/Phase 2
small cell lung cancer/Phase 2
small intestine cancer/Phase 1
small intestine cancer/Phase 2
small-cell carcinoma/Phase 2
soft tissue sarcoma/Phase 1
soft tissue sarcoma/Phase 1/Phase 2
soft tissue sarcoma/Phase 2
soft tissue sarcoma/Phase 3
solid tumor/Phase 1
solid tumor/Phase 1/Phase 2
solid tumor/Phase 2
solid tumor/Phase 3
splenic marginal zone B-cell lymphoma/Phase 1
splenic marginal zone B-cell lymphoma/Phase 2
squamous cell carcinoma of nasopharynx/Phase 2
squamous cell lung cancer/Phase 1
squamous cell lung cancer/Phase 1/Phase 2
squamous cell lung cancer/Phase 2
squamous cell lung cancer/Phase 3
stage 1B non-small cell lung carcinoma/Phase 3
stage 3 ovarian cancer/Phase 1
stage II breast cancer/Phase 2
stage IIB breast cancer/Phase 2
stage IV adult diffuse large cell lymphoma/Phase 1
superficial bladder cancer/Phase 2
synovial sarcoma/Phase 2
T-cell non-Hodgkin lymphoma/Phase 2
testicular cancer/Phase 2
testicular germ cell tumor/Phase 1/Phase 2
testicular germ cell tumor/Phase 2
thrombocytopenia/Phase 2
thromboembolism/Phase 3
TP53 amplification positive fallopian tube cancer/Phase 1
tp53 amplification positive ovarian cancer/Phase 1
TP53 amplification positive peritoneal cancer/Phase 1
TP53 mutation positive epithelial ovarian cancer/Phase 1
TP53 mutation positive fallopian tube cancer/Phase 1
TP53 mutation positive peritoneal cancer/Phase 1
transitional cell bladder cancer/Phase 1
transitional cell bladder cancer/Phase 1/Phase 2
transitional cell bladder cancer/Phase 2
transitional cell bladder cancer/Phase 3
transitional cell epithelial cancer/Phase 1/Phase 2
transitional cell epithelial cancer/Phase 2
transitional cell epithelial cancer/Phase 2/Phase 3
transitional cell epithelial cancer/Phase 3
triple negative breast adenocarcinoma/Phase 2/Phase 3
tumor/Phase 1
tumor/Phase 1/Phase 2
undernutrition/Unspecified phase
undifferentiated carcinoma/Phase 3
undifferentiated fallopian tube carcinoma/Phase 2
undifferentiated fallopian tube carcinoma/Phase 3
undifferentiated nasopharyngeal carcinoma/Phase 2/Phase 3
undifferentiated ovarian carcinoma/Phase 2
undifferentiated ovarian carcinoma/Phase 3
ureteric cancer/Phase 1/Phase 2
urethral cancer/Phase 1
urethral cancer/Phase 1/Phase 2
urethral cancer/Phase 2
urethral cancer/Phase 2/Phase 3
urethral cancer/Phase 3
urethral carcinoma/Phase 3
urethral urothelial carcinoma/Phase 1
urinary tract cancer/Phase 2
urothelial bladder carcinoma/Phase 1
urothelial bladder carcinoma/Phase 2
urothelial bladder carcinoma/Phase 3
urothelial cancer/Phase 2
uterine cancer/Unspecified phase
uterine carcinosarcoma/Phase 2
uterine corpus cancer/Phase 1
uterine leiomyosarcoma/Phase 2

© 2000-2014 QIAGEN. All rights reserved.

				uterine leiomyosarcoma/Unspecified phase<br>uterine sarcoma/Phase 1<br>uterine sarcoma/Phase 2<br>uterine sarcoma/Phase 3<br>uterine sarcoma/Unspecified phase<br>vaginal cancer/Phase 1<br>vomiting/Unspecified phase<br>vulva cancer/Phase 1<br>vulva cancer/Phase 2<br>vulvar squamous cell carcinoma/Phase 2<br>Waldenstrom's macroglobulinemia/Phase 1<br>Waldenstrom's macroglobulinemia/Phase 1/Phase 2<br>Waldenstrom's macroglobulinemia/Phase 2
gemcitabine/irinotecan [gemcitabine]	RRM2	inhibitor		
gemcitabine/oxaliplatin [gemcitabine]	RRM2	inhibitor		biliary tract cancer/Phase 1/Phase 2<br>biliary tract cancer/Phase 2<br>malignant urothelial neoplasm/Phase 2<br>occult primary tumor/Phase 2<br>pancreatic cancer/Phase 2<br>prostate cancer/Phase 2
gemcitabine/oxaliplatin/paclitaxel [gemcitabine]	RRM2	inhibitor		
gemcitabine/oxaliplatin/rituximab [gemcitabine]	RRM2	inhibitor		
gemcitabine/paclitaxel [gemcitabine]	RRM2	inhibitor	Abraxane/gemcitabine, Gemzar/paclitaxel	breast cancer/Phase 2<br>colorectal carcinoma/Phase 1<br>melanoma/Phase 1<br>metastatic breast cancer/Approved<br>non-small cell lung cancer/Phase 1<br>ovarian cancer/Phase 1<br>pancreatic adenocarcinoma/Approved<br>pancreatic carcinoma/Phase 1<br>pancreatic ductal carcinoma/Phase 2<br>prostate cancer/Phase 1<br>renal-cell carcinoma/Phase 1<br>solid tumor/Phase 1
gemcitabine/pemetrexed [gemcitabine]	RRM2	inhibitor		
gemcitabine/vinorelbine [gemcitabine]	RRM2	inhibitor		
HM61713	EGFR	inhibitor		EGFR mutation positive non-small cell lung cancer/Phase 1<br>non-small cell lung cancer/Phase 2
hydroxyurea	RRM2	inhibitor	Biosupressin, Droxia, Hidrix, Hydrea, Hydreia, Hydura, Hydurea, Litaler, Litalir, Mylocel, Onco-Carbide, Oxyurea, Ureaphil	acquired immunodeficiency syndrome/Unspecified phase<br>acute myeloid leukemia/Phase 2<br>adrenoleukodystrophy/Phase 2<br>advanced solid tumor/Phase 1<br>astrocytoma/Phase 3<br>beta thalassemia/Phase 2<br>beta thalassemia/Unspecified phase<br>brain cancer/Phase 2<br>cancer/Phase 2<br>central nervous system tumor/Phase 1<br>central nervous system tumor/Phase 2<br>chronic kidney disease/Phase 1<br>chronic myeloid leukemia/Approved<br>cytogenetic abnormality positive loss of Y chromosome negative Philadelphia chromosome negative t(5;12)(q33;p13) negative CMML/Phase 3<br>desmoid tumor/Phase 2<br>esophageal cancer/Phase 1/Phase 2<br>esophageal cancer/Phase 2<br>essential thrombocythemia/Phase 3<br>extrahepatic bile duct cancer/Phase 2<br>fibromatosis/Phase 2<br>gastric cancer/Phase 2<br>glioblastoma cancer/Phase 1<br>glioblastoma cancer/Phase 2<br>glioblastoma/Phase 3<br>gliosarcoma cancer/Phase 1<br>gliosarcoma cancer/Phase 2<br>GM1 gangliosidosis/Phase 2<br>head and neck cancer/Phase 1/Phase 2<br>head and neck cancer/Phase 2<br>hemochromatosis/Phase 3<br>hemoglobin SC disease/Phase 2<br>hemoglobinopathy/Phase 2<br>hemoglobinopathy/Phase 3<br>high risk JAK2 V617F mutation positive polycythemia vera/Phase 3<br>hypopharyngeal squamous cell carcinoma/Phase 1<br>immunodeficiency/Phase 2<br>infection/Phase 1<br>infection/Phase 2<br>infection/Phase 3<br>infection/Phase 4<br>infection/Unspecified phase<br>JAK2 V617F mutation positive polycythemia vera/Phase 3

© 2000-2014 QIAGEN. All rights reserved.

Krabbe's disease/Phase 2
laryngeal cancer/Phase 2
laryngeal cancer/Phase 3
laryngeal squamous cell carcinoma/Phase 1
laryngeal squamous cell carcinoma/Phase 1/Phase 2
laryngeal verrucous carcinoma/Phase 1
laryngeal verrucous carcinoma/Phase 1/Phase 2
leukemia/Phase 2
leukemia/Phase 3
liver cancer/Phase 2
lung cancer/Phase 1
lymphocytic cancer/Phase 2
malaria/Phase 3
melanoma/Approved
meningioma/Phase 2
metachromatic leukodystrophy/Phase 2
mucolipidosis, type II/Phase 2
multiple sclerosis/Phase 2/Phase 3
myelodysplastic myeloproliferative neoplasm/Phase 2
myelodysplastic myeloproliferative neoplasm/Phase 3
myelodysplastic syndrome/Phase 2
myelodysplastic syndrome/Phase 3
myelofibrosis with myeloid metaplasia/Phase 3
myeloproliferative disorder/Unspecified phase
oropharyngeal squamous-cell carcinoma/Phase 1
pain crisis associated with sickle cell anemia/Approved
pancreatic cancer/Phase 2
PDGFBR rearrangement negative Philadelphia chromosome negative chronic myelomonocytic leukemia/Phase 3
Ph positive chronic myeloid leukemia in chronic phase/Phase 3
Philadelphia chromosome negative chronic myeloid neoplasm/Phase 3
Philadelphia chromosome negative t(5;12)(q33;p13) negative chronic myelomonocytic leukemia/Phase 3
Philadelphia-positive chronic myeloid leukemia/Phase 1/Phase 2
polycythemia vera/Approved
polycythemia vera/Phase 3
post-transplant lymphoproliferative disorder/Phase 2
pulmonary hypertension/Phase 0
pulmonary hypertension/Phase 1
pulmonary hypertension/Phase 1/Phase 2
recurrent laryngeal squamous cell carcinoma/Phase 1
salivary gland cancer/Phase 1
Sandhoff's disease/Phase 2
sickle cell anemia/Approved
sickle cell anemia/Phase 0
sickle cell anemia/Phase 1
sickle cell anemia/Phase 1/Phase 2
sickle cell anemia/Phase 2
sickle cell anemia/Phase 2/Phase 3
sickle cell anemia/Phase 3
sickle cell anemia/Phase 4
sickle cell anemia/Unspecified phase
small intestine cancer/Phase 2
spinal muscular atrophy/Phase 1/Phase 2
spinal muscular atrophy/Phase 2/Phase 3
squamous cell carcinoma of nasopharynx/Phase 1
stage III oropharyngeal squamous cell carcinoma/Phase 1
stage IV oropharyngeal squamous cell carcinoma/Phase 1
stage IVA oropharyngeal squamous cell carcinoma/Phase 1/Phase 2
stage IVB oropharyngeal squamous cell carcinoma/Phase 1/Phase 2
stroke/Phase 1/Phase 2
stroke/Phase 2
stroke/Phase 3
Tay-Sachs disease/Phase 2
thalassemia intermedia/Phase 2
thalassemia intermedia/Phase 2/Phase 3
thrombocytosis/Unspecified phase
tongue cancer/Phase 1
undifferentiated nasopharyngeal carcinoma/Phase 1
Wolman disease/Phase 2

© 2000-2014 QIAGEN. All rights reserved.
INGENUITY® SYSTEMS

Drug Name	Targets	Actions	Brand Names	Indications/Status
icotinib	EGFR	inhibitor		adult solid tumor/Phase 1<br>brain metastasis associated with EGFR mutation positive non-small cell lung cancer/Phase 2<br>brain metastasis associated with EGFR mutation positive non-small cell lung cancer/Phase 3<br>brain metastasis associated with metastatic non-small-cell lung cancer/Phase 2<br>brain metastasis associated with non-small cell lung cancer/Phase 2<br>brain metastasis/Phase 1/Phase 2<br>EGFR (L858R) positive lung adenocarcinoma/Phase 4<br>EGFR activating mutation positive adenocarcinoma of the lung/Phase 4<br>EGFR exon 19 deletion positive lung adenocarcinoma/Phase 4<br>EGFR exon 19 deletion positive non-small cell lung cancer/Phase 2<br>EGFR exon 19 deletion positive non-small cell lung cancer/Phase 4<br>EGFR exon 19 mutation positive lung adenocarcinoma/Phase 2<br>EGFR exon 19 mutation positive non-small cell lung cancer/Phase 2<br>EGFR exon 19 mutation positive non-small cell lung cancer/Phase 3<br>EGFR exon 21 mutation positive non-small cell lung cancer/Phase 2<br>EGFR exon 21 mutation positive non-small cell lung cancer/Phase 3<br>EGFR exon 21 mutation positive non-small cell lung cancer/Phase 4<br>EGFR exon 21 mutation-positive lung adenocarcinoma/Phase 2<br>EGFR L858R mutation positive non-small cell lung cancer/Phase 2<br>EGFR L858R mutation positive non-small cell lung cancer/Phase 4<br>EGFR mutation negative non-small cell lung cancer/Phase 2<br>EGFR mutation positive gastroesophageal junction carcinoma/Phase 2<br>EGFR mutation positive non-small cell lung cancer/Phase 1<br>EGFR mutation positive non-small cell lung cancer/Phase 2<br>EGFR mutation positive non-small cell lung cancer/Phase 3<br>EGFR mutation positive non-small cell lung cancer/Phase 4<br>EGFR sensitizing mutation positive lung adenocarcinoma/Phase 3<br>EGFR sensitizing mutation positive non-small cell lung cancer/Phase 3<br>esophageal cancer/Phase 2<br>esophageal carcinoma/Phase 2<br>esophageal squamous cell carcinoma/Phase 2<br>metastatic breast cancer/Phase 2<br>metastatic esophageal squamous cell carcinoma/Phase 2<br>metastatic non-small-cell lung cancer/Phase 2<br>nasopharyngeal carcinoma/Phase 1/Phase 2<br>nasopharyngeal carcinoma/Phase 2<br>non-small cell lung cancer with activating EGFR mutations/Phase 4<br>non-small cell lung cancer/Phase 1/Phase 2<br>non-small cell lung cancer/Phase 2<br>non-small cell lung cancer/Phase 3<br>non-small cell lung cancer/Phase 4<br>pancreatic cancer/Phase 1<br>squamous cell lung cancer/Phase 2
IMGN289	EGFR	inhibitor		EGFR expressing solid tumor/Phase 1
irinotecan/panitumumab [panitumumab]	EGFR	antibody		

© 2000-2014 QIAGEN. All rights reserved.

Drug Name	Targets	Actions	Brand Names	Indications/Status
itraconazole	CYP51A1	inhibitor	Hyphanox, Itrizole, Oriconazole, Sporal, Sporanos, Sporanox, Sporonox, Triasporn	advanced malignant tumor/Phase 1<br>advanced non-small-cell lung cancer/Phase 1<br>advanced solid tumor/Phase 1<br>ALK fusion positive non-small cell lung cancer/Phase 1<br>allergic bronchopulmonary aspergillosis/Phase 2/Phase 3<br>allergic bronchopulmonary aspergillosis/Phase 4<br>Alzheimer's disease/Phase 1<br>aspergillosis/Phase 2<br>basal cell carcinoma of skin/Phase 0<br>basal cell carcinoma of skin/Phase 2<br>blastomycosis/Phase 1/Phase 2<br>blastomycosis/Phase 3<br>brain tumor/Phase 4<br>bronchopulmonary aspergillosis/Approved<br>c-MET amplified non-small cell lung cancer/Phase 1<br>cancer/Phase 3<br>cancer/Unspecified phase<br>candidiasis/Approved<br>candidiasis/Phase 1<br>castration refractory prostate cancer/Phase 1/Phase 2<br>castration refractory prostate cancer/Phase 2<br>chronic hepatitis C/Phase 1/Phase 2<br>chronic kidney disease/Phase 1<br>chronic sinusitis/Phase 2/Phase 3<br>cryptococcal meningitis/Phase 2<br>cryptococcal meningitis/Unspecified phase<br>cystic fibrosis/Phase 1<br>cystic fibrosis/Phase 2<br>cystic fibrosis/Phase 4<br>dentin dysplasia type I/Phase 1<br>EGFR sensitizing mutation positive non-small cell lung cancer/Phase 1<br>esophageal candidiasis/Unspecified phase<br>febrile neutropenia/Phase 2<br>fungemia/Phase 3<br>graft-vs-host disease/Phase 4<br>hematological disorder/Phase 2<br>hepatic insufficiency/Phase 1<br>histoplasmosis/Approved<br>histoplasmosis/Phase 1<br>histoplasmosis/Phase 1/Phase 2<br>histoplasmosis/Phase 2<br>histoplasmosis/Phase 3<br>histoplasmosis/Unspecified phase<br>hormone refractory metastatic prostate cancer/Phase 2<br>hormone refractory prostate cancer/Phase 2<br>infection/Phase 1<br>infection/Phase 2<br>infection/Phase 3<br>infection/Unspecified phase<br>large-cell Ki-1 lymphoma/Phase 1<br>mammary tumor/Unspecified phase<br>metastasis/Unspecified phase<br>mycosis/Phase 2<br>mycosis/Phase 4<br>neuroblastoma/Phase 4<br>nocturia/Phase 1<br>non-small cell lung cancer/Phase 2<br>onychomycosis/Phase 2<br>oral candidiasis/Unspecified phase<br>prostate cancer/Phase 1/Phase 2<br>prostate cancer/Phase 2<br>prostatic tumor/Phase 2<br>relapsed multiple myeloma/Phase 1<br>retinoblastoma/Phase 4<br>ROS positive non-small cell lung cancer/Phase 1<br>sinusitis/Phase 1<br>skin cancer/Phase 2<br>solid tumor/Phase 1<br>thrombosis/Phase 1<br>tumor/Phase 1<br>Wilm's tumorigenesis/Phase 4

© 2000-2014 QIAGEN. All rights reserved.

Drug Name	Targets	Actions	Brand Names	Indications/Status
ketoconazole	CYP51A1	inhibitor	Extina, Fungarest, Fungoral, Ketoderm, Ketoisdin, Ketozole, Nizoral, Nizoral a-D, Nizoral a-D Shampoo, Nizoral Cream, Nizoral Shampoo, Orifungal, Orifungal M, Panfungol, Sebazole	acute respiratory distress syndrome/Phase 3<br>adult solid tumor/Phase 1<br>adult solid tumor/Phase 1/Phase 2<br>advanced cancer/Phase 1<br>age-related macular degeneration/Phase 1<br>Alzheimer's disease/Phase 1<br>asthma/Phase 1<br>burn/Phase 2/Phase 3<br>cancer/Phase 1<br>cancer/Phase 2<br>candidiasis/Approved<br>candidiasis/Phase 3<br>carcinoma/Phase 1<br>chemotherapy sickness/Phase 1<br>chronic central serous chorioretinopathy/Phase 2<br>chronic lymphocytic leukemia/Phase 1<br>chronic obstructive pulmonary disease/Phase 1<br>chronic obstructive pulmonary disease/Phase 4<br>dandruff/Approved<br>esophageal candidiasis/Unspecified phase<br>essential hypertension/Phase 1<br>FOXL2 C134W mutation positive ovarian granulosa cell carcinoma/Phase 2<br>gastroparesis/Phase 1<br>hematologic cancer/Phase 1<br>hip fracture/Phase 2<br>histoplasmosis/Approved<br>Hodgkin's disease/Phase 1<br>Huntington's disease/Phase 1<br>hypogonadism/Unspecified phase<br>hypogonadotropic hypogonadism/Phase 2<br>impotence/Phase 1<br>infection/Unspecified phase<br>insulin resistance/Unspecified phase<br>Kallmann's syndrome/Unspecified phase<br>large-cell Ki-1 lymphoma/Phase 1<br>lymphocytic cancer/Phase 1<br>macular degeneration/Phase 1<br>melanoma/Phase 1<br>metastatic bone cancer/Phase 2<br>metastatic breast cancer/Phase 2<br>multiple sclerosis/Phase 1<br>neuroblastoma/Phase 1<br>neuroblastoma/Unspecified phase<br>non-Hodgkin's disease/Phase 1<br>non-insulin-dependent diabetes mellitus/Phase 1<br>osteoarthritis/Phase 2<br>osteoporosis/Phase 1<br>ovarian cancer/Phase 1/Phase 2<br>primary peritoneal cancer/Phase 1/Phase 2<br>prostate cancer/Phase 1/Phase 2<br>prostate cancer/Phase 2<br>prostate cancer/Phase 3<br>prostate cancer/Unspecified phase<br>prostatic adenocarcinoma/Phase 2<br>prostatic tumor/Phase 2<br>psoriasis/Phase 2<br>pulmonary disease/Phase 3<br>recurrent prostate cancer/Phase 2<br>schizophrenia/Phase 1<br>seborrheic dermatitis/Approved<br>seborrheic dermatitis/Unspecified phase<br>solid tumor/Phase 1<br>solid tumor/Phase 2<br>thromboembolism/Phase 2<br>tinea pedis/Phase 3<br>tumor/Phase 1
L-asparaginase/gemcitabine/oxaliplatin [gemcitabine]	RRM2	inhibitor		
L19-IL2 monoclonal antibody-cytokine fusion protein	FN1	binder		advanced solid tumor/Phase 1/Phase 2
lapatinib	EGFR	antagonist	Tycerb, Tykerb, Tyverb	acoustic neuroma/Phase 0<br>acoustic neuroma/Phase 2<br>adenocarcinoma/Phase 2<br>adenoid cystic carcinoma of salivary gland/Phase 2<br>adult solid tumor/Phase 1<br>advanced breast cancer/Phase 2<br>advanced cancer/Phase 1<br>advanced colorectal cancer/Phase 1<br>advanced non-small-cell lung cancer/Phase 1<br>advanced non-small-cell lung cancer/Phase 2<br>advanced solid tumor/Phase 1<br>advanced solid tumor/Unspecified phase<br>bladder cancer/Phase 1<br>bladder cancer/Phase 2

© 2000-2014 QIAGEN. All rights reserved.
INGENUITY® SYSTEMS

bladder carcinoma/Phase 0
BRAF V600E mutation positive thyroid cancer/Phase 1
BRAF V600E mutation positive thyroid carcinoma/Phase 1
BRAF V600K mutation positive thyroid cancer/Phase 1
BRAF V600K mutation positive thyroid carcinoma/Phase 1
brain cancer/Phase 2
brain metastasis associated with brain metastasis/Phase 2
brain metastasis associated with EGFR expressing breast cancer/Phase 2
brain metastasis associated with EGFR expressing lung cancer/Phase 2
brain metastasis associated with HER2 positive metastatic breast cancer/Phase 2
brain metastasis/Phase 1
brain metastasis/Phase 3
brain tumor/Phase 2
breast cancer/Phase 0
breast cancer/Phase 1
breast cancer/Phase 1/Phase 2
breast cancer/Phase 2
breast cancer/Phase 3
breast cancer/Phase 4
breast carcinoma/Phase 1
breast carcinoma/Phase 3
cancer/Phase 1
cancer/Phase 2
cancer/Unspecified phase
cardiovascular toxicity/Phase 2
CDKN2A negative oropharyngeal squamous cell cancer/Phase 2
central nervous system metastasis/Phase 2
central nervous system tumor/Phase 1/Phase 2
childhood anaplastic astrocytoma/Phase 1/Phase 2
childhood brain stem glioma/Phase 1/Phase 2
childhood giant cell glioblastoma/Phase 1/Phase 2
childhood gliosarcoma/Phase 1/Phase 2
childhood medulloblastoma/Phase 1/Phase 2
colorectal cancer/Phase 1
colorectal cancer/Phase 2
colorectal tumor/Phase 1
ductal carcinoma in situ/Unspecified phase
EGFR expressing esophagogastric junction cancer/Phase 2
EGFR expressing stomach cancer/Phase 2
EGFR positive bladder cancer/Phase 2/Phase 3
endometrial carcinoma/Phase 2
epithelial cancer/Phase 0
epithelial ovarian cancer/Phase 1/Phase 2
epithelial ovarian cancer/Phase 2
esophageal adenocarcinoma/Phase 1/Phase 2
estrogen receptor positive HER2 negative breast cancer/Phase 3
estrogen receptor positive HER2 negative progesterone receptor positive breast cancer/Phase 3
estrogen receptor positive HER2 positive breast cancer/Phase 2
estrogen receptor positive HER2 positive invasive breast cancer/Phase 3
extrahepatic bile duct cancer/Phase 2
gallbladder cancer/Phase 2
Gardner syndrome/Phase 2
gastric adenocarcinoma/Phase 2/Phase 3
gastric cancer/Phase 2
gastroesophageal cancer/Phase 2
gastrointestinal tumor/Phase 2
gastrointestinal tumor/Phase 3
glioblastoma/Phase 2
glioma cancer/Phase 2
gliosarcoma cancer/Phase 2
head and neck cancer/Phase 1
head and neck cancer/Phase 1/Phase 2
head and neck cancer/Phase 2
head and neck squamous cell carcinoma/Phase 2
head and neck tumor/Phase 2
head and neck tumor/Phase 3
HER2 expressing gastric cancer/Phase 2
HER2 expressing gastroesophageal junction cancer/Phase 2
HER2 negative progesterone

© 2000-2014 QIAGEN. All rights reserved.
INGENUITY® SYSTEMS

receptor positive breast cancer/Phase 3
HER2 non-overexpressing hormone receptor positive breast cancer/Phase 3
HER2 positive bladder cancer/Phase 2/Phase 3
HER2 positive breast adenocarcinoma/Phase 1
HER2 positive breast adenocarcinoma/Phase 1/Phase 2
HER2 positive breast adenocarcinoma/Phase 2
HER2 positive cancer/Phase 2
HER2 positive early breast cancer/Phase 2
HER2 positive endometrial carcinoma/Phase 1
HER2 positive esophageal adenocarcinoma/Phase 2
HER2 positive esophageal cancer/Phase 1
HER2 positive gastric cancer/Phase 1
HER2 positive gastric cardia adenocarcinoma/Phase 2
HER2 positive gastroesophageal junction adenocarcinoma/Phase 1
HER2 positive gastroesophageal junction adenocarcinoma/Phase 2
HER2 positive hormone receptor positive malignant neoplasm of breast/Phase 3
HER2 positive invasive breast cancer/Phase 1/Phase 2
HER2 positive invasive breast cancer/Phase 2
HER2 positive invasive breast cancer/Phase 3
HER2 positive invasive breast carcinoma/Phase 1
HER2 positive invasive breast carcinoma/Phase 2
HER2 positive metastatic breast cancer/Phase 1
HER2 positive metastatic breast cancer/Phase 1/Phase 2
HER2 positive metastatic breast cancer/Phase 2
HER2 positive metastatic breast cancer/Phase 3
HER2 positive metastatic breast cancer/Phase 4
HER2 positive progesterone receptor positive malignant breast neoplasm/Phase 3
HER2 positive solid tumor/Phase 1
HER2-positive breast cancer/Phase 0
HER2-positive breast cancer/Phase 1
HER2-positive breast cancer/Phase 1/Phase 2
HER2-positive breast cancer/Phase 2
HER2-positive breast cancer/Phase 3
HER2-positive breast cancer/Unspecified phase
HER2-positive carcinoma of breast/Phase 2
HER2-positive carcinoma of breast/Phase 3
HER2-positive male breast cancer/Phase 1
hormone receptor positive breast cancer/Phase 2
hormone receptor positive HER2 negative invasive breast cancer/Phase 2
hypopharyngeal squamous cell carcinoma/Phase 2
intestinal gastric adenocarcinoma/Phase 2
invasive breast cancer/Phase 3
invasive estrogen receptor negative HER2 positive breast cancer/Phase 3
invasive HER2 positive ductal breast carcinoma/Phase 2
KRAS exon 2 mutation positive PIK3CA exon 9 mutation negative PIK3CA exon 20 mutation negative colorectal cancer/Phase 1/Phase 2
KRAS exon 2 mutation positive PIK3CA exon 9 mutation negative PIK3CA exon 20 mutation negative non-small cell lung cancer/Phase 1/Phase 2
KRAS exon 2 mutation positive PIK3CA exon 9 mutation negative PIK3CA exon 20 mutation negative pancreatic cancer/Phase 1/Phase 2
KRAS exon 3 mutation positive PIK3CA exon 9 mutation negative PIK3CA exon 20 mutation negative colorectal cancer/Phase 1/Phase 2

© 2000-2014 QIAGEN. All rights reserved.

KRAS exon 3 mutation positive
PIK3CA exon 9 mutation negative
PIK3CA exon 20 mutation negative
non-small cell lung cancer/Phase 1/Phase 2
KRAS exon 3 mutation positive
PIK3CA exon 9 mutation negative
PIK3CA exon 20 mutation negative
pancreatic cancer/Phase 1/Phase 2
KRAS exon 4 mutation positive
PIK3CA exon 9 mutation negative
PIK3CA exon 20 mutation negative
colorectal cancer/Phase 1/Phase 2
KRAS exon 4 mutation positive
PIK3CA exon 9 mutation negative
PIK3CA exon 20 mutation negative
non-small cell lung cancer/Phase 1/Phase 2
KRAS exon 4 mutation positive
PIK3CA exon 9 mutation negative
PIK3CA exon 20 mutation negative
pancreatic cancer/Phase 1/Phase 2
laryngeal squamous cell carcinoma/Phase 2
laryngeal verrucous carcinoma/Phase 2
liver metastasis associated with HER2 positive gastric cancer/Phase 2
locally advanced EGFR positive bladder cancer/Phase 2/Phase 3
locally advanced HER2 positive bladder cancer/Phase 2/Phase 3
locally advanced HER2 positive breast cancer/Phase 1/Phase 2
locally advanced HER2 positive breast cancer/Phase 2
lung cancer/Phase 1
lymphocytic cancer/Phase 1
male breast cancer/Phase 1/Phase 2
male breast cancer/Phase 2
male breast carcinoma/Phase 3
mammary tumor/Phase 1
mammary tumor/Phase 2
mammary tumor/Phase 3
mammary tumor/Phase 4
melanoma/Phase 2
metastasis/Phase 1
metastasis/Phase 1/Phase 2
metastasis/Phase 2
metastatic breast cancer/Phase 1
metastatic breast cancer/Phase 1/Phase 2
metastatic breast cancer/Phase 2
metastatic breast cancer/Phase 3
metastatic cervical cancer/Phase 2
metastatic colorectal cancer/Phase 1
metastatic colorectal cancer/Phase 2
metastatic estrogen receptor positive HER2 positive malignant neoplasm of breast/Phase 3
metastatic HER2 positive invasive breast cancer/Phase 1
metastatic pancreatic cancer/Phase 2
metastatic solid tumor/Phase 1
metastatic triple negative breast cancer/Unspecified phase
mixed gastric adenocarcinoma/Phase 2
neurofibromatosis 2/Phase 0
neurofibromatosis 2/Phase 2
non-small cell lung cancer/Phase 1
oropharyngeal squamous-cell carcinoma/Phase 2
ovarian cancer/Phase 1
ovarian cancer/Phase 1/Phase 2
ovarian cancer/Phase 2
pancreatic cancer/Phase 2
pediatric cancer/Phase 2
pediatric glioblastoma/Phase 1/Phase 2
peritoneal cavity cancer/Phase 2
pituitary gland adenoma/Phase 2
primary peritoneal cancer/Phase 2
prolactinoma/Phase 2
prostate cancer/Phase 1/Phase 2
prostate cancer/Phase 2
recurrent breast cancer/Phase 1/Phase 2
recurrent breast cancer/Phase 2
recurrent laryngeal squamous cell carcinoma/Phase 2
recurrent ovarian cancer/Phase 2
recurrent peritoneal cancer/Phase 2
recurrent prostate cancer/Phase 2
renal-cell carcinoma/Phase 1
salivary gland adenocarcinoma/Phase 2
salivary gland cancer/Phase 2
salivary gland carcinoma/Phase 2
salivary gland mucoepidermoid carcinoma/Phase 2

© 2000-2014 QIAGEN. All rights reserved.

				small cell lung cancer/Phase 2<br>solid tumor/Phase 1<br>solid tumor/Phase 4<br>spinal cord neoplasm/Phase 2<br>squamous cell cancer of the hypopharynx/Phase 2<br>squamous cell carcinoma of nasopharynx/Phase 2<br>squamous cell carcinoma/Phase 2<br>stage II breast cancer/Phase 2<br>stage IIA breast cancer/Phase 3<br>stage IIB breast cancer/Phase 3<br>stage III gastric cancer/Phase 2<br>stage IV gastric cancer/Phase 2<br>stage IV oropharyngeal squamous cell carcinoma/Phase 2<br>stage IVA oropharyngeal squamous cell carcinoma/Phase 2<br>stage IVB oropharyngeal squamous cell carcinoma/Phase 2<br>stage IVC oropharyngeal squamous cell carcinoma/Phase 2<br>thymus cancer/Phase 2<br>tongue cancer/Phase 2<br>transitional cell bladder cancer/Phase 2<br>tumor/Phase 1<br>urothelial cancer/Phase 2
lapatinib/letrozole [lapatinib]	EGFR	antagonist		HER2 positive hormone receptor positive metastatic breast cancer/Approved
lapatinib/paclitaxel [lapatinib]	EGFR	antagonist		
lapatinib/pazopanib [lapatinib]	EGFR	antagonist		
lapatinib/trastuzumab [lapatinib]	EGFR	antagonist	Tyverb/trastuzumab	
lovastatin/niacin [nicotinic acid]	QPRT	binder	Advicor	hypercholesterolemia/Approved<br>hyperlipidemia/Approved
luliconazole	CYP51A1	inhibitor	Luzu	dermatophytosis/Phase 4<br>onychomycosis/Phase 1/Phase 2<br>tinea corporis associated with infection/Approved<br>tinea cruris associated with infection/Approved<br>tinea pedis associated with infection/Approved<br>tinea pedis/Phase 2
LY3164530	EGFR	antibody		advanced cancer/Phase 1<br>metastasis/Phase 1
MEHD7945A	EGFR	binder		head and neck squamous cell carcinoma/Phase 2<br>KRAS mutation negative adenocarcinoma of the colon/Phase 2<br>KRAS mutation negative adenocarcinoma of the rectum/Phase 2<br>KRAS mutation positive colorectal cancer/Phase 1<br>KRAS mutation positive non small cell lung cancer/Phase 1<br>KRAS mutation positive solid tumor/Phase 1<br>KRAS wild-type metastatic colorectal cancer/Phase 2<br>metastatic KRAS mutation negative colorectal adenocarcinoma/Phase 2<br>stage IV head and neck squamous cell carcinoma/Phase 2
miconazole	CYP51A1	inhibitor	Daktarin, Femizol-M, M-zole 3 Combination Pack, M-zole 7 Dual Pack, Micatin, Miconazole 3, Miconazole 3 Combination Pack, Miconazole 7, Miconazole 7 Combination Pack, Miconazole Nitrate, Miconazole Nitrate Combination Pack, Miconazole-7, Micozole, Minostate, Monazole 7, Monistat, Monistat 1 Combination Pack, Monistat 3, Monistat 3 Combination Pack, Monistat 3 Dual-Pak, Monistat 3 Vaginal Ovules, Monistat 5, Monistat 5 Tampon, Monistat 7, Monistat 7 Combination Pack, Monistat 7 Dual-Pak, Monistat 7 Vaginal Suppositories, Monistat Dual- PAK, Monistat IV, Monistat-1 Dual Pack, Monistat-3 Combination Pack, Monistat-Derm, Novo-Miconazole Vaginal Ovules, Oravig, Vusion, Zimycan	candidiasis/Approved<br>infection/Phase 1<br>melasma/Phase 4<br>tinea corporis/Approved<br>tinea cruris/Approved<br>tinea pedis/Approved

© 2000-2014 QIAGEN. All rights reserved.

Drug Name	Targets	Actions	Brand Names	Indications/Status
mipomersen	APOB	antisense	Kynamro	atherosclerosis/Phase 3<br>autosomal dominant hypercholesterolemia/Phase 2<br>autosomal dominant hypercholesterolemia/Phase 3<br>congenital malformation/Phase 2<br>congenital malformation/Phase 3<br>coronary artery disease/Phase 3<br>coronary disease/Phase 3<br>disorder of lipid metabolism/Phase 2<br>disorder of lipid metabolism/Phase 3<br>dyslipidemia/Phase 2<br>dyslipidemia/Phase 3<br>familial hypercholesterolemia/Approved<br>familial hypercholesterolemia/Phase 2<br>heterozygous familial hypercholesterolemia/Phase 3<br>hypercholesterolemia/Phase 2<br>hypercholesterolemia/Phase 3<br>hyperlipidemia/Phase 2<br>hyperlipidemia/Phase 3<br>hyperlipoproteinemia/Phase 2<br>hyperlipoproteinemia/Phase 3<br>hypobetalipoproteinemia/Phase 2<br>hypolipoproteinemia/Phase 2<br>inborn error of lipid metabolism/Phase 2<br>inborn error of lipid metabolism/Phase 3<br>metabolic disorder/Phase 2<br>metabolic disorder/Phase 3
necitumumab	EGFR	binder		advanced solid tumor/Phase 1<br>advanced solid tumor/Phase 2<br>ALK translocation positive non-small cell lung cancer/Phase 1<br>malignant solid tumor/Phase 2<br>metastatic colorectal cancer/Phase 2<br>metastatic non-small-cell lung cancer/Phase 1<br>metastatic solid tumor/Phase 2<br>non-small cell lung cancer with activating EGFR mutations/Phase 1<br>non-small cell lung cancer/Phase 2<br>non-small cell lung cancer/Phase 3<br>solid tumor/Phase 1<br>squamous cell lung cancer/Phase 1/Phase 2<br>squamous cell lung cancer/Phase 2

© 2000-2014 QIAGEN. All rights reserved.

Drug Name	Targets	Actions	Brand Names	Indications/Status
neratinib	EGFR	inhibitor		advanced breast cancer/Phase 1/Phase 2<br>advanced breast cancer/Phase 2<br>advanced solid tumor/Phase 1<br>advanced solid tumor/Phase 1/Phase 2<br>BRAF mutation negative KRAS mutation negative NRAS mutation negative PIK3CA mutation negative colorectal cancer/Phase 1/Phase 2<br>brain metastasis associated with HER2 positive invasive breast cancer/Phase 2<br>breast cancer/Phase 1<br>breast cancer/Phase 1/Phase 2<br>breast cancer/Phase 2<br>breast cancer/Phase 3<br>carcinoma/Phase 1<br>EGFR activating mutation positive solid tumor/Phase 2<br>EGFR expressing solid tumor/Phase 2<br>hepatic insufficiency/Phase 1<br>HER2 activating mutation positive non-small cell lung cancer/Phase 2<br>HER2 activating mutation positive solid tumor/Phase 2<br>HER2 mutation positive HER2 non-overexpressing breast cancer/Phase 2<br>HER2 negative hormone receptor negative breast cancer/Phase 1/Phase 2<br>HER2 positive hormone receptor negative breast cancer/Phase 2<br>HER2 positive invasive breast adenocarcinoma/Phase 1/Phase 2<br>HER2 positive metastatic breast cancer/Phase 1/Phase 2<br>HER2-negative breast cancer/Phase 2<br>HER2-positive breast cancer/Phase 3<br>HER3 activating mutation positive solid tumor/Phase 2<br>hormone receptor positive HER2 positive breast cancer/Phase 2<br>lung tumor/Phase 2<br>mammary tumor/Phase 1<br>mammary tumor/Phase 1/Phase 2<br>mammary tumor/Phase 2<br>metastatic HER2 positive invasive breast cancer/Phase 1/Phase 2<br>non-small cell lung cancer/Phase 2<br>tumor/Phase 1<br>tumor/Phase 2
nicotinic acid	QPRT	binder	Acido Nicotino, Akotin, Niaspan, Nico 400, Nicolar, Niconacid, Nicotinic Acid, Nikacid, Nikotime, Novoniacin, Slo-Niacin, Span Niacin, Vitaplex, Wampocap	arterial occlusive disease/Phase 3<br>atherogenic dyslipidemia/Phase 2<br>atherosclerosis/Phase 3<br>atherosclerosis/Phase 4<br>atherosclerosis/Unspecified phase<br>cardiovascular disorder/Phase 2<br>cardiovascular disorder/Phase 3<br>cardiovascular disorder/Unspecified phase<br>carotid artery disease/Phase 3<br>carotid artery disease/Phase 4<br>carotid artery disease/Unspecified phase<br>central retinal vein occlusion/Phase 2/Phase 3<br>cerebrovascular dysfunction/Phase 3<br>chronic kidney disease/Phase 4<br>chronic renal failure/Phase 3<br>coronary artery disease/Phase 2<br>coronary artery disease/Phase 3<br>coronary artery disease/Phase 4<br>coronary artery disease/Unspecified phase<br>coronary disease/Phase 2<br>coronary disease/Phase 3<br>coronary disease/Phase 4<br>coronary disease/Unspecified phase<br>diabetes mellitus/Phase 3<br>diabetes mellitus/Phase 4<br>diabetes mellitus/Unspecified phase<br>dyslipidemia/Phase 1<br>dyslipidemia/Phase 2<br>dyslipidemia/Phase 3<br>dyslipidemia/Phase 4<br>dyslipidemia/Unspecified phase<br>endothelial dysfunction/Unspecified phase<br>flushing response/Phase 2<br>flushing response/Phase 3<br>growth hormone deficiency/Phase 1/Phase 2<br>heart disease/Phase 2<br>heart disease/Phase 3<br>heart disease/Unspecified phase<br>high density lipoprotein deficency/Phase 4

© 2000-2014 QIAGEN. All rights reserved.

			high density lipoprotein deficency/Unspecified phase<br>hypercholesterolemia/Approved<br>hypercholesterolemia/Phase 1<br>hypercholesterolemia/Phase 2<br>hypercholesterolemia/Phase 3<br>hypercholesterolemia/Phase 4<br>hypercholesterolemia/Unspecified phase<br>hyperlipidemia/Approved<br>hyperlipidemia/Phase 1<br>hyperlipidemia/Phase 3<br>hyperlipidemia/Phase 4<br>hyperlipidemia/Unspecified phase<br>hyperlipoproteinemia/Phase 4<br>hypertriglyceridemia/Approved<br>hypertriglyceridemia/Phase 4<br>hypertriglyceridemia/Unspecified phase<br>hypolipoproteinemia/Phase 4<br>infection/Phase 2<br>infection/Phase 4<br>infection/Unspecified phase<br>inflammation/Unspecified phase<br>insulin resistance/Phase 4<br>insulin resistance/Unspecified phase<br>intermittent claudication/Phase 3<br>ischemic stroke/Phase 2<br>metabolic syndrome X/Phase 3<br>metabolic syndrome X/Phase 4<br>mixed hyperlipidemia/Phase 1<br>mixed hyperlipidemia/Phase 3<br>mixed hyperlipidemia/Phase 4<br>myocardial infarction/Phase 3<br>myocardial infarction/Unspecified phase<br>myocardial ischemia/Phase 2<br>myocardial ischemia/Phase 3<br>myocardial ischemia/Unspecified phase<br>non-insulin-dependent diabetes mellitus/Phase 1<br>non-insulin-dependent diabetes mellitus/Phase 4<br>nonalcoholic fatty liver disease/Unspecified phase<br>obesity/Phase 1/Phase 2<br>obesity/Phase 2<br>pain/Phase 3<br>peripheral arterial disease/Phase 3<br>peripheral arterial disease/Phase 4<br>peripheral arterial disease/Unspecified phase<br>peripheral vascular disease/Phase 3<br>primary hypercholesterolemia/Phase 1<br>primary hypercholesterolemia/Phase 3<br>psychosis/Phase 3<br>retinal vein occlusion/Phase 2/Phase 3<br>short stature/Phase 1/Phase 2<br>sickle cell anemia/Phase 2<br>stroke/Phase 3<br>stroke/Phase 4<br>vascular disease/Phase 3<br>venous retinal branch occlusion/Phase 2/Phase 3
nicotinic acid/pioglitazone [nicotinic acid]	QPRT	binder	

© 2000-2014 QIAGEN. All rights reserved.

Drug Name	Targets	Actions	Brand Names	Indications/Status
nimotuzumab	EGFR	inhibitor	Theraloc	adenocarcinoma/Phase 2<br>adenocarcinoma/Phase 2/Phase 3<br>carcinoma/Phase 2<br>cervical cancer/Phase 1/Phase 2<br>cervical cancer/Phase 2<br>cervical squamous cell carcinoma/Phase 2<br>colorectal cancer/Phase 2<br>EGFR exon 19 deletion positive non-small cell lung cancer/Phase 1/Phase 2<br>EGFR expressing adenocarcinoma of the gastroesophageal junction/Phase 3<br>EGFR expressing esophagogastric junction cancer/Phase 3<br>EGFR expressing gastric adenocarcinoma/Phase 3<br>EGFR expressing non-small cell lung cancer/Phase 2<br>EGFR expressing stomach cancer/Phase 3<br>EGFR L858R mutation positive non-small cell lung cancer/Phase 1/Phase 2<br>esophageal cancer/Phase 2/Phase 3<br>esophageal squamous cell cancer/Phase 1/Phase 2<br>esophageal squamous cell cancer/Phase 2<br>esophageal squamous cell carcinoma/Phase 2<br>esophageal squamous cell carcinoma/Phase 2/Phase 3<br>gastric cancer/Phase 2<br>head and neck cancer/Phase 2<br>head and neck squamous cell carcinoma/Phase 2<br>HER2 negative hormone receptor negative breast cancer/Phase 2<br>hypopharyngeal squamous cell carcinoma/Phase 2<br>hypopharyngeal squamous cell carcinoma/Phase 3<br>hypopharynx carcinoma/Phase 3<br>KRAS mutation negative pancreatic adenocarcinoma/Phase 3<br>KRAS mutation negative pancreatic cancer/Phase 3<br>KRAS wild-type metastatic colorectal cancer/Phase 2<br>KRAS wild-type positive colorectal cancer/Phase 2<br>laryngeal carcinoma/Phase 3<br>laryngeal squamous cell carcinoma/Phase 3<br>locally advanced rectal cancer/Phase 2<br>loco-regionally advanced squamous cell carcinoma of the head and neck/Phase 2<br>metastatic esophageal squamous cell carcinoma/Phase 2<br>metastatic non-small-cell lung cancer/Phase 1<br>metastatic non-small-cell lung cancer/Phase 2<br>metastatic triple negative breast cancer/Phase 2<br>nasopharyngeal cancer/Phase 3<br>nasopharyngeal carcinoma/Phase 4<br>non-small cell lung cancer/Phase 1<br>non-small cell lung cancer/Phase 1/Phase 2<br>non-small cell lung cancer/Phase 2<br>primary esophageal squamous cell carcinoma/Phase 3<br>solid tumor/Phase 1<br>squamous cell carcinoma of the oral cavity/Phase 3<br>stage III oropharyngeal squamous cell carcinoma/Phase 2<br>stage III oropharyngeal squamous cell carcinoma/Phase 3<br>stage IV nasopharyngeal carcinoma/Phase 2<br>stage IV oropharyngeal squamous cell carcinoma/Phase 3<br>stage IVB oropharyngeal squamous cell carcinoma/Phase 2<br>stomach tumor/Phase 2

© 2000-2014 QIAGEN. All rights reserved.

Drug Name	Targets	Actions	Brand Names	Indications/Status
ocriplasmin	FN1	enzyme	Jetrea	acute stroke/Phase 2<br>arterial occlusive disease/Phase 2<br>deep vein thrombosis/Phase 2<br>diabetic macular edema/Phase 2<br>exudative age-related macular degeneration/Phase 2<br>macular degeneration/Phase 2<br>ophthalmic disorder/Phase 2<br>stroke/Phase 2<br>uveitis/Phase 1/Phase 2<br>vitreomacular adhesion/Approved<br>vitreomacular adhesion/Phase 3<br>vitreomacular adhesion/Phase 4
oxiconazole	CYP51A1	inhibitor	Oxistat	tinea corporis/Approved<br>tinea cruris/Approved<br>tinea pedis/Approved<br>tinea pedis/Phase 1
panitumumab	EGFR	antibody	Vectibix	adenocarcinoma of the thoracic esophagus/Phase 2<br>adenocarcinoma/Phase 1<br>adenosquamous lung carcinoma/Phase 2<br>advanced rectal adenocarcinoma/Phase 3<br>advanced renal cell carcinoma/Phase 2<br>advanced solid tumor/Phase 1<br>biliary tract cancer/Phase 2<br>bladder cancer/Phase 2<br>BRAF mutation negative KRAS mutation negative colorectal cancer/Phase 2<br>BRAF mutation negative KRAS mutation negative NRAS mutation negative colorectal cancer/Phase 2<br>BRAF mutation negative KRAS mutation negative NRAS mutation negative PIK3CA mutation negative colon adenocarcinoma/Phase 2<br>BRAF mutation negative KRAS mutation negative NRAS mutation negative PIK3CA mutation negative colorectal cancer/Phase 2<br>BRAF mutation negative KRAS mutation negative NRAS mutation negative PIK3CA mutation negative rectal adenocarcinoma/Phase 2<br>BRAF V600E mutation positive colorectal cancer/Phase 2<br>brain glioma/Phase 2<br>breast cancer/Phase 2<br>c-MET overexpressing KRAS mutation negative colorectal cancer/Phase 1<br>cancer/Phase 1/Phase 2<br>cancer/Phase 2<br>carcinoma/Phase 2<br>cholangiocarcinoma/Phase 2<br>colon adenocarcinoma/Phase 3<br>colon cancer/Phase 1<br>colon cancer/Phase 1/Phase 2<br>colon cancer/Phase 2<br>colorectal cancer/Phase 1<br>colorectal cancer/Phase 1/Phase 2<br>colorectal cancer/Phase 2<br>colorectal cancer/Phase 3<br>colorectal carcinoma/Phase 2<br>colorectal tumor/Phase 1/Phase 2<br>colorectal tumor/Phase 2<br>epithelial ovarian cancer/Phase 2<br>esophageal adenocarcinoma/Phase 1/Phase 2<br>esophageal adenocarcinoma/Phase 2<br>esophageal cancer/Phase 1<br>esophageal cancer/Phase 1/Phase 2<br>esophageal cancer/Phase 2<br>esophageal squamous cell carcinoma/Phase 3<br>exanthem/Phase 2<br>gallbladder cancer/Phase 2<br>gastric adenocarcinoma/Phase 1/Phase 2<br>gastroesophageal adenocarcinoma/Phase 2<br>gastrointestinal tract cancer/Phase 1/Phase 2<br>head and neck cancer/Phase 2<br>head and neck cancer/Phase 3<br>head and neck squamous cell carcinoma/Phase 1<br>head and neck squamous cell carcinoma/Phase 2<br>head and neck tumor/Phase 2<br>HER2 negative esophageal adenocarcinoma/Phase 2<br>HER2 negative gastric adenocarcinoma/Phase 2<br>HER2 negative gastric cardia adenocarcinoma/Phase 2<br>HER2 negative gastroesophageal junction adenocarcinoma/Phase 2<br>HER2 negative inflammatory breast

© 2000-2014 QIAGEN. All rights reserved.

carcinoma/Phase 2
KRAS codon 12 mutation negative codon 13 mutation negative colorectal cancer/Approved
KRAS codon 12 mutation negative colorectal adenocarcinoma/Phase 2
KRAS codon 12 mutation positive colorectal cancer/Phase 2
KRAS codon 13 mutation positive colorectal cancer/Phase 2
KRAS codon 61 mutation positive colorectal cancer/Phase 2
KRAS mutation negative adenocarcinoma of ampulla of vater/Phase 2
KRAS mutation negative adenocarcinoma of the colon/Phase 1
KRAS mutation negative adenocarcinoma of the rectum/Phase 1
KRAS mutation negative adenocarcinoma of the small bowel/Phase 2
KRAS mutation negative and NRAS mutation negative colorectal cancer/Phase 2
KRAS mutation negative and NRAS mutation negative colorectal cancer/Phase 3
KRAS mutation negative and NRAS mutation negative colorectal cancer/Phase 4
KRAS mutation negative and NRAS mutation negative rectal cancer/Phase 2
KRAS mutation negative cervical cancer/Phase 2
KRAS mutation negative cholangiocarcinoma/Phase 2
KRAS mutation negative colorectal adenocarcinoma/Phase 2
KRAS mutation negative epithelial ovarian cancer/Phase 2
KRAS mutation negative fallopian tube cancer/Phase 2
KRAS mutation negative gall bladder carcinoma/Phase 2
KRAS mutation negative MET amplification positive colon adenocarcinoma/Phase 1
KRAS mutation negative MET amplification positive rectal adenocarcinoma/Phase 1
KRAS mutation negative NRAS mutation negative colon adenocarcinoma/Phase 4
KRAS mutation negative NRAS mutation negative colon cancer/Phase 2
KRAS mutation negative NRAS mutation negative colorectal adenocarcinoma/Phase 2
KRAS mutation negative NRAS mutation negative mucinous adenocarcinoma of the colon/Phase 2
KRAS mutation negative NRAS mutation negative mucinous adenocarcinoma of the rectum/Phase 2
KRAS mutation negative NRAS mutation negative rectal adenocarcinoma/Phase 4
KRAS mutation negative NRAS mutation negative signet ring adenocarcinoma of the colon/Phase 2
KRAS mutation negative NRAS mutation negative signet ring adenocarcinoma of the rectum/Phase 2
KRAS mutation negative peritoneal carcinomatosis/Phase 2
KRAS wild-type liver metastasis/Phase 2
KRAS wild-type metastatic colorectal cancer/Phase 1
KRAS wild-type metastatic colorectal cancer/Phase 1/Phase 2
KRAS wild-type metastatic colorectal cancer/Phase 2
KRAS wild-type positive colorectal cancer/Phase 1
KRAS wild-type positive colorectal cancer/Phase 1/Phase 2
KRAS wild-type positive colorectal cancer/Phase 2
large cell lung carcinoma/Phase 2
liver metastasis associated with BRAF mutation positive colorectal cancer/Phase 3
liver metastasis associated with KRAS mutation negative and NRAS mutation negative colorectal cancer/Phase 2

© 2000-2014 QIAGEN. All rights reserved.

			liver metastasis associated with KRAS wild-type metastatic colorectal cancer/Phase 2<br>liver metastasis associated with metastatic colorectal cancer/Phase 3<br>liver metastasis associated with RAS mutation positive colorectal cancer/Phase 3<br>lung cancer/Phase 1<br>lung cancer/Phase 1/Phase 2<br>lung cancer/Phase 2<br>lung squamous cell carcinoma/Phase 2<br>malignant carcinoid syndrome/Phase 2<br>malignant salivary gland tumor/Phase 2<br>malignant solid tumor/Phase 1<br>metastasis/Phase 2<br>metastasis/Phase 3<br>metastatic BRAF V600E mutation positive colorectal cancer/Phase 2<br>metastatic breast cancer/Phase 2<br>metastatic colorectal cancer/Phase 1/Phase 2<br>metastatic colorectal cancer/Phase 2<br>metastatic colorectal cancer/Phase 3<br>metastatic colorectal cancer/Unspecified phase<br>metastatic KRAS mutation negative colorectal adenocarcinoma/Phase 2<br>metastatic squamous cell cancer of the head and neck/Phase 2<br>neck neoplasm/Phase 2<br>non small cell lung adenocarcinoma/Phase 2<br>non-small cell lung cancer/Phase 1<br>non-small cell lung cancer/Phase 1/Phase 2<br>non-small cell lung cancer/Phase 2<br>pancreatic cancer/Phase 1<br>pancreatic cancer/Phase 1/Phase 2<br>pancreatic cancer/Phase 2<br>pleomorphic adenoma/Phase 2<br>primary KRAS mutation negative colorectal cancer/Phase 1/Phase 2<br>primary rectal adenocarcinoma/Phase 3<br>prostate cancer/Phase 1<br>prostate cancer/Phase 2<br>RAS mutation negative colorectal cancer/Phase 1/Phase 2<br>RAS mutation negative colorectal cancer/Phase 2<br>RAS mutation positive colorectal cancer/Phase 1/Phase 2<br>rectal adenocarcinoma/Phase 3<br>rectum cancer/Phase 1<br>rectum cancer/Phase 1/Phase 2<br>rectum cancer/Phase 2<br>recurrent breast cancer/Phase 2<br>renal cancer/Phase 1<br>skin squamous cell carcinoma/Phase 2<br>skin toxicity/Phase 2<br>solid tumor/Phase 1<br>solid tumor/Phase 2<br>squamous cell carcinoma of the esophagogastric junction/Phase 2<br>squamous cell carcinoma/Phase 2<br>stage 3a non small cell lung carcinoma/Phase 2<br>stage IV head and neck squamous cell carcinoma/Phase 2<br>stomach tumor/Phase 2<br>tumor/Phase 1<br>tumor/Phase 2<br>unresectable KRAS mutation negative adenocarcinoma of the rectum/Phase 1<br>unresectable KRAS mutation negative colon adenocarcinoma/Phase 1
PD 153035	EGFR	inhibitor	psoriasis/Approved
PF-06459988	EGFR	inhibitor	
PF-06747775	EGFR	inhibitor	EGFR exon 19 deletion positive EGFR T790M mutation positive non small cell lung cancer/Phase 1<br>EGFR exon 19 deletion positive non-small cell lung cancer/Phase 1<br>EGFR L858R mutation positive EGFR T790M mutation positive non small cell lung cancer/Phase 1<br>EGFR L858R mutation positive non-small cell lung cancer/Phase 1

© 2000-2014 QIAGEN. All rights reserved.

Drug Name	Targets	Actions	Brand Names	Indications/Status
posaconazole	CYP51A1	antagonist	Noxafil	acute myeloid leukemia/Phase 1<br>acute myeloid leukemia/Phase 3<br>acute myeloid leukemia/Phase 4<br>advanced malignant tumor/Phase 1<br>aspergillosis/Phase 3<br>Chagas disease/Phase 2<br>chronic granulomatous disease/Phase 2<br>chronic granulomatous disease/Phase 3<br>coccidioidomycosis/Phase 3<br>hematologic cancer/Phase 3<br>hyper-IgE syndrome/Phase 3<br>infection associated with acute myeloid leukemia/Phase 4<br>infection associated with myelodysplastic syndrome/Phase 4<br>infection/Approved<br>infection/Phase 1<br>infection/Phase 2<br>infection/Phase 3<br>infection/Phase 4<br>leukemia/Phase 2<br>leukopenia/Phase 3<br>mycosis/Phase 2<br>mycosis/Phase 3<br>myelodysplastic syndrome/Phase 3<br>myeloid leukemia/Phase 1<br>neutropenia/Phase 1<br>neutropenia/Phase 3<br>neutropenia/Phase 4<br>onychomycosis/Phase 2<br>oral candidiasis/Phase 2<br>oral candidiasis/Phase 3<br>oropharyngeal candidiasis/Approved
poziotinib	EGFR	inhibitor		advanced solid tumor/Phase 1<br>EGFR mutation positive adenocarcinoma of the lung/Phase 2<br>head and neck squamous cell carcinoma/Phase 2<br>HER2 positive gastric cancer/Phase 1/Phase 2<br>HER2-positive breast cancer/Phase 2<br>metastatic head and neck squamous cell carcinoma/Phase 2
RG 13022	EGFR	inhibitor		
RG 14620	EGFR	inhibitor		
rociletinib	EGFR	inhibitor		EGFR mutation positive EGFR exon 20 insertion activating mutation negative non-small cell lung cancer/Phase 1/Phase 2<br>EGFR mutation positive EGFR exon 20 insertion activating mutation negative non-small cell lung cancer/Phase 2<br>EGFR mutation positive EGFR exon 20 insertion activating mutation negative non-small cell lung cancer/Phase 3<br>EGFR T790M mutation negative EGFR exon 20 insertion mutation negative non-small cell lung cancer/Phase 2<br>EGFR T790M mutation positive EGFR exon 20 insertion activating mutation negative non-small cell lung cancer/Phase 2<br>EGFR T790M positive non-small cell lung cancer/Phase 1/Phase 2
sapitinib	EGFR	inhibitor		advanced solid tumor/Phase 1<br>ALK fusion negative EGFR-activating mutation negative non-small cell lung cancer/Phase 2<br>breast cancer/Phase 1<br>breast cancer/Phase 1/Phase 2<br>breast cancer/Phase 2<br>HER2-negative breast cancer/Phase 2<br>KRAS wild-type metastatic colorectal cancer/Phase 1<br>KRAS wild-type positive colorectal cancer/Phase 1<br>mammary tumor/Phase 1/Phase 2<br>mammary tumor/Phase 2<br>metastasis/Phase 1<br>tumor/Phase 1<br>tumor/Phase 1/Phase 2<br>tumor/Phase 2
selatinib	EGFR	inhibitor		HER2 positive metastatic breast cancer/Phase 1<br>HER2-positive breast cancer/Phase 1
sertaconazole	CYP51A1	inhibitor	Ertaczo	tinea pedis/Approved
sym004	EGFR	binder		KRAS wild-type metastatic colorectal cancer/Phase 2<br>metastatic colorectal cancer/Phase 1/Phase 2<br>solid tumor/Phase 1
TAK-285	EGFR	inhibitor		cancer/Phase 1

© 2000-2014 QIAGEN. All rights reserved.

Drug Name	Targets	Actions	Brand Names	Indications/Status
terconazole	CYP51A1	antagonist	Fungistat, Gyno-Terazol, Terazol, Terazol 3, Terazol 7, Tercospor	candidiasis/Approved
tesevatinib	EGFR	inhibitor		autosomal dominant polycystic kidney disease/Phase 1/Phase 2<br>breast cancer/Phase 1<br>cancer/Phase 1<br>esophageal cancer/Phase 1<br>gastric cancer/Phase 1<br>HER2 positive metastatic breast cancer/Phase 1/Phase 2<br>metastatic estrogen receptor positive HER2 positive breast cancer/Phase 1/Phase 2<br>non-small cell lung cancer/Phase 1<br>non-small cell lung cancer/Phase 2<br>non-small cell lung cancer/Phase 3
tioconazole	CYP51A1	antagonist	Fungibacid, GyneCure, Gyno-trosyd, Monistat 1, Trosyd, Trosyd AF, Trosyd J, Trosyl, Tz-3, Vagistat, Vagistat-1, Zoniden	gynecologic infection/Approved
triapine	RRM2	inhibitor		accelerated phase chronic myelogenous leukemia/Phase 1<br>accelerated phase chronic myelogenous leukemia/Phase 2<br>adult acute myeloid leukemia with 11q23 abnormalities/Phase 1<br>adult acute myeloid leukemia with inv(16)(p13.1q22)/Phase 1<br>adult acute myeloid leukemia with t(16;16)(p13;q22)/Phase 1<br>adult acute myeloid leukemia with t(8;21)(q22;q22)/Phase 1<br>adult Burkitt lymphoma/Phase 1<br>adult diffuse large-cell lymphoma/Phase 1<br>adult diffuse mixed-cell lymphoma/Phase 1<br>adult Hodgkin lymphoma/Phase 1<br>adult lymphoblastic lymphoma/Phase 1<br>adult solid tumor/Phase 1<br>adult T cell leukemia/Phase 1<br>angioimmunoblastic T-cell lymphoma/Phase 1<br>atypical chronic myeloid leukemia/Phase 2<br>cervical adenocarcinoma/Phase 2<br>cervical adenosquamous carcinoma/Phase 2<br>cervical cancer/Phase 1<br>cervical cancer/Phase 2<br>cervical squamous cell carcinoma/Phase 2<br>chronic B-cell leukemia/Phase 1<br>chronic eosinophilic leukemia/Phase 2<br>chronic myeloid leukemia/Phase 1<br>chronic myeloid leukemia/Phase 2<br>chronic myelomonocytic leukemia/Phase 2<br>cutaneous T-cell lymphoma/Phase 1<br>epithelial ovarian cancer/Phase 1<br>epithelial ovarian cancer/Phase 2<br>esophageal adenocarcinoma/Phase 2<br>esophageal cancer/Phase 2<br>essential thrombocythemia/Phase 2<br>extrahepatic bile duct cancer/Phase 2<br>gallbladder cancer/Phase 2<br>grade 1 follicular lymphoma/Phase 1<br>grade 2 follicular lymphoma/Phase 1<br>grade 3 follicular lymphoma/Phase 1<br>large-cell Ki-1 lymphoma/Phase 1<br>leukemia/Phase 1<br>lung cancer/Phase 2<br>male breast cancer/Phase 2<br>mantle cell lymphoma/Phase 1<br>marginal zone cell lymphoma/Phase 1<br>metastatic breast cancer/Phase 2<br>metastatic pancreatic cancer/Phase 2<br>myelodysplastic myeloproliferative neoplasm/Phase 1<br>myelodysplastic syndrome/Phase 1<br>myelodysplastic-myeloproliferative disease/Phase 1<br>myelofibrosis with myeloid metaplasia/Phase 2<br>non-small cell lung cancer/Phase 2<br>pancreatic adenocarcinoma/Phase 1<br>pancreatic cancer/Phase 1<br>pancreatic cancer/Phase 2<br>Philadelphia chromosome negative chronic myelogenous leukemia/Phase 2<br>PML-RARA mutation positive adult acute myeloid leukemia/Phase 1

© 2000-2014 QIAGEN. All rights reserved.
INGENUITY SYSTEMS

				polycythemia vera/Phase 2<br>primary peritoneal cancer/Phase 2<br>prostate cancer/Phase 2<br>recurrent adult acute lymphocytic leukemia/Phase 1<br>recurrent adult acute myeloid leukemia/Phase 1<br>recurrent adult diffuse large-cell lymphoma/Phase 1<br>recurrent breast cancer/Phase 2<br>recurrent grade 1 follicular lymphoma/Phase 1<br>recurrent grade 2 follicular lymphoma/Phase 1<br>recurrent grade 3 follicular lymphoma/Phase 1<br>relapsed mantle cell lymphoma/Phase 1<br>renal cancer/Phase 2<br>secondary acute myeloid leukemia/Phase 1<br>splenic marginal zone B-cell lymphoma/Phase 1<br>stage IV adult diffuse large cell lymphoma/Phase 1<br>vaginal cancer/Phase 1<br>vaginal cancer/Phase 2<br>vaginal squamous cell carcinoma/Phase 2<br>vulva cancer/Phase 1<br>Waldenstrom's macroglobulinemia/Phase 1
tyrphostin A30	EGFR	inhibitor		
vandetanib	EGFR	inhibitor	Caprelsa, Zactima	adenocarcinoma/Phase 1<br>adult solid tumor/Phase 1<br>advanced breast cancer/Phase 2<br>advanced cancer/Phase 1<br>ALK fusion negative EGFR-activating mutation negative non-small cell lung cancer/Phase 2<br>anaplastic astrocytoma/Phase 2<br>anaplastic oligodendroglioma/Phase 2<br>biliary tract cancer/Phase 1<br>biliary tract cancer/Phase 2<br>bladder cancer/Phase 2<br>breast cancer/Phase 1<br>breast cancer/Phase 2<br>cancer/Phase 1<br>cancer/Phase 2<br>cancer/Unspecified phase<br>carcinoma of ampulla of Vater/Phase 2<br>carcinoma/Phase 1<br>central nervous system tumor/Phase 1<br>colorectal cancer/Phase 1<br>colorectal cancer/Phase 2<br>colorectal carcinoma/Phase 1<br>colorectal tumor/Phase 1<br>differentiated thyroid cancer/Phase 3<br>esophageal cancer/Phase 1<br>fallopian tube cancer/Phase 2<br>fallopian tube neoplasm/Phase 2<br>gallbladder cancer/Phase 2<br>gastric cancer/Phase 2<br>gastrointestinal tract cancer/Unspecified phase<br>glioblastoma cancer/Phase 1<br>glioblastoma/Phase 1/Phase 2<br>glioblastoma/Phase 2<br>glioma cancer/Phase 1<br>glioma cancer/Unspecified phase<br>gliosarcoma cancer/Phase 1<br>gliosarcoma cancer/Phase 1/Phase 2<br>gliosarcoma cancer/Phase 2<br>head and neck cancer/Phase 1<br>head and neck cancer/Phase 2<br>head and neck squamous cell carcinoma/Phase 2<br>hepatocellular carcinoma/Phase 2<br>HER2-negative breast cancer/Phase 2<br>hereditary leiomyomatosis and renal cell cancer/Phase 1/Phase 2<br>invasive breast cancer/Unspecified phase<br>KIT-PDGFRA wild-type gastrointestinal stromal tumor/Phase 2<br>low-grade glioma/Phase 1<br>lung cancer/Phase 1<br>lung cancer/Phase 2<br>lung cancer/Phase 3<br>lymphocytic cancer/Phase 1<br>lymphoproliferative disorder/Phase 1<br>malignant glioma/Phase 1<br>malignant solid tumor/Phase 1<br>malignant urothelial neoplasm/Phase 2<br>mammary tumor/Phase 2<br>medullary thyroid cancer/Phase 1/Phase 2

© 2000-2014 QIAGEN. All rights reserved.

			medullary thyroid cancer/Phase 3
			mesothelioma/Phase 2
			metastasis/Phase 1
			metastasis/Phase 2
			metastatic breast cancer/Phase 1
			metastatic breast cancer/Phase 2
			metastatic paraganglioma/Phase 1
			metastatic pheochromocytoma/Phase 1
			multiple endocrine neoplasia 2b/Phase 1/Phase 2
			multiple endocrine neoplasia type 2A/Phase 1/Phase 2
			multiple myeloma/Phase 2
			neoplasia/Phase 2
			neuroblastoma/Phase 1
			non-small cell lung cancer/Phase 1
			non-small cell lung cancer/Phase 2
			non-small cell lung cancer/Phase 3
			ovarian cancer/Phase 1/Phase 2
			ovarian cancer/Phase 2
			ovarian tumor/Phase 2
			pancreatic cancer/Phase 1
			pancreatic cancer/Phase 2
			paraganglioma/Phase 1
			peritoneal tumor/Phase 2
			pheochromocytoma/Phase 1
			pleural effusion/Phase 2
			primary peritoneal cancer/Phase 2
			progressive medullary thyroid cancer/Approved
			prostate cancer/Phase 1
			prostate cancer/Phase 2
			recurrent cancer/Phase 1
			renal-cell carcinoma/Phase 1/Phase 2
			RET fusion positive non small cell lung cancer/Phase 2
			small cell lung cancer/Phase 2
			small intestine cancer/Phase 1
			solid tumor/Phase 1
			sporadic papillary renal cell carcinoma/Phase 1/Phase 2
			thyroid cancer/Phase 2
			thyroid cancer/Phase 3
			thyroid cancer/Phase 4
			thyroid gland tumor/Phase 2
			urethral cancer/Phase 2
varlitinib	EGFR	inhibitor	advanced cancer/Phase 1
			advanced cancer/Phase 1/Phase 2
			advanced solid tumor/Phase 1/Phase 2
			EGFR dysregulated solid tumor/Phase 1
			EGFR or HER2 expressing solid tumor/Phase 1
			EGFR T790M mutation negative solid tumor/Phase 1
			gastric cancer/Phase 2
			HER2 dysregulated solid tumor/Phase 1
			HER2 positive metastatic breast cancer/Phase 2
			HER2-positive breast cancer/Phase 1/Phase 2
			HER2-positive carcinoma of breast/Phase 1/Phase 2
			metastatic solid tumor/Phase 1
			pancreatic cancer/Phase 1/Phase 2
			solid tumor/Phase 1
			stomach tumor/Phase 2

© 2000-2014 QIAGEN. All rights reserved.

Drug Name	Targets	Actions	Brand Names	Indications/Status
voriconazole	CYP51A1	antagonist	Vfend	acquired immunodeficiency syndrome/Phase 3<br>acute myeloid leukemia/Phase 2<br>acute myeloid leukemia/Phase 3<br>adult solid tumor/Phase 2<br>allergic bronchopulmonary aspergillosis/Phase 2/Phase 3<br>aspergillosis/Approved<br>aspergillosis/Phase 1<br>aspergillosis/Phase 2<br>aspergillosis/Phase 2/Phase 3<br>aspergillosis/Phase 3<br>aspergillosis/Phase 4<br>bronchopulmonary aspergillosis/Approved<br>cancer/Unspecified phase<br>candidemia/Approved<br>candidemia/Phase 2<br>candidemia/Phase 3<br>candidemia/Phase 4<br>candidiasis/Approved<br>candidiasis/Phase 1<br>candidiasis/Phase 2<br>candidiasis/Phase 3<br>candidiasis/Phase 4<br>chronic B-cell leukemia/Phase 1<br>chronic obstructive pulmonary disease/Phase 4<br>corneal ulcer/Phase 3<br>cryptococcosis/Phase 4<br>cystic fibrosis/Phase 2<br>esophageal candidiasis/Approved<br>fungemia/Phase 3<br>graft-vs-host disease/Phase 1<br>graft-vs-host disease/Unspecified phase<br>hematologic cancer/Phase 3<br>infection/Phase 1<br>infection/Phase 2<br>infection/Phase 3<br>infection/Phase 4<br>leukemia/Phase 2<br>leukemia/Phase 3<br>leukemia/Unspecified phase<br>lymphocytic cancer/Phase 2<br>lymphocytic cancer/Phase 3<br>multiple myeloma/Phase 2<br>multiple myeloma/Unspecified phase<br>mycosis associated with infection/Approved<br>mycosis/Phase 1<br>mycosis/Phase 3<br>mycosis/Unspecified phase<br>myelodysplastic myeloproliferative neoplasm/Phase 2<br>myelodysplastic syndrome/Phase 2<br>myelodysplastic syndrome/Unspecified phase<br>neuroblastoma/Phase 2<br>neutropenia/Phase 1/Phase 2<br>neutropenia/Phase 2<br>neutropenia/Phase 3<br>renal cancer/Phase 2<br>renal failure/Phase 1/Phase 2<br>sarcoma/Phase 2

© 2000-2014 QIAGEN. All rights reserved.

Target Information - Overview of known drug targets in My Pathway

Showing 9 of 9 row(s) of Target data.

Target (Gene Symbol)	Entrez Gene Name	Location	Type	Drug(s)	Species
APOB	apolipoprotein B	Extracellular Space	transporter	mipomersen	Human, Mouse, Rat
CYP51A1	cytochrome P450, family 51, subfamily A, polypeptide 1	Cytoplasm	enzyme	betamethasone/clotrimazole, bifonazole, clotrimazole, econazole, fluconazole, itraconazole, ketoconazole, luliconazole, miconazole, oxiconazole, posaconazole, sertaconazole, terconazole, tioconazole, voriconazole	Human, Mouse, Rat
EGFR	epidermal growth factor receptor	Plasma Membrane	kinase	18F-PEG6-IPQA, ABT-414, AEE 788, afatinib, anti-EGFR monoclonal antibody GT-MAB 5.2-GEX, AP26113, ASP8273, avitinib, AZD3759, AZD9291, bevacizumab/cetuximab, bevacizumab/erlotinib, bevacizumab/panitumumab, BMS-599626, cabozantinib/erlotinib, canertinib, capecitabine/erlotinib, capecitabine/lapatinib, cetuximab, cetuximab/irinotecan, EGF816, EGFR antisense DNA, EGFR tyrosine kinase inhibitor, erlotinib, erlotinib/gemcitabine, erlotinib/vismodegib, gefitinib, HM61713, icotinib, IMGN289, irinotecan/panitumumab, lapatinib, lapatinib/letrozole, lapatinib/paclitaxel, lapatinib/pazopanib, lapatinib/trastuzumab, LY3164530, MEHD7945A, necitumumab, neratinib, nimotuzumab, panitumumab, PD 153035, PF-06459988, PF-06747775, poziotinib, RG 13022, RG 14620, rociletinib, sapitinib, selatinib, sym004, TAK-285, tesevatinib, tyrphostin A30, vandetanib, varlitinib	Human, Mouse, Rat
FN1	fibronectin 1	Extracellular Space	enzyme	L19-IL2 monoclonal antibody-cytokine fusion protein, ocriplasmin	Human, Mouse, Rat
GLS	glutaminase	Cytoplasm	enzyme	CB-839	Human, Mouse, Rat
GSK3B	glycogen synthase kinase 3 beta	Nucleus	kinase	enzastaurin	Human, Mouse, Rat
QPRT	quinolinate phosphoribosyltransferase	Cytoplasm	enzyme	atorvastatin/niacin, lovastatin/niacin, nicotinic acid, nicotinic acid/pioglitazone	Human, Mouse, Rat
RRM2	ribonucleotide reductase M2	Nucleus	enzyme	5-fluorouracil/gemcitabine, alemtuzumab/cyclophosphamide/fludarabine phosphate/mitoxantrone, alemtuzumab/cyclophosphamide/fludarabine phosphate/rituximab, alemtuzumab/fludarabine phosphate, bevacizumab/gemcitabine, bortezomib/cladribine/rituximab, capecitabine/docetaxel/gemcitabine, capecitabine/gemcitabine, chlorambucil/fludarabine phosphate, cladribine, cladribine/cytarabine/daunorubicin, cladribine/cytarabine/decitabine, cladribine/cytarabine/filgrastim, cladribine/cytarabine/filgrastim/idarubicin, cladribine/cytarabine/filgrastim/idarubicin/plerixafor, cladribine/cytarabine/filgrastim/mitoxantrone, cladribine/peginterferon alfa-2a, cladribine/rituximab, cyclophosphamide/fludarabine phosphate/mitoxantrone, cyclophosphamide/fludarabine phosphate/mitoxantrone/rituximab, cyclophosphamide/fludarabine phosphate/rituximab, cytarabine/filgrastim/fludarabine phosphate, cytarabine/fludarabine phosphate, cytarabine/fludarabine phosphate/oxaliplatin/rituximab, dexamethasone/fludarabine phosphate/mitoxantrone, dexamethasone/fludarabine phosphate/mitoxantrone/rituximab, docetaxel/gemcitabine, docetaxel/gemcitabine/vincristine, fludarabine phosphate, fludarabine phosphate/mitoxantrone, fludarabine phosphate/mitoxantrone/rituximab, fludarabine phosphate/rituximab, gallium nitrate, gemcitabine, gemcitabine/irinotecan, gemcitabine/oxaliplatin, gemcitabine/oxaliplatin/paclitaxel, gemcitabine/oxaliplatin/rituximab, gemcitabine/paclitaxel, gemcitabine/pemetrexed, gemcitabine/vinorelbine, hydroxyurea, L-asparaginase/gemcitabine/oxaliplatin, triapine	Human, Mouse, Rat
SMS	spermine synthase	Cytoplasm	enzyme	(±)-2-hydroxyoleic acid	Human, Mouse, Rat

Supporting References - References from which all of the Findings in the My Pathway were collected

© 2000-2014 QIAGEN. All rights reserved.
